# Design, Synthesis, and Biological Evaluation of Ferrocenyl–Cyclo-(Gly‑l‑Pro) Hybrids Sensitizing Multidrug-Resistant Cancer Cells to Anticancer Agents

**DOI:** 10.1021/acsmedchemlett.5c00256

**Published:** 2025-06-16

**Authors:** Andrzej Błauż, Karolina Rózga, Małgorzata Nosek, Anna Makal, Błażej Rychlik, Damian Plażuk

**Affiliations:** 1 Centre for Digital Biology and Biomedical Science - Biobank Lodz, Faculty of Biology and Environmental Protection, University of Lodz, ul. Pomorska 141/143, 90-236 Łódź, Poland; 2 Department of Organic Chemistry, Faculty of Chemistry, University of Lodz, ul. Tamka 12, 91-403 Łódź, Poland; 3 Laboratory for Structural and Biochemical Research, Biological and Chemical Research Centre, Department of Chemistry, University of Warsaw, ul. Zwirki i Wigury 101, 02-089 Warszawa, Poland; 4 Laboratory of Molecular Spectroscopy, Department of Organic Chemistry, Faculty of Chemistry, University of Lodz, ul. Tamka 12, 91-403 Łódź, Poland

**Keywords:** Multidrug resistance, ABCB1 inhibitors, ABCG2 inhibitors, Ferrocene, Bioorganometallic chemistry

## Abstract

Ferrocenyl–cyclo-(Gly-l-Pro) hybrids as novel inhibitors of ABCB1 and ABCG2 transporters were developed. These organometallic compounds were virtually nontoxic to colon cancer cells, their multidrug-resistant (MDR) variants, and normal fibroblasts. Derivatives bearing *o*-, *m*-, or *p*-ferrocenylphenyl groups significantly sensitized ABCB1- and ABCG2-overexpressing cells to chemotherapeutics such as vincristine, mitoxantrone, and doxorubicin, reducing IC_50_ values by up to 12.7- and 10.3-fold, respectively. Notably, (*S*,*Z*)-**4b**, (*S*,*Z*)-**4c**, and (*S*,*Z*)-**4d** showed the strongest effects. Drug combination studies revealed synergistic interactions, particularly in vincristine-, mitoxantrone-, and etoposide-resistant cells (synergy scores: 13.6–17.05). Accumulation assays confirmed ABC transporter inhibition, with (*S*,*Z*)-**4b** and (*S*,*Z*)-**4d** increasing intracellular retention of calcein and pheophorbide A up to 3.4- and 2.9-fold—comparable to those of verapamil and Ko143. Antibody-binding assays further indicated that these hybrids act as substrates of ABCB1 and ABCG2.

## Graphical abstract

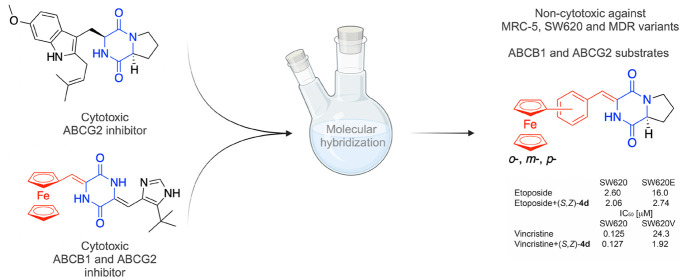

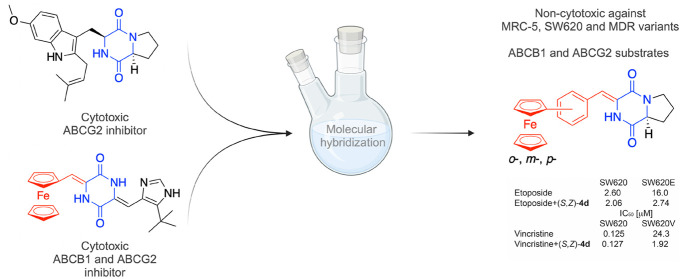

The use of chemotherapeutic agents in cancer treatment frequently results in tumor cell resistance against structurally diverse cytotoxic compounds—a phenomenon known as multidrug resistance (MDR)—which can ultimately cause treatment failure.^1^ A primary mechanism underlying MDR is the overexpression of ATP-binding cassette (ABC) transporters,^2,3^ which actively expel xenobiotics and secondary metabolites from cells, thereby protecting them from toxic substances. Nevertheless, the action of these transporters reduces the intracellular concentration of anticancer drugs, thus diminishing their therapeutic efficacy. Among several ABC transporters implicated in MDR, three key proteins^3^—P-glycoprotein (ABCB1),^4^ breast cancer resistance protein (ABCG2),^5^ and MDR-associated protein (ABCC1)^6^ —are quite frequently overexpressed in resistant tumors.^7^

Targeting ABC transporters with small-molecule inhibitors represents a promising strategy to overcome MDR. Over the recent years, research efforts have been devoted to identifying potent ABC protein inhibitors of low or negligible toxicity that efficiently sensitize multidrug-resistant tumor cells to anticancer drugs.^8,9^ Numerous structurally diverse compounds inhibit multidrug-resistant proteins,^8,10^ among which ABCB1 ^11^ and ABCG2 inhibitors^12^ have been particularly well-investigated, whereas ABCC1 inhibitors remain less explored.^13^ Several compounds exhibiting promising inhibitory activity have advanced to phase I or II clinical trials; however, none of them have been approved for clinical use.^14^ Nevertheless, the development of small-molecule inhibitors targeting ABC transporters remains a significant focus in MDR research.^15^ Remarkably, inhibitors containing the 2,5-piperazinedione scaffold^16,17^ have been extensively investigated as ABC inhibitors. Natural, synthetic, and semisynthetic compounds containing a 2,5-piperazinedione moiety—such as fumitremorgin C, Ko143,^18^ tryprostatin A^19^ (Figure 1), and others^20,21^ —have been identified as potential ABC protein inhibitors.

**Figure 1:**
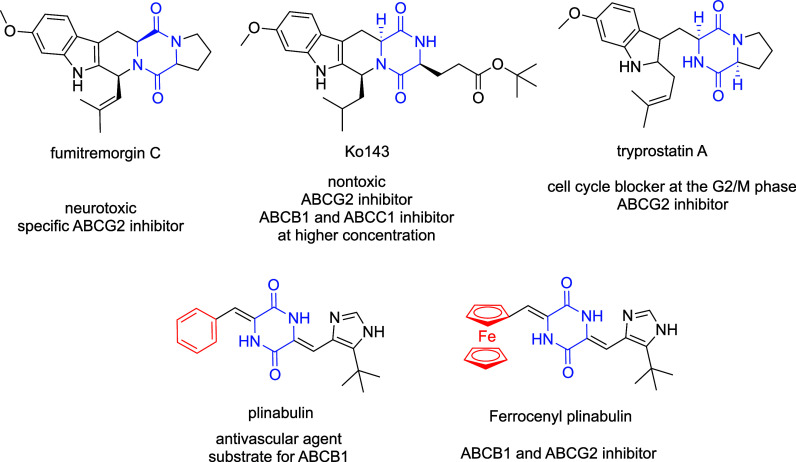
Structures of selected ABC inhibitors bearing the 2,5-piperazinedione moiety.

The development of new bioactive compounds often involves modifying the structures of existing ones to improve their properties.^22^ Although conventional organic groups provide ample opportunities for such modifications, there has been considerable interest in using organometallic compounds also to fine-tune the biological properties of active molecules in recent years. The introduction of an organometallic group into an organic compound can confer unique chemical properties (e.g., generation of reactive oxygen species or improved hydrophobic interactions) and distinct spatial geometry compared with organic groups, aiding in the design of novel drug candidates. The presence of an organometallic substituent, such as a ferrocenyl moiety, in the structure of organometallic compounds can result in improved activity of such molecules or the appearance of a new biological activity that the parental substance lacks.^23,24^ This approach has yielded numerous ferrocenyl analogs and derivatives of established drugs, including ferrocifen,^25^ which is active against both estrogen-dependent (MCF-7) and estrogen-independent (MDA-MB-231) breast cancer cells, and ferroquine,^26^ which exerts potent antimalarial effects. Furthermore, various ferrocenyl hybrids have demonstrated remarkable antitumor, antifungal, antimalarial, and antimicrobial activities.^23,27^ Some studies, including our own, suggest that introducing a ferrocenyl moiety into a bioactive structure can, in certain cases, overcome resistance to widely used therapies, including anticancer^28,29^ and antimalarial drugs.^30^

As depicted in Figure 1, some organic ABC transporter inhibitors feature a 2,5-piperazinedione scaffold with an indole substituent; however, these compounds often exhibit undesirable biological toxicity. Considering that none of the existing ABC transporter inhibitors have been approved for clinical use, there remains a critical need to develop new selective and nontoxic candidates. Inspired by the finding that plinabulin (**1**), which contains a phenyl group attached to a 2,5-piperazinedione core, is inactive against ABC-overexpressing cancer cells, whereas its ferrocenyl analog **2** exhibits inhibitory activity,^20^ we investigated the effect of ferrocenyl substitution in new organometallic hybrids designed by the molecular hybridization^31^ of the plinabulin ferrocenyl analog **2** with tryprostatin A (**3**), resulting in next-generation ABC transporter inhibitors **4a**–**e** (Figure 2). On the basis of these findings, we hypothesized that replacing the indole ring in tryprostatin A with a ferrocenyl group could improve ABC transporter inhibition in multidrug-resistant cancer cells and simultaneously reduce toxicity to normal cells, thus providing a promising new direction in MDR-targeted drug design.

**Figure 2:**
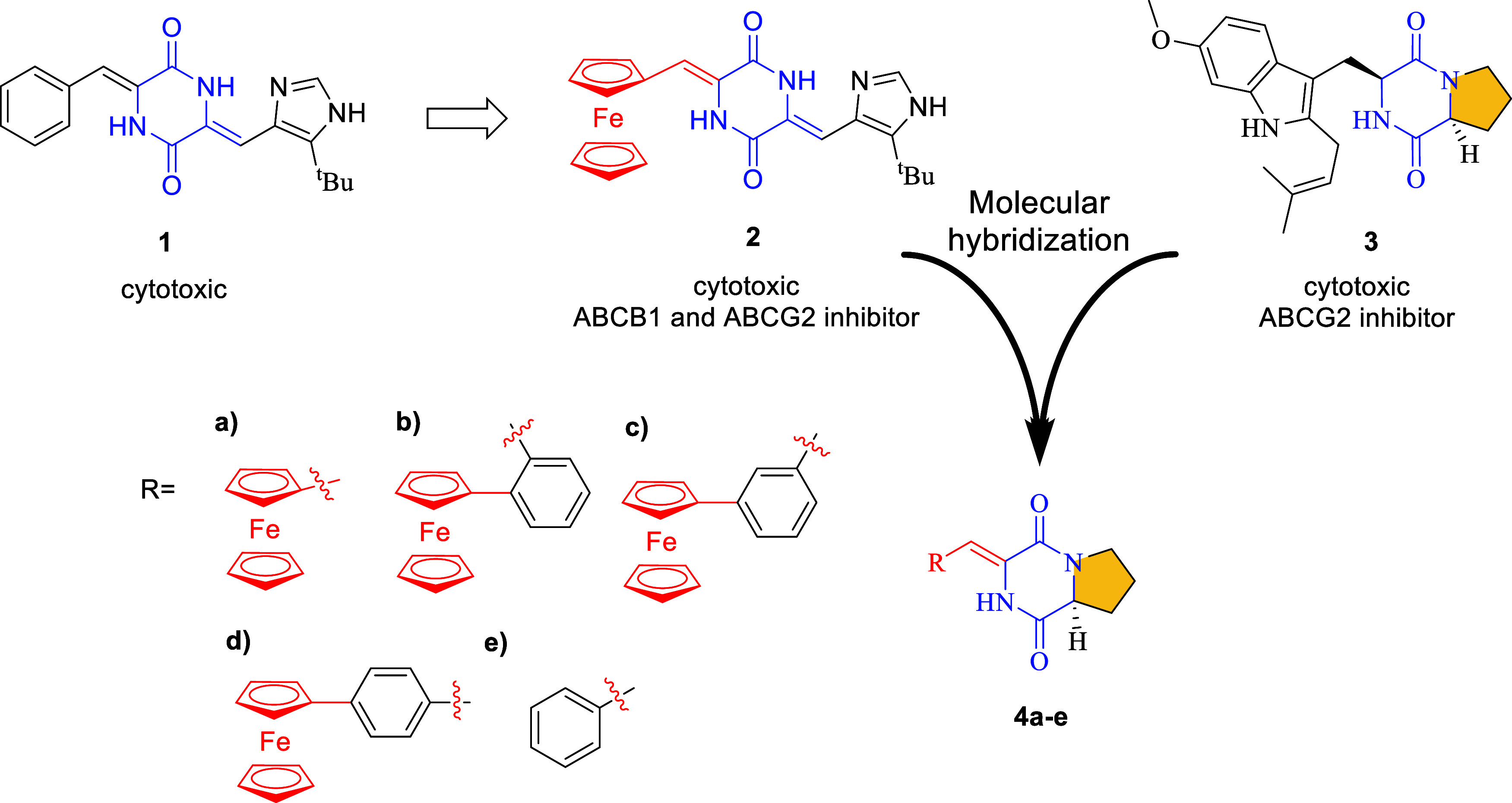
Structures of plinabulin (**1**), its ferrocenyl analog (**2**), tryprostatin A (**3**), and compounds **4a**–**e** investigated herein, designed by molecular hybridization of **2** and **3**.

We first attempted to synthesize compounds **4a**–**e** via the Knoevenagel condensation of *N*-acetyl-cyclo-(Gly-l-Pro) (**5**), which was prepared from cyclo-(Gly-l-Pro),^32^ with the corresponding aldehydes. This approach was analogous to that described for synthesizing the ferrocenyl analogs of plinabulin.^20^ However, reacting **5** with ferrocenecarboxaldehyde (**6a**) in DMF for 2 h at 90 °C in the presence of cesium carbonate resulted only in 33% recovery of unreacted starting **5**. Using other bases, such as potassium *tert*-butoxide, did not improve the outcome, as 90% of **5** was recovered. Microwave-assisted synthesis yielded **4d** in only 4.5% yield, whereas other aldehydes failed to react under these conditions (Scheme 1). The failure of the Knoevenagel condensation of **5** with **6a**, even under microwave-assisted conditions, may be attributed to a combination of factors, including the poor nucleophilicity of the methylene protons in **5**, steric hindrance from the diketopiperazine ring system, and the reduced electrophilicity of the formyl group in **6a**. These structural and electronic limitations likely prevented the efficient formation of the desired carbon–carbon double bond. The low reactivity observed for **5** with **6a** is consistent with previous reports describing similar challenges in the synthesis of ferrocenyl analogs of plinabulin via Knoevenagel condensation.^20^

**Scheme 1:**
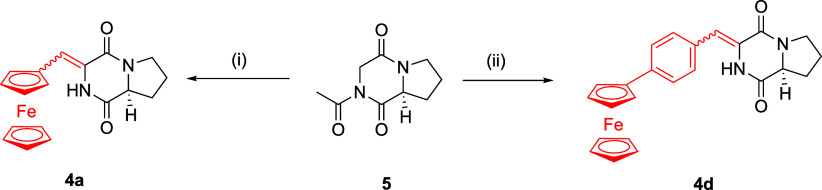
Attempts to Synthesize **4a** and **4d** via Knoevenagel Condensation of Peptide **5** with Aldehydes **6a** and **6d** ^a^

a Reagents and conditions: (i) ferrocenecarboxaldehyde (**6a**), Cs_2_CO_3_, DMF, 90 °C, 2h, no reaction; (ii) 4-ferrocenylbenzaldehyde (**6d**), Cs_2_CO_3_, DMF, 90 °C, 30 min, MW, 4.5%.

Zhang et al.^33^ recently reported an efficient synthesis of 2,5-diketopiperazines through the intramolecular nucleophilic cycloaddition of amides to ynamides. We adopted this procedure to prepare **4a**–**e** from the corresponding propynoic acids **9a**–**e** (where **9e** is commercially available), which were synthesized in several steps from aldehydes **6a**–**d**. First, the reaction of **6a**–**d** with carbon tetrabromide and triphenylphosphine yielded 1,1-dibromoethenes **7a**–**d**, which were converted into acetylenes **8a**–**d** in 62–98% yield by treatment with *n*-BuLi at −78 °C. Subsequent reaction of these acetylenes with *n*-BuLi followed by carbon dioxide furnished acids **9a**–**d** in 46–82% yield. Next, **9a**–**e** coupled with l-prolinamide using 1-hydroxybenzotriazole (HOBt) and *N*,*N*′-diisopropylcarbodiimide (DIC) in *N*,*N*-dimethylformamide (DMF) at room temperature (23 °C) for 18 h, which yielded ynamides **10a**–**e**. These compounds were isolated as rotamer mixtures, as confirmed by the NMR spectra. Cyclization of ynamides **10a**–**e** under Zhang’s conditions (80 °C for 12 h in a DMF/toluene mixture) gave only a 12% yield of **4e** after 48 h, with 33% of **10e** recovered. Similarly, the ferrocenyl analogue **4a** was isolated in only 4% yield, and no formation of **4b**–**d** was detected. Extending the reaction time or increasing the temperature resulted in decomposition of the starting material. However, we found that performing the cyclization under microwave conditions—using 30 min cycles at 120 °C for a total of 1–2.5 h—afforded **4a**–**e** in total yields of 29.5–77.5% (Scheme 2).

**Scheme 2:**
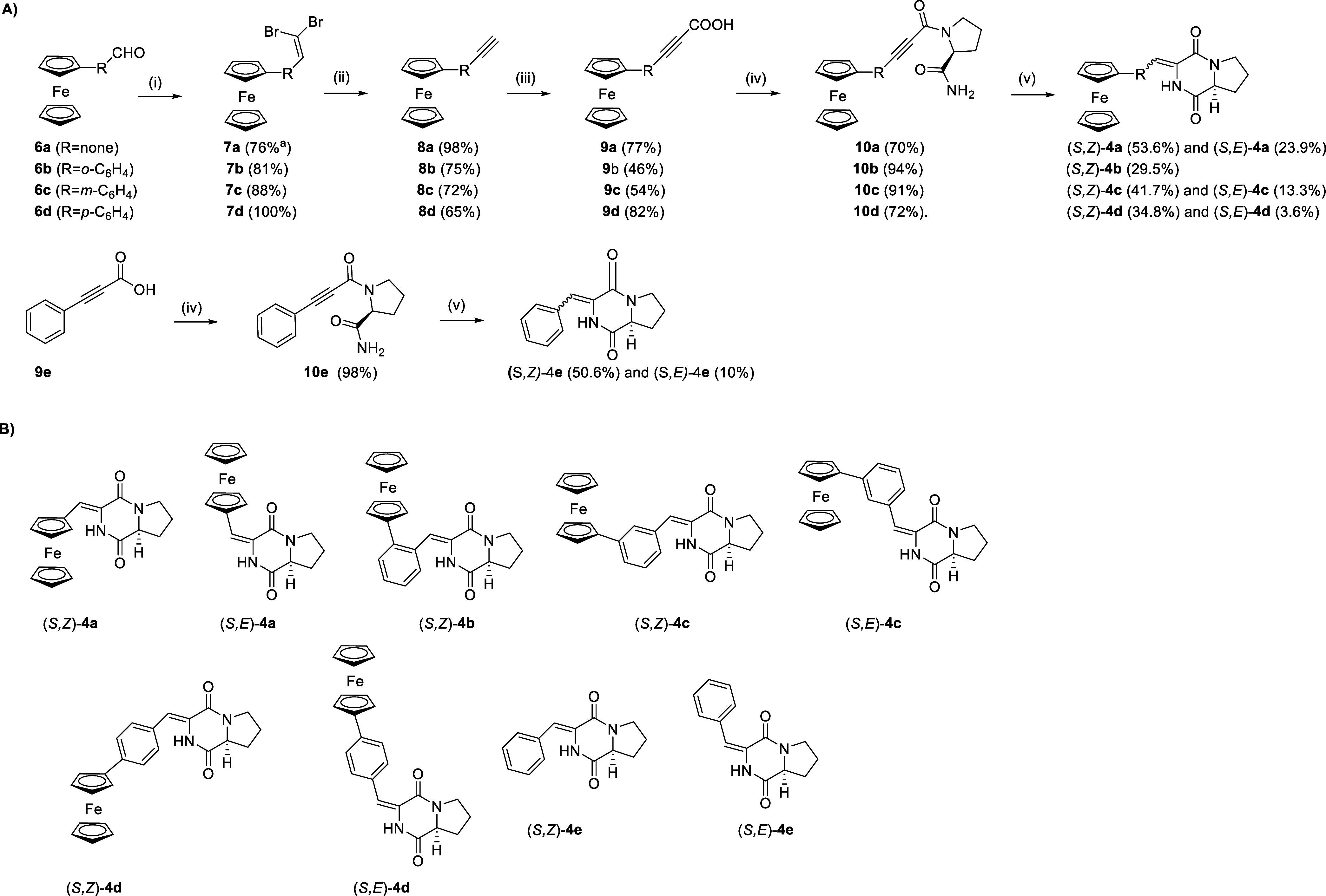
(A) Synthesis^a^ and (B) Structures of Compounds **4a**–**e**

a Reagents and conditions: (i) CBr_4_, PPh_3_, DCM, RT, 10 min; (ii) *n*-BuLi, THF, −78 °C, 1 h; (iii) *n*-BuLi, THF, −78 °C, 1 h, then CO_2_, −78 °C to RT, 1 h; (iv) (*S*)-prolinamide, DIC, HOBt, DMF, RT, 18 h; (v) Me_3_P, Glu, DMF/toluene (1:6), 120 °C, MW, up to 150 min.

All final compounds were obtained as mixtures of enantiomers with a predominance of the (*S*,*Z*) isomer, as determined by chiral HPLC: (*Z*)-**4a** ((*S*,*Z*)-**4a**:(*R*,*Z*)-**4a** ratio of 78:22), (*Z*)-**4b** ((*S*,*Z*)-**4b**:(*R*,*Z*)-**4b** ratio of 90:10), (*Z*)-**4c** ((*S*,*Z*)-**4c**:(*R*,*Z*)-**4c** ratio of 74:26), (*Z*)-**4d** ((*S*,*Z*)-**4d**:(*R*,*Z*)-**4d** ratio of 70:30), and (*Z*)-**4e** ((*S*,*Z*)-**4e**:(*R*,*Z*)-**4e** ratio of 74:26). In all cases, the (*S*)-configured stereoisomer was predominant; however, to facilitate the reading of the paper, we refer only to the configuration of the major enantiomer present in the isolated mixture. The stereochemical composition, partial racemization, and solid-state structures of the synthesized compounds were confirmed by chiral HPLC, selROESY experiments, and X-ray crystallography; full details and supporting figures are provided in the Supporting Information (Figures S1–S24 and Table S1).

Effective ABC transporter inhibitors should exhibit low intrinsic toxicity to be suitable for therapeutic use. To evaluate this aspect, we investigated the viability of SW620 colon cancer cells and their multidrug-resistant variants (overexpressing ABCB1, ABCC1, and ABCG2)^34^ exposed to 1 and 10 μM **4a**–**e** using a neutral red uptake assay (Table 1). In general, compounds **4a**–**e** exerted little or no cytotoxic effect on either SW620 or multidrug-resistant sublines even at 10 μM, with the remarkable exception of SW620C (cisplatin-selected variant of SW620) cells, which turned out to be relatively susceptible toward ferrocenyl **4a**–**c**. These compounds bear ferrocenyl or *o*- or *m*-ferrocenylphenyl substituents, suggesting that the position of the ferrocenyl group may influence biological activity. Among them, (*S*,*Z*)-**4c** (meta-substituted) showed the strongest cytotoxic effect against SW620C cells (survival rate = 23 ± 1.4% at 10 μM), whereas para-substituted (*S*,*Z*)-**4d** was significantly less active. This trend may reflect steric or electronic contributions to cellular uptake or interaction with intracellular targets. Additionally, comparison between geometric isomers of compound **4c** revealed that only the *Z* isomer exhibited cytotoxicity toward SW620C cells. The *E* isomer was essentially inactive (survival rate = 87 ± 1.1%), suggesting that the stereochemistry of the vinyl group plays a role in the biological activity. However, since *E*/*Z* isomeric data are currently available only for compound **4c**, this hypothesis requires validation. To quantify this effect, IC_50_ values were determined (Table 2). Most compounds showed IC_50_ values above 100 μM, with the exception of (*S*,*Z*)-**4a**, (*S*,*Z*)-**4b**, and (*S*,*Z*)-**4c**, which exhibited low-micromolar IC_50_ values in SW620C cells. In contrast, (*S*,*E*)-**4c** had a 13-fold higher IC_50_ value, further supporting the relevance of stereochemistry. Additionally, (*S*,*Z*)-**4b** showed moderate activity in SW620M, -V, and -Mito variants (IC_50_ ≈ 50 μM). These results indicate that both the presence and spatial arrangement of the ferrocenyl moiety significantly affect the cytotoxic properties of the compounds, particularly in resistant cell lines. Importantly, none of the tested compounds—except (*S*,*Z*)-**4c**—significantly reduced the viability of normal fibroblasts (MRC-5), confirming their generally low intrinsic toxicity.

**Table 1:** Survival Rates of SW620 Parental and Multidrug-Resistant Cell Lines after 72 h Exposure to 1 or 10 μM **4a**–**e** (*n* = 3); Survival Data for MRC-5 Fibroblasts Exposed to 10 μM Tryprostatin A (TrpA) and **4a**–**e** Are Also Included

		survival rate [% ± SD]							
cell line	conc. (μM)	(*S*,*Z*)-**4a**	(*S*,*Z*)-**4b**	(*S*,*Z*)-**4c**	*(S,E)*-**4c**	(*S*,*Z*)-**4d**	(*S*,*Z*)-**4e**	(*S*,*E*)-**4e**	TrpA
MRC-5	10	106 ± 13	92 ± 8.7	70 ± 7.9	110 ± 10	101 ± 7	102 ± 6	117 ± 11	97 ± 11
SW620	1	95 ± 2.2	91 ± 8.0	97 ± 2.5	95 ± 3.4	97 ± 3.0	94 ± 5.4	96 ± 3.4	ND^a^
	10	88 ± 6.0	84 ± 13	78 ± 8.1	95 ± 6.0	97 ± 2.1	92 ± 5.0	96 ± 2.2	ND
SW620C	1	78 ± 1.8	83 ± 4.2	80 ± 3.5	84 ± 0.7	96 ± 3.6	95 ± 2.1	96 ± 0.8	ND
	10	**54 ± 3.1**	**57 ± 1.1**	**23 ± 1.4**	87 ± 1.1	90 ± 1.6	95 ± 1.0	95 ± 2.5	ND
SW620D	1	96 ± 4.8	97 ± 1.0	96 ± 2.4	95 ± 1.5	92 ± 0.8	96 ± 0.7	96 ± 2.4	ND
	10	93 ± 1.1	87 ± 3.0	91 ± 0.7	95 ± 4.5	96 ± 0.9	94 ± 2.9	95 ± 0.7	ND
SW620E	1	93 ± 0.8	93 ± 1.2	98 ± 1.5	95 ± 2.1	97 ± 0.8	94 ± 1.9	96 ± 2.5	ND
	10	94 ± 3.0	99 ± 0.8	96 ± 1.7	91 ± 0.8	94 ± 2.5	96 ± 4.4	99 ± 0.9	ND
SW620M	1	93 ± 0.9	97 ± 0.8	94 ± 1.0	93 ± 1.8	99 ± 0.8	96 ± 4.8	93 ± 0.3	ND
	10	98 ± 2.3	96 ± 3.5	92 ± 1.3	96 ± 4.6	95 ± 1.5	95 ± 4.0	95 ± 3.4	ND
SW620V	1	94 ± 0.7	92 ± 0.9	96 ± 1.6	96 ± 1.8	94 ± 2.9	98 ± 0.9	95 ± 2.9	ND
	10	94 ± 2.5	95 ± 2.4	95 ± 3.6	94 ± 0.8	95 ± 3.2	99 ± 1.4	95 ± 3.3	ND
SW620Mito	1	98 ± 2.0	85 ± 1.0	97 ± 0.9	94 ± 3.5	98 ± 0.9	92 ± 1.3	94 ± 0.7	ND
	10	90 ± 3.1	85 ± 1.9	96 ± 3.4	94 ± 1.1	93 ± 1.1	94 ± 1.8	96 ± 0.8	ND

a ND: not determined.

**Table 2:** Cytotoxic Activity (IC_50_) of **4a**–**e** in Human Colorectal Cancer Cell Lines and Cisplatin- (SW620C), Doxorubicin- (SW620D), Etoposide- (SW620E), Methotrexate- (SW620M), Vincristine- (SW620V), and Mitoxantrone-Resistant (SW620Mito) Variants^a^

	IC_50_ [μM] (95% CI)						
compound	SW620	SW620C	SW620D	SW620E	SW620M	SW620V	SW620Mito
(*S*,*Z*)-**4a**	>100	15.1 (13.3–17.2)	>100	>100	>100	>100	>100
(*S*,*Z*)-**4b**	>100	12.0 (9.87–14.5)	>100	>100	52.2 (43.6–63.6)	48.8 (40.7–59.5)	44.9 (34.3–62.4)
(*S*,*Z*)-**4c**	72.4 (58.2–94.8)	4.77 (3.83–5.87)	91.2 (79.4–109)	93.6 (87.7–100)	88.2 (77.4–100)	>100	80.9 (73.1–89.6)
(*S*,*E*)-**4c**	>100	62.4 (49.1–79.3)	>100	>100	>100	>100	>100
(*S*,*Z*)-**4d**	>100	>100	>100	>100	>100	>100	>100
(*S*,*Z*)-**4e**	>100	>100	>100	>100	>100	>100	>100
(*S*,*E*)-**4e**	>100	>100	>100	>100	>100	>100	>100

a Exposure time = 72 h. The IC_50_ values and corresponding 95% confidence intervals are shown in parentheses (*n* = 3).

We investigated the potency of **4a**–**e** to inhibit ABC transporters (ABCB1, ABCC1, and ABCG2) by determining their enhancement of reversal potency (ERP) values. ERP represents fold changes in the IC_50_ values of anticancer drugs in multidrug-resistant cancer sublines (ABCB1-overexpressing SW620D, SW620E, and SW620V; ABCC1-overexpressing SW620M; ABCG2-overexpressing SW620C and SW620Mito) exposed to a combination of the investigated compounds and anticancer agents at equimolar concentrations, compared with values obtained with drugs alone (Tables S2 and S3 and Figure 3).

**Figure 3:**
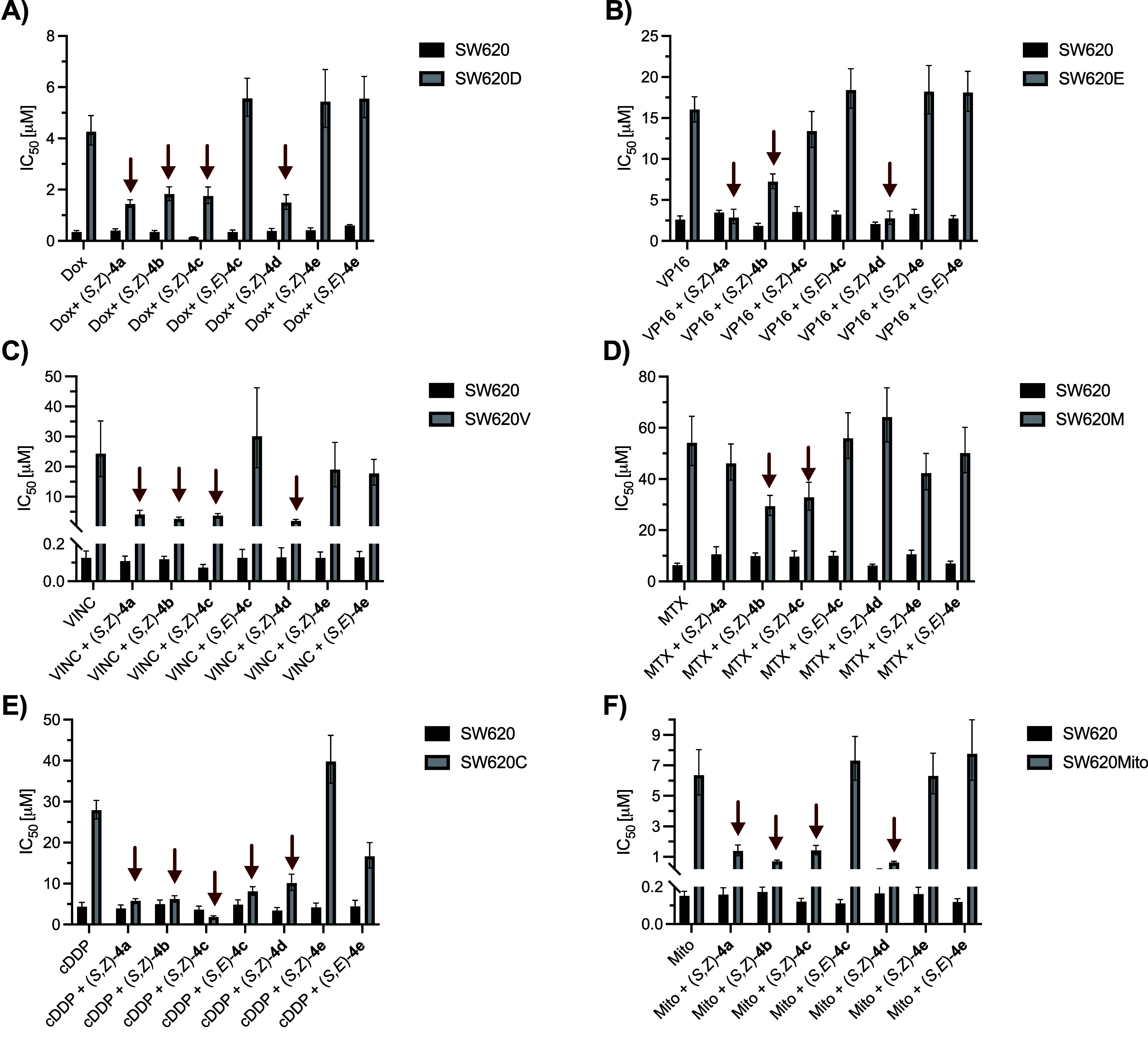
Effect of coadministration of **4a**–**e** and anticancer drugs at equimolar concentrations, expressed as IC_50_ value of drugs in SW620 and MDR versions of SW620 cancer cell lines (*n* = 3). The most active compounds are indicated with red arrows. (A) SW620D treated with Dox and **4a**–**e**; (B) SW620E treated with VP16 and **4a**–**e**; (C) SW620V treated with Vinc and **4a**–**e**; (D) SW620M treated with MTX and **4a**–**e**; (E) SW620C treated with cDDP and **4a**–**e**; and (F) SW620Mito treated with Mito and **4a**–**e**.

Of the seven compounds, four were found to be sensitizers of multidrug-resistant cells, although the extent of sensitization depended on both the specific cell line variant and the inhibitor. For instance, among ABCB1-overexpressing cells, (*S*,*Z*)-**4a** and (*S*,*Z*)-**4d** were remarkably effective in SW620E cells (ERP = 5.61 and 5.84, respectively), whereas in SW620V cells, the effects of (*S*,*Z*)-**4b** and (*S*,*Z*)-**4d** were the most pronounced (ERP = 8.57 and 12.7, respectively). Among ABCG2-overexpressing cells, cotreatment was most effective in SW620Mito cells treated with a combination of mitoxantrone and (*S*,*Z*)-**4b** or (*S*,*Z*)-**4d** (ERP = 9.11 and 10.3, respectively). The pronounced reversal effect was observed also for (*S*,*Z*)-**4c**, which reached an ERP value of 15.2 in SW620C cells treated with cisplatin. In contrast, its (*S*,*E*)-**4c** geometric isomer showed negligible reversal potency, underscoring the significance of stereochemistry at the vinyl group in determining the activity. Notably, (*S*,*Z*)-**4c** also exhibited substantial intrinsic cytotoxicity toward drug-sensitive SW620 cells, unlike the other tested compounds. In aggregate, these data suggest that the high ERP value observed for the (*S*,*Z*)-**4c** + cDDP combination may be attributed not solely to multidrug-resistance protein inhibition but rather to a combination of effects, including the inherent cytotoxicity of (*S*,*Z*)-**4c**, an additive interaction with cDDP, and other phenomena. As discussed before,^34^ exposure to cisplatin leads to transcriptionally regulated activation of DNA repair systems, which is considered to be the dominant mechanism of cDDP resistance. Thus, ABCG2-overexpression in SW620C cells may be a side effect of this activation, and the antiproliferative potential of (*S*,*Z*)-**4c** may result from inhibition of other, yet unknown, molecular targets. The effects in ABCC1-overexpressing SW620M cells were rather mild, although both (*S*,*Z*)-**4b** and (*S*,*Z*)-**4c** affected methotrexate resistance in these cells (ERP = 1.84 and 1.65, respectively).

The results clearly demonstrate that along with low cytotoxicity, the ferrocene compounds **4a**–**d** exert superior ability to reverse multidrug resistance compared to the phenyl analogue **4e**. The strongest effects were observed for derivatives bearing *o*-, *m*-, and *p*-ferrocenylphenyl substituents. This enhancement may be attributed to increased lipophilicity and improved interactions with membrane proteins resulting from the hydrophobic nature of the ferrocene moiety.

In an ideal setting, the effect of cell exposure to a combination of a chemotherapeutic and an MDR inhibitor should be synergistic. To evaluate the potential interactions between putative ABC inhibitors and anticancer drugs, we used the Bliss model via the SynergyFinder web application.^35,36^ The calculated synergy scores (Table 3) and the corresponding synergy maps (Figure 4) revealed synergy across all multidrug-resistant sublines, except for methotrexate-resistant SW620M cells. These findings indicate that the combination treatment was more effective than individual drug exposure. The highest synergy scores identified an optimal concentration range where the combination exerted its maximal effect. Among the tested compounds, ferrocene-containing (*S*,*Z*)-**4a**–**d** were the most potent agents that effectively sensitized ABCG2- and ABCB1-overexpressing cells. In cisplatin-resistant SW620C cells, the low or lack of synergy observed for most compounds may be due to the additive effects of cisplatin and **4** rather than a true synergistic interaction. Nevertheless, (*S*,*Z*)-**4c** and (*S*,*Z*)-**4d** exhibited significant synergy in SW620C cells. Remarkably, coadministration of **4a**–**e** with anticancer drugs rarely increased cytotoxicity in nonresistant SW620 cells, indicating a potentially favorable therapeutic window for these organometallic inhibitors.

**Table 3:** Synergy Scores and the Most Synergistic Area Scores Calculated Using SynergyFinder; Synergy Scores in the SW620 Cancer Cell Line Are Provided in Parentheses^a^

drug	additive	cell line	synergy score	most synergistic area score
cisplatin	(*S*,*Z*)-**4a**	SW620C (SW620)	–2.62 (−4.74)	4.33
	(*S*,*Z*)-**4b**		–2.78 (−5.00)	0.00
	(*S*,*Z*)-**4c**		**5.46** (−4.15)	**19.80**
	(*S*,*E*)-**4c**		0.39 (−4.66)	8.86
	(*S*,*Z*)-**4d**		**6.03** (−2.46)	**13.42**
	(*S*,*Z*)-**4e**		–3.14 (−3.94)	6.66
	(*S*,*E*)-**4e**		–2.78 (−4.17)	3.89
doxorubicin	(*S*,*Z*)-**4a**	SW620D (SW620)	**6.30** (−4.15)	**20.51**
	(*S*,*Z*)-**4b**		**3.96** (−2.67)	**11.62**
	(*S*,*Z*)-**4c**		**5.41** (5.91)	**11.32**
	(*S*,*E*)-**4c**		–3.01 (−1.89)	1.68
	(*S*,*Z*)-**4d**		**7.37** (−3.76)	**17.81**
	(*S*,*Z*)-**4e**		–8.70 (−3.90)	0.00
	(*S*,*E*)-**4e**		–6.63 (−4.78)	0.00
etoposide	(*S*,*Z*)-**4a**	SW620E (SW620)	**12.44** (−4.45)	**28.26**
	(*S*,*Z*)-**4b**		**3.32** (−5.23)	**14.42**
	(*S*,*Z*)-**4c**		**3.01** (−5.01)	**21.52**
	(*S*,*E*)-**4c**		–4.06 (−4.80)	1.22
	(*S*,*Z*)-**4d**		**16.02** (0.64)	**26.95**
	(*S*,*Z*)-**4e**		–2.56 (−3.96)	2.22
	(*S*,*E*)-**4e**		–3.93 (−1.15)	0.19
methotrexate	(*S*,*Z*)-**4a**	SW620M (SW620)	–3.78 (−5.99)	3.56
	(*S*,*Z*)-**4b**		–5.03 (3.26)	1.57
	(*S*,*Z*)-**4c**		–1.36 (−4.92)	2.23
	(*S*,*E*)-**4c**		–2.83 (−4.31)	0.00
	(*S*,*Z*)-**4d**		–5.20 (−2.64)	0.00
	(*S*,*Z*)-**4e**		–3.16 (−5.58)	1.17
	(*S*,*Z*)-**4e**		–5.69 (−3.81)	3.78
mitoxantrone	(*S*,*Z*)-**4a**	SW602Mito (SW620)	**7.86** (−1.66)	**16.82**
	(*S*,*Z*)-**4b**		**11.11** (−3.17)	**19.63**
	(*S*,*Z*)-**4c**		**10.09** (0.48)	**18.93**
	(*S*,*E*)-**4c**		–4.74 (0.86)	0.67
	(*S*,*Z*)-**4d**		**17.05** (−1.93)	**27.66**
	(*S*,*E*)-**4e**		–0.12 (−3.41)	4.35
	(*S*,*Z*)-**4e**		–3.17 (1.87)	0.00
vincristine	(*S*,*Z*)-**4a**	SW620V (SW620)	**4.27** (−0.36)	**15.88**
	(*S*,*Z*)-**4b**		**7.15** (5.77)	**27.24**
	(*S*,*Z*)-**4c**		**9.96** (2.61)	**26.87**
	(*S*,*Z*)-**4c**		–4.34 (−0.97)	0.83
	(*S*,*Z*)-**4d**		**13.60** (−1.29)	**24.93**
	(*S*,*E*)-**4e**		0.36 (−1.47)	5.58
	(*S*,*E*)-**4e**		–1.45 (−1.07)	2.19

a Synergy score < −10: likely antagonistic interactions. Synergy score between −10 and 10: likely additive interactions. Synergy score >10: likely synergistic interactions.

**Figure 4:**
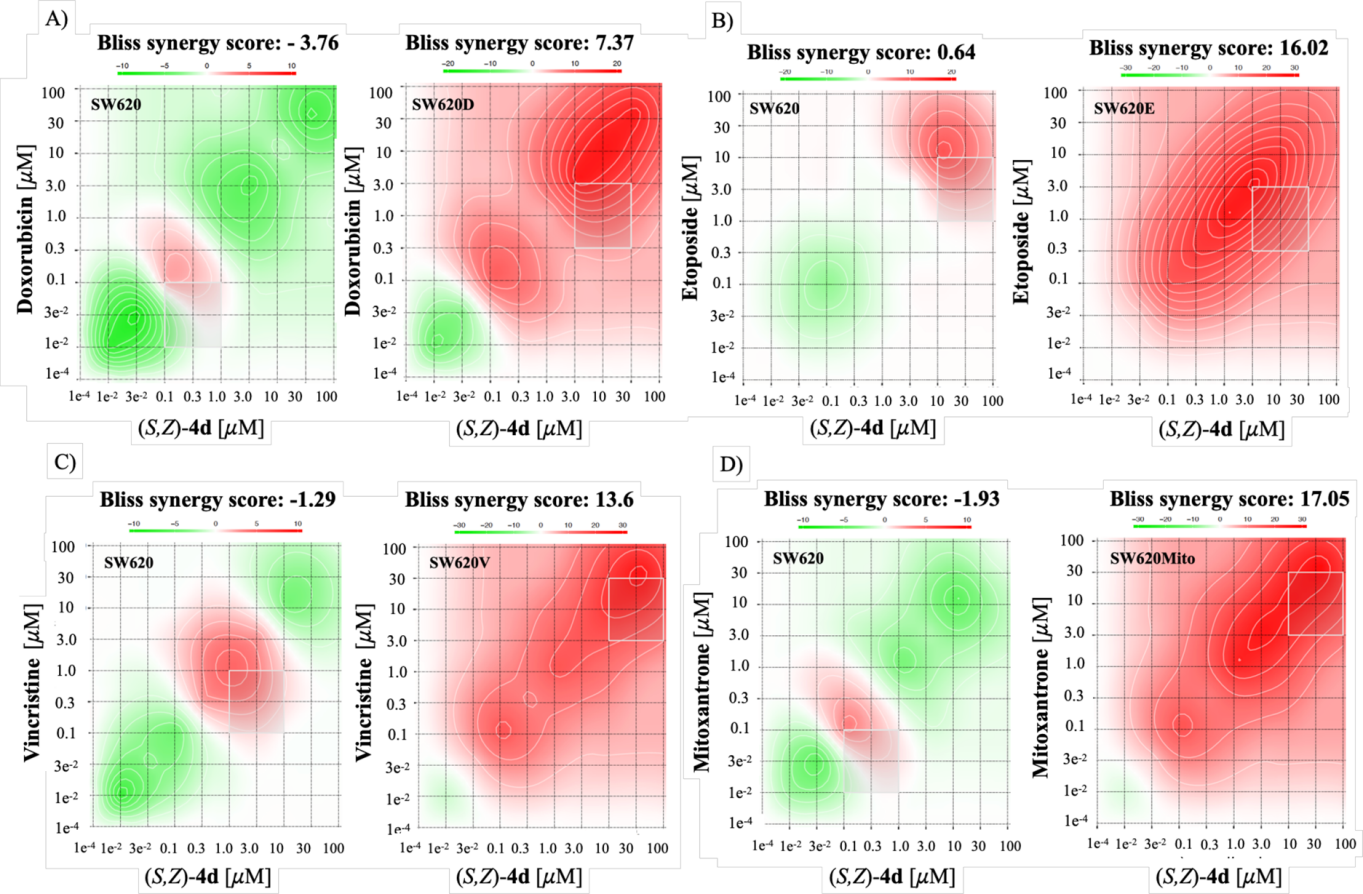
Most synergistic areas, calculated by SynergyFinder using the Bliss model, of SW620 cells (left) and their multidrug-resistant variants (right) treated with a combination of an anticancer drug and (*S*,*Z*)-**4d** at various concentrations: (A) SW620D, (B) SW620E, (C) SW620V, and (D) SW620Mito. Synergy score < −10: likely antagonistic interactions. Synergy score between −10 and 10: likely additive interactions. Synergy score >10: likely synergistic interactions.

To confirm the hypothesis of direct interaction between putative ABC protein inhibitors and transporter molecules, we conducted a series of experiments based on fluorescent probe transport as described previously.^20^ None of the compounds affected 2′,7′-bis­(2-carboxyethyl)-5-(and-6)-carboxyfluorescein (BCECF) efflux out of SW620M cells, indicating the lack of interaction between the putative inhibitors and ABCC1 (Figure 5A). (*S*,*Z*)-**4b**, (*S*,*Z*)-**4d**, and to a lesser extent (*S*,*Z*)-**4a** also efficiently inhibited ABCB1 activity as assessed using the calcein accumulation assay (Figure 5B). A similar inhibition pattern was observed in the pheophorbide A (PheA) accumulation assay of SW620Mito cells, indicative of ABCG2 activity, with a remarkable exception for (*S*,*Z*)-**4c**, which could inhibit PheA export (Figure 5C). As anticipated, none of the newly synthesized compounds affected dye accumulation in drug-sensitive SW620 cells (data not shown).

**Figure 5:**
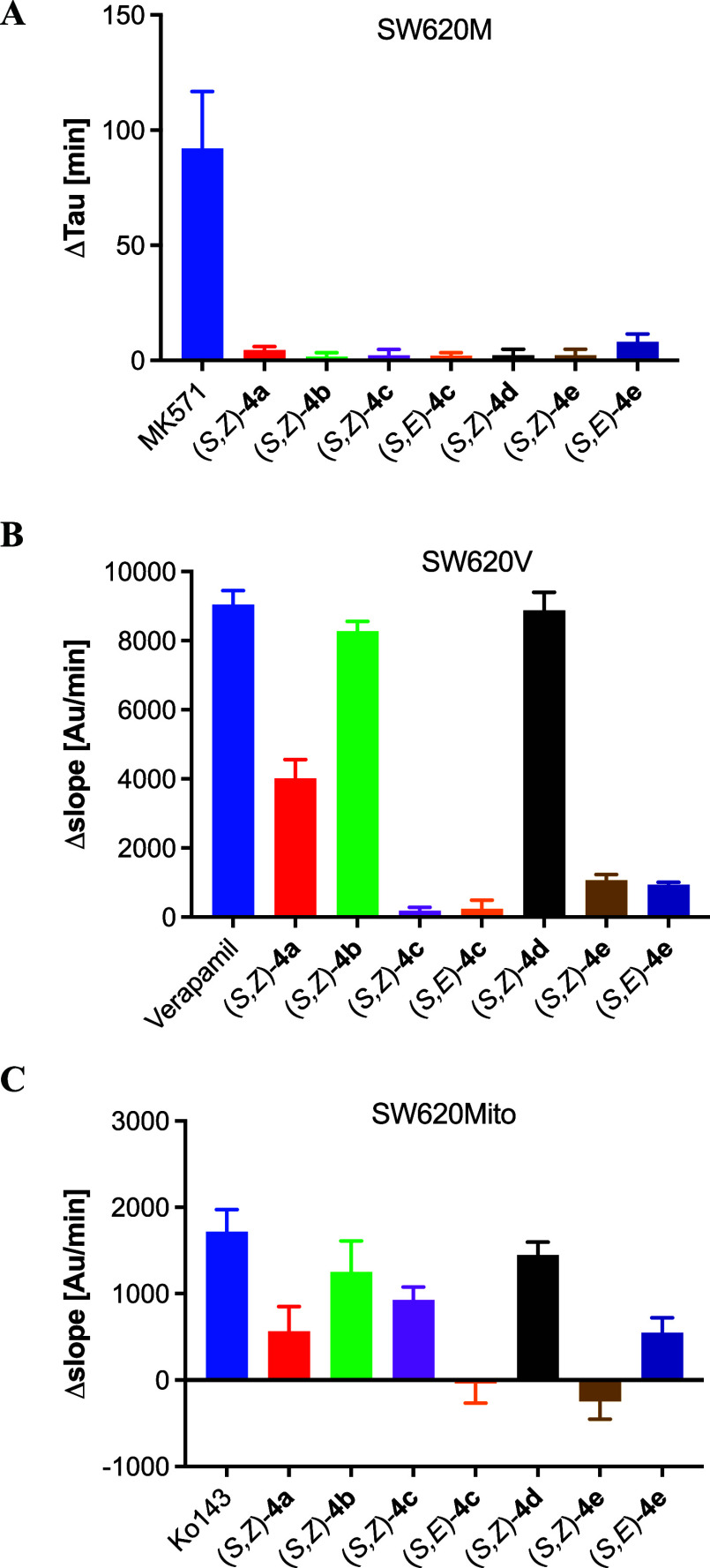
Effects of **4a**–**e** on the retention of BCECF and the accumulation of calcein and pheophorbide A in different cell lines overexpressing distinct ABC transporters: (A) ABCC1, (B) ABCB1, and (C) ABCG2. Established inhibitors were used as positive controls (*n* = 3).

To obtain deeper insight into the direct interactions between the investigated inhibitors and transporter protein molecules, we used two commercially available antibodies, UIC-2 and 5D3, which recognize the active forms of ABCB1 and ABCG2, respectively. These antibodies specifically recognize epitopes that are exposed when the transporter interacts with the allocrite. Although none of the tested compounds improved the antibody binding as strongly as established allocrites (vincristine for ABCB1 and mitoxantrone for ABCG2), (*S*,*Z*)-**4a**, (*S*,*Z*)-**4b**, (*S*,*Z*)-**4c**, and (*S*,*Z*)-**4d** exhibited binding patterns expected for ABCB1 allocrites (Figure 6). These four compounds were also recognized as ABCG2 allocrites, consistent with the results of the PheA accumulation assay.

**Figure 6:**
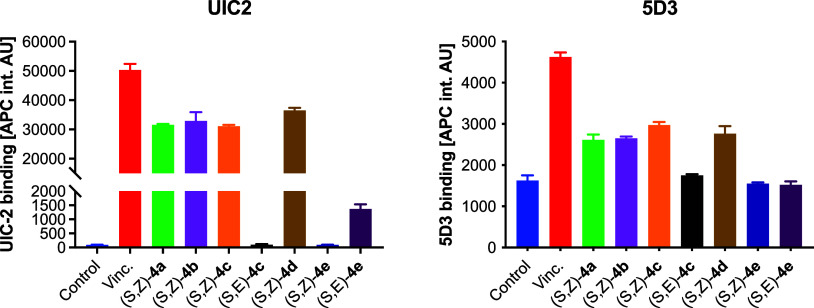
UIC-2 antibody binding to ABCB1 in SW620V cells (left) and 5D3 antibody binding to ABCG2 in SW620Mito cells (right) after exposure to 1 μM **4a**–**e** (*n* = 3).

An analysis of the biological results highlights that ferrocenyl-containing hybrids **4a**–**d** consistently outperformed the phenyl analogue **4e** in reversing multidrug resistance across various cell lines. This observation suggests a beneficial contribution of the ferrocene unit, potentially through enhanced lipophilicity and/or improved interactions with proteins. Tryprostatin A (TrpA) was used in the cytotoxicity assays to evaluate the intrinsic toxicity of the compounds against normal fibroblasts rather than as a reference for ABC transporter inhibition studies. The activity of the compounds against ABC transporters was assessed in comparison to established inhibitors such as MK571 (for ABCC1), Ko143 (for ABCG2), and verapamil (for ABCB1). Nevertheless, the low cytotoxicity of compounds **4a**–**d** compared with TrpA confirms that the introduction of the ferrocene moiety did not adversely affect cell viability, supporting the favorable safety profile of these ferrocenyl hybrids.

These observations provide a strong rationale for the further development of ferrocenyl derivatives as effective and safe modulators of multidrug resistance.

Furthermore, a direct correlation between the inhibition of ABC transporter activity and the observed ERP values was noted. The strong ERP values of (*S*,*Z*)-**4b**, (*S*,*Z*)-**4c**, and (*S*,*Z*)-**4d** in ABCB1-overexpressing SW620V cells correspond to their ability to inhibit calcein efflux and enhance UIC-2 antibody binding to ABCB1. Likewise, the high ERP value of (*S*,*Z*)-**4c** in ABCG2-overexpressing cells aligns with its ability to inhibit PheA efflux and to enhance the binding of the 5D3 antibody to ABCG2. Notably, (*S*,*Z*)-**4c** demonstrated markedly stronger ERP responses and cytotoxicity compared to (*S*,*E*)-**4c**, which showed only modest activity and no detectable interaction with either UIC2 or 5D3 antibodies. These findings support the involvement of ABC transporter modulation in the activity of (*S*,*Z*)-**4c**. Importantly, (*S*,*Z*)-**4c** also exhibited substantial intrinsic cytotoxicity toward both drug-sensitive SW620 and cisplatin-resistant SW620C cells, which was not observed for other compounds. However, given the exceptionally high ERP values—especially in combination with cisplatin—it is likely that additional mechanisms, including intrinsic cytotoxicity and potential synergistic effects, contribute to the overall biological activity. This highlights a multimodal mechanism of action rather than an exclusive reliance on transporter inhibition.

We synthesized and investigated a series of ferrocenyl–cyclo-(Gly-l-Pro) hybrids as potential multidrug-resistant protein inhibitors in contrast to their purely organic analog (**4e**). All of the investigated compounds exhibited low toxicity against both colon cancer cells and normal fibroblasts, suggesting a favorable safety profile. They could reverse ABCB1- and ABCG2-mediated drug resistance, sensitizing multidrug-resistant cells to conventional cytotoxic agents. Their effect was synergistic with anticancer agents, particularly in ABCG2-overexpressing cells. Real-time transport assays based on fluorescent substrates confirmed that the ferrocenyl hybrids inhibited the activity of both ABCB1 and ABCG2, and antibody-binding assays demonstrated their direct interactions with transporter protein molecules. These data emphasize the potential for ferrocenyl hybrids to act as chemosensitizers, restoring the effectiveness of standard chemotherapy against resistant tumors. Future studies should focus on the further optimization of these compounds to improve their potency and selectivity.

## Supplementary Material





## Notes

Detailed experimental section with description of chemical synthesis and biological experiments, supplementary figures, copies of the NMR spectra, and supplementary tables (PDF)
Molecular formula strings and associated biological data (XLSX)

## Notes

Deposition numbers 2207258–2207261 contain the supplementary crystallographic data for this paper. These data can be obtained free of charge via the Cambridge Crystallographic Data Center (CCDC).

## Abbreviations

ABC: ATP-binding cassette
ABCB1: ATP binding cassette subfamily B member 1
ABCC1: ATP binding cassette subfamily C member 1
ABCG2: ATP binding cassette subfamily G member 2
BCECF: 2′,7′-bis­(2-carboxyethyl)-5-(and-6)-carboxyfluorescein
cDDP: *cis*-diammine­di­chloro­platinum­(II)
DIC: *N*,*N′*-diisopropylcarbodiimide
DMF: *N*,*N*-dimethylformamide
ERP: enhancement of reversal potency
HOBt: 1-hydroxybenzotriazole
MCF-7: human breast cancer cell line
MDA-MB-231: triple-negative breast cancer cell line
MRC-5: human lung fibroblast cell line
MDR: multidrug resistance
MTX: methotrexate
ND: not detected
PheA: pheophorbide
SD: standard deviation
selROESY: selective rotating-frame Overhauser effect spectroscopy
SW620: human colorectal cancer cell line
SW620C: human colorectal cancer cell line cisplatin-resistant variant
SW620D: human colorectal cancer cell line doxorubicin-resistant variant
SW620E: human colorectal cancer cell line etoposide-resistant variant
SW620M: human colorectal cancer cell line methotrexate-resistant variant
SW620Mito: human colorectal cancer cell line mitoxantrone-resistant variant
SW620V: human colorectal cancer cell line vincristine-resistant variant
TrpA: tryprostatin A
UIC-2 antibody: anti-ABCB1 monoclonal antibody
VINC: vincristine
5D3 antibody: anti-ABCG2 monoclonal antibody
