# Supplementary material for: Design, Synthesis, and Biological Evaluation of Ferrocenyl–Cyclo-(Gly‑l‑Pro) Hybrids Sensitizing Multidrug-Resistant Cancer Cells to Anticancer Agents
Source: ACS Med Chem Lett. 2025 Jun 16;16(7):1391–400. doi: 10.1021/acsmedchemlett.5c00256 (PMC12257394; doi:10.1021/acsmedchemlett.5c00256)
Supplement: Supplementary file 1 [file ml5c00256_si_001.pdf]

Design, Synthesis, and Biological Evaluation of Ferrocenyl-Cyclo-(Gly-L-Pro) Hybrids Sensitizing Multidrug-Resistant Cancer Cells to Anticancer Agents

Andrzej Błauż[1], Karolina Rózga[2], Małgorzata Nosek[1], Anna Makal[3], Błażej Rychlik[1], Damian Plażuk*[4]

[1] Centre for Digital Biology and Biomedical Science - Biobank Lodz®, Faculty of Biology and Environmental Protection, University of Lodz, ul. Pomorska 141/143, 90-236 Łódź, Poland

[2] Department of Organic Chemistry, Faculty of Chemistry, University of Lodz, ul. Tamka 12, 91-403 Łódź, Poland

[3] Laboratory for Structural and Biochemical Research, Biological and Chemical Research Centre, Department of Chemistry, University of Warsaw, ul. Zwirki i Wigury 101, 02-089 Warszawa, Poland

[4] Laboratory of Molecular Spectroscopy, Department of Organic Chemistry, Faculty of Chemistry, University of Lodz, ul. Tamka 12, 91-403 Łódź, Poland

E-mail: damian.plazuk@chemia.uni.lodz.pl

Table of Contents

STEREOCHEMICAL COMPOSITION OF 4A-E. **3**

EXPERIMENTAL SECTION **4**

General Information 4
Synthesis 5
General procedure A: synthesis of 1,1-dibromo-2-(ferrocenylphenyl)ethenes 7b–d 5
General procedure B: synthesis of (ferrocenylphenyl)acetylenes 8b–d 7
General procedure C: Synthesis of (ferrocenylphenyl)propynoic acids 9a–d 9
General procedure D: Synthesis of 1-acyl-prolinamides 10a–e 11
General procedure E: synthesis of 1,4-piperazino-2,5-diones 4a–e 16
Crystallography 21

BIOLOGICAL ASSAYS **21**

Cell lines 21
Viability assay [12] 22
The enhancement of reversal potency (ERP) value determination 22
Transport assays 23
Antibody-binding assays 24
Synergy determination 24
Statistical analysis. 24
Additional figures 25

X-RAY DATA **35**

ADDITIONAL BIOLOGICAL DATA **40**

COPIES OF THE NMR SPECTRA **42**

REFERENCES **63**

Stereochemical composition of 4a-e.

All compounds except **4b** formed mixtures of major (*S,Z*)- and minor (*S,E*)-isomers, although in most cases, the (*Z*)-isomer could be isolated (Figures S1–S6). The (*E*)- and (*Z*)-configurations were determined by selROESY experiments (for examples see Figures S7–S15). Chiral HPLC analysis revealed that cyclizing **10** to **4** induced partial racemization, generating (*R,Z*)-isomers along with the predominant (*S,Z*)-isomers under both literature and microwave conditions (Figures S17-S21). For instance, reaction of (*S*)-**10a** yielded a mixture of (*S,Z*)-**4a** and (*R,Z*)-**4a**, with slightly reduced racemization under microwave conditions (Figure S16). Similarly, (*R,Z*)-**4b–e** were formed at 10%–30% relative abundance (Figures S17–S21). This partial racemization was further confirmed using (*S*)-**10e** and (*R*)-**10e**, each of which yielded mixtures of (*S,Z*)- and (*R,Z*)-**4e**. Chiral HPLC revealed two products with retention times t_1 = 14.1 min and t_2 = 22.9 min, corresponding to (*R,Z*)-**4e** and (*S,Z*)-**4e**, respectively (Figure S22). This behavior can be attributed to the presence of an α-proton in the proline moiety, which makes the stereocenter prone to base- or acid-catalyzed epimerization, leading to partial racemization during synthesis.

The structures of **4a**, **4d**, and **4e** were also confirmed by X-ray analysis (Figure S23, Table S1). Importantly, all the investigated compounds crystallized as mixture of diastereoisomers, with the fragment around chiral carbon C8a being disordered, which confirmed that racemization occurs during the reaction. The ratios of (*R,Z*) vs (*S,Z*) diastereoisomers in most cases were refined to 9:1 in a molecular site; in conjunction with crystallization in nonchiral space groups, this indicated a 1:1 (*R,Z*):(*S,Z*) isomer ratio in the crystal. Although (*S,E*)-**4e** also crystallized in a chiral $P2_1$ space group with no apparent disorder, the Flack parameter[1] in that case was indicative of racemic twinning and hence approximately 7:3 ratio of (*R,E*) vs (*S,E*) diastereoisomers. These findings confirmed that both isomers must have been present in

solution during the crystallization stage. The analyzed compounds exhibited overall typical bond lengths, the ligands were almost exactly coplanar with the substituted ferrocene ring, and the ferrocene moieties uniformly adopted an eclipsed conformation. Compound (*S,Z*)-**4e** exhibited two distinctive conformations in its two polymorphs, demonstrating flexibility of the phenyl-piperazine-2,5-dione linker; the phenyl ring was rotated away from the piperazino-2,5-dione (dihedral angle between these moieties being 178.9(4)°) in the $P2_1$ polymorph, whereas in the case of the $P4_2bc$ polymorph, the same dihedral angle was as small as 9.9(4)° (Figure S24). Detailed information regarding the data processing, structure solution, and refinement is shown in Table S1.

Experimental section

General Information

All reactions were performed under Ar atmosphere. Reagents and solvents were purchased from Avantor Performance Materials Poland (Gliwice, Poland) or Chempur (Piekary Śląskie, Poland) or Merck. ^{1}H, $^{13}C\{^{1}H\}$ and 2D NMR spectra were recorded on a Bruker 600 MHz Avance III spectrometer at 600.3 MHz for ^{1}H and 150.0 MHz for ^{13}C frequency or on Bruker 600 MHz Avance Neo spectrometer equipped with N2-cryoprobe with spectrometer frequencies of 600.1 MHz for ^{1}H and 150.0 MHz for ^{13}C and 300K. Chemical shifts were calibrated on residual solvents signals at δ = 2.51 ppm for ^{1}H and δ = 39.5 ppm for ^{13}C. Elemental analyses were performed at the Faculty of Chemistry, University of Lodz (Poland). Column chromatography was carried out with SilicaFlash® P60 (Silicycle, Canada) for flash chromatography (0.040–0.063 mm, 230–400 mesh). Reactions accelerated by microwave were performed in 10 mL or 30 mL reactions tube using Monowave 400 Microwave Reactor (Anton Paar) in temperature control mode. The typical reaction parameters are as follow: target temperature 120°C with an ASAP heating ramp, automatically adjusted power, stirring rate 600 min^{-1}, and pressure limit of 20 bar. MS analysis was performed on Shimadzu Nexera XR system

equipped with an SPD-M40 and an LCMS-2020 detector and Phenomenex XB-C18 column (50 × 4.6 mm, 2.1 mm, 1.7 μm, low rate 0.4 mL·min^{-1}). HPLC analysis was performed on Shimadzu HPLC system using Kinetex PFP column (150 × 4.0 mm, 5 μm, flow rate 1 mL·min^{-1}). Chiral HPLC analysis was performed using Lux Celluloxe-3 or Lux Cellulose-4 column (100 × 4.6 mm mm, 5 μm, flow rate 1 mL·min^{-1}) with a diode array detector (SPD-M20A). The composition of mobile phase is given for specific compound. Melting points (M.p.) were determined for all new compounds in capillaries with a DigiMelt MPA161 apparatus (SRS) and were uncorrected. IR spectra were performed on a FT-IR Nexus spectrometer in KBr pellets. Antibodies UIC-2 and 5D3 and respective isotype controls were purchased from Biolegend (San Diego, CA USA) and MBL Life Science (Tokyo, Japan). Buffers and aqueous solutions used to perform experiments were prepared using Milli-Q water (Milli-Q Integral water station, Millipore, Billerica, MA, USA). **5** was synthesized by refluxing of cyclo-(Gly-L-Pro)[2] with an excess of acetic anhydride for 1 h and used after the evaporation of volatiles without purification.

Synthesis

General procedure A: synthesis of 1,1-dibromo-2-(ferrocenylphenyl)ethenes 7b–d

First, 1.00 eq of an aldehyde **6b–d** ($C_{aldehyde}$ = 0.16 M) in DMC was added dropwise at RT to a solution containing 2.00 eq of carbon tetrabromide and 4.00 eq of triphenylphosphine in dichloromethane (DCM). After 15 min of stirring at RT, an equal amount of water was added, and the products were extracted with DCM. The organic solution was washed with brine, dried over sodium sulfate, evaporated, and purified on silica.

1,1-dibromo-2-(2-ferrocenylophenyl)ethene 7b

Compound **7b** was prepared in 81% yield (2.71 g) starting from 2.18 g (7.50 mmol) of 2-ferrocenylbenzaldehyde **6b** according to general procedure A. The product was isolated by column chromatography using DCM/cyclohexane 5/95 (v/v) as eluent. M.p. 62-63 °C. ^{1}H NMR (DMSO-d_6) δ 7.89 (s, 1H, H-2), 7.68 (d, J = 7.8 Hz, 1H, H-3′), 7.37 (d, J = 7.6 Hz, 1H, H-6′), 7.34 (t, J = 7.5 Hz, 1H, H-4′), 7.28 (t, J = 7.5 Hz, 1H, H-5′), 4.57-4.56 (m, 2H, Cp), 4.43-4.41 (m, 2H, Cp), 4.13 (s, 5H, Cp′). ^{13}C{^{1}H} NMR (DMSO-d_6) δ 138.3 (C-2), 136.8 (C-1′), 133.1 (C-2′), 129.6 (C-3′), 129.0 (C-6′), 128.3 (C-4′), 125.9 (C-5′), 90.8 (C-1), 84.4 (Cp_{ipso}), 69.3 (Cp′), 68.9 (Cp), 68.8 (Cp). IR (KBr, ν, cm^{-1}) 3025, 1104, 1000, 850, 822, 782, 754, 732, 606, 564, 491. ESI-MS (m/z) calc. for $C_{18}H_{14}{}^{79}Br_2Fe$ 443.9 $[M]^+$, found 443.8 $[M]^+$, calc. for $C_{18}H_{14}{}^{81}Br_2Fe$ 447.9 $[M]^+$, found 447.7 $[M]^+$, calc. for $C_{18}H_{14}{}^{79}Br^{81}BrFe$ 445.9 $[M]^+$, found 445.7 $[M]^+$. Elemental analysis calc. for $C_{18}H_{14}Br_2Fe$ C 48.48, H 3.16, found C 48.54, H 3.23%.

1,1-dibromo-2-(3-ferrocenylophenyl)ethene 7c

Compound **7c** was prepared in 88% yield (2.53 g) starting from 1.88 g (6.49 mmol) of 3-ferrocenylbenzaldehyde **6c** according to general procedure A. The product was isolated by column chromatography using DCM/cyclohexane 30/70 (v/v) as eluent. M.p. 107-108°C. ^{1}H NMR (DMSO-d_6) δ 7.81 (s, 1H, H-2), 7.77 (br s, 1H, H-2′), 7.54 (dt, J = 7.7, 1.3 Hz, 1H, H-4′), 7.44-7.42 (m, 1H, H-6′), 7.34 (t, J = 7.7 Hz, 1H, H-5′), 4.79 (t, J = 1.8 Hz, 2H, Cp), 4.37 (t, J = 1.8 Hz, 2H, Cp), 4.04 (s, 5H, Cp′). ^{13}C{^{1}H} NMR (DMSO-d_6) δ 139.4 (C-3′), 137.1 (C-2), 135.0 (C-1′), 128.5 (C-5′), 126.1 (C-4′), 125.8 (C-2′), 125.6 (C-6′), 89.2 (C-1), 84.1 (Cp_{ipso}), 69.4 (Cp′), 69.0 (Cp), 66.4 (Cp). IR (KBr, ν, cm^{-1}) 3016, 1599, 1104, 899, 855, 816, 801, 786, 693, 672, 601, 482. ESI-MS (m/z) calc. for $C_{18}H_{14}{}^{79}Br_2Fe$ 443.9 $[M]^+$, found 443.8 $[M]^+$, calc. for $C_{18}H_{14}{}^{81}Br_2Fe$ 447.9 $[M]^+$, found 447.6 $[M]^+$, calc. for $C_{18}H_{14}{}^{79}Br^{81}BrFe$ 445.9 $[M]^+$, found 445.7 $[M]^+$. Elemental analysis calc. for $C_{18}H_{14}Br_2Fe$ C 48.48, H 3.16, found C 48.32, H 3.44%.

1,1-dibromo-2-(4-ferrocenylphenyl)ethene 7d

Compound **7d** was prepared in 99% yield (1.22 g) starting from 0.80 g (2.76 mmol) of 4-ferrocenylbenzaldehyde **7d** according to general procedure A. The product was isolated by column chromatography using DCM/cyclohexane 5/95 (v/v) as eluent. M.p. 118-119°C. ^{1}H NMR (DMSO-d_6) δ 7.74 (s, 1H, H-2), 7.58 (s, 4H, H_{Ar}), 4.84 (t, J = 1.7 Hz, 2H, Cp), 4.39 (t, J = 1.7 Hz, 2H, Cp), 4.03 (s, 5H, Cp′). ^{13}C{^{1}H} NMR (DMSO-d_6) δ 140.0 (C-4′), 136.8 (C-2), 132.2 (C-1′), 128.4 (C-2′/6′), 125.7 (C-3′/C5′), 87.7 (C-1), 83.7 (Cp_{ipso}), 69.4 (Cp′), 69.3 (Cp), 66.4 (Cp). IR (KBr, ν, cm^{-1}) 3009, 2923, 2852, 1604, 1522, 1419, 1270, 1106, 1084, 1032, 996, 877, 859, 818, 775, 639, 629, 581, 528, 493. ESI-MS (*m/z*) calc. for $C_{18}H_{14}{}^{79}Br_2Fe$ 443.9 [M]$^+$, found 443.8 [M]$^+$, calc. for $C_{18}H_{14}{}^{81}Br_2Fe$ 447.9 [M]$^+$, found 447.6 [M]$^+$, calc. for $C_{18}H_{14}{}^{79}Br^{81}BrFe$ 445.9 [M]$^+$, found 445.7 [M]$^+$. Elemental analysis calc. for $C_{18}H_{14}Br_2Fe$ C 48.48, H 3.16, found C 48.30, H 3.26%.

General procedure B: synthesis of (ferrocenylphenyl)acetylenes 8b–d

First, 3.30 eq of n-BuLi (C = 2.5 M) was added to a −78°C cooled solution of 1.00 eq of 1,1-dibromo-2-(ferrocenylphenyl)ethene **7b–d** (C = 0.2 M) in anhydrous THF, and the resulting mixture was stirred at −78°C for an additional 1 h. Then, the reaction was quenched by water, diluted with two volumes of DCM, and neutralized with 1 M HCl, and the products were extracted with DCM. The organic solution was washed with brine, dried over sodium sulfate, and evaporated. The desired products were isolated by column chromatography on silica.

(2-ferrocenylphenyl)acetylene 8b

Compound **8b** was prepared in 75% yield (1.33 g) starting from 2.71 g (6.08 mmol) of **7b** according to general procedure B. The product was isolated by column chromatography using DCM/cyclohexane 5/95 (v/v) as eluent. M.p. 75-76°C. ^{1}H NMR (DMSO-d_6) δ 7.69 (dd, J = 7.9

, 1.0 Hz, 1H, H-3′), 7.43 (dd, J = 7.7, 1.3 Hz, 1H, H-6′), 7.35 (td, J = 7.6, 1.3 Hz, 1H, H-5′), 7.20 (td, J = 7.5, 1.2 Hz, 1H, H-4′), 4.96 (t, J = 1.9 Hz, 2H, Cp), 4.39 (s, 1H, H-1), 4.37 (t, J = 1.8 Hz, 2H, Cp), 4.09 (s, 5H, Cp′). $^{13}C\{^{1}H\}$ NMR (DMSO-d_6) δ 140.7 (C-2′), 134.0 (C-6′), 129.2 (C-3′), 128.7 (C-5′), 125.8 (C-4′), 118.9 (C-1′), 84.5 (C-1), 84.0 (C-2), 83.9 (Cp_{ipso}), 69.5 (Cp′), 68.7 (Cp), 68.5 (Cp). IR (KBr, ν, cm^{-1}) 3312, 3259, 2101, 1496, 825, 770, 762, 676, 627, 598, 497, 488, 473. ESI-MS (*m/z*) calc. for $C_{18}H_{14}Fe$ 286.0 $[M]^{+}$, found 286.0 $[M]^{+}$. Elemental analysis calc. for $C_{18}H_{14}Fe$ C 75.55, H 4.93, found C 75.50, H 4.98%.

(3-ferrocenylphenyl)acetylene 8c

Compound **8c** was prepared in 72% yield (1.17 g) starting from 2.53 g (5.67 mmol) of **7c** according to general procedure B. The product was isolated by column chromatography using DCM/cyclohexane 5/95 (v/v) as eluent. M.p. 114-115°C. ^{1}H NMR (DMSO-d_6) δ 7.62 (br s, 1H, H-4′), 7.60-7.58 (m, 1H, H-6′), 7.33-7.30 (m, 2H, H-2′ and H-5′), 4.84 (t, J = 1.7 Hz, 2H, Cp), 4.37 (t, J = 1.7 Hz, 2H, Cp), 4.21 (s, 1H, H-1), 4.02 (s, 5H, Cp′). $^{13}C\{^{1}H\}$ NMR (DMSO-d_6) δ 139.8 (C-3′), 129.0 (C-5′), 128.7 (C-2′), 128.5 (C-4′), 126.5 (C-6′), 121.8 (C-1′), 83.7 (C-2), 83.4 (Cp_{ipso}), 80.6 (C-1), 69.4 (Cp′), 69.2 (Cp), 66.4 (Cp). IR (KBr, ν, cm^{-1}) 3280, 3108, 3096, 3082, 2109, 1593, 1497, 1456, 1407, 1288, 1104, 1030, 998, 891, 814, 795, 689, 672, 661, 621, 504, 487. ESI-MS (*m/z*) calc. for $C_{18}H_{14}Fe$ 286.0 $[M]^{+}$, found 285.9 $[M]^{+}$. Elemental analysis calc. for $C_{18}H_{14}Fe$ C 75.55, H 4.93, Found C 75.51, H 4.85%.

(4-ferrocenylphenyl)acetylene 8d

Compound **8d** was prepared in 65% yield (0.534 g) starting from 1.26 g (2.84 mmol) of **7d** according to general procedure B. The product was isolated by column chromatography using a gradient of DCM in cyclohexane starting from 5% to 100% of DCM (v/v) as eluent. M.p. 104-105°C. ^{1}H NMR (DMSO-d_6) δ 7.55 (d, J = 8.3 Hz, 2H, H-3′/5′), 7.40 (d, J = 8.3 Hz, 2H,

H-2′/6′), 4.83 (t, J = 1.8 Hz, 2H, Cp), 4.39 (t, J = 1.8 Hz, 2H, Cp), 4.19 (s, 1H, H-1), 4.02 (s, 5H, Cp′). $^{13}C\{^1H\}$ NMR (DMSO-d_6) δ 140.3 (C-1′), 131.7 (C-2′/6′), 125.8 (C-3′/5′), 118.7 (C-4′), 83.9 (C-2), 83.4 (Cp_{ipso}), 80.6 (C-1), 69.5 (Cp′), 69.4 (Cp), 66,4 (Cp). IR (KBr, ν, cm^{-1}) 3286, 2104, 1664, 1645, 1604, 1106, 835, 820, 659, 614, 549, 487. ESI-MS (*m*/*z*) calc. for $C_{18}H_{14}Fe$ 286.0 $[M]^+$, found 285.9 $[M]^+$. Elemental analysis calc. for $C_{18}H_{14}Fe$ C 75.55, H 4.93, found C 75.51, H 4.87%.

General procedure C: Synthesis of (ferrocenylphenyl)propynoic acids 9a–d

First, 1.20 eq of n-BuLi (C = 1.6 M) was added to a −78°C cooled solution of 1.00 eq acetylene **8a–d** (C = 0.2 M) in THF. The resulting solution was stirred at −78°C for 1 h, and then an excess of crushed dry ice was added in small portions within 1 h. Next, the reaction mixture was left to reach RT, and two volumes of DCM was added followed by an excess of saturated solution of citric acid, after which the product was extracted with DCM. The organic solution was washed with brine, dried over sodium sulfate, and evaporated to dryness. Pure products were isolated by column chromatography on silica and used in the next step without further analysis.

Ferrocenylpropynoic acid 9a

Compound **9a** was prepared in 77% yield (0.463 g) starting from 0.500 g (2.38 mmol) of **8a** according to general procedure C. The product was isolated by column chromatography using a gradient of MeOH in DCM starting from 1% to 30% of MeOH (v/v) as eluent. The NMR spectra correspond to the reported data.[3]

(2-ferrocenylphenyl)propynoic acid 9b

Compound **9b** was prepared in 46% yield (0.665 g) starting from 1.25 g (4.37 mmol) of **8b** according to general procedure C. The product was isolated by column chromatography using

a gradient of MeOH in DCM starting from 1% to 10% of MeOH (v/v) as eluent. M.p. >165°C (dec.). ^{1}H NMR (DMSO-d_6) δ 13.82 (br. s, 1H, COOH), 7.72 (d, J = 7.9 Hz, 1H, H-3′), 7.55 (d, J = 7.7 Hz, 1H, H-6′), 7.45 (td, J = 7.7, 1.1 Hz, 1H, H-4′), 7.28 (td, J = 7.6, 1.0 Hz, 1H, H-5′), 4.96 (t, J = 1.7 Hz, 2H, Cp), 4.43 (t, J = 1.7 Hz, 2H, Cp), 4.12 (s, 5H, Cp′). $^{13}C\{^{1}H\}$ NMR (DMSO-d_6) δ 154.7 (C-1), 142.6 (C-1′), 134.8 (C-6′), 130.7 (C-4′), 129.2 (C-3′), 126.1 (C-5′), 116.0 (C-2′), 85.5 (C-2), 85.3 (C-3), 83.0 (Cp_{ipso}), 69.6 (Cp′), 69.1 (Cp), 68.5 (Cp). IR (KBr, ν, cm^{-1}) 2201 (C≡C), 1663 (C=O). ESI-MS (m/z) calc. for $C_{19}H_{14}FeO_2$ 330.0 $[M]^+$, found 329.9 $[M]^+$. Elemental analysis calc. for $C_{19}H_{14}FeO_2$ C 69.12, H 4.27, found C 69.15, H 4.39%.

(3-ferrocenylphenyl)propynoic acid 9c

Compound **9c** was prepared in 54% yield (0.727 g) starting from 1.17 g (4.08 mmol) of **8c** according to general procedure C. The product was isolated by column chromatography using a gradient of MeOH in DCM starting from 1% to 5% of MeOH (v/v) as eluent. M.p. >140°C (dec.). ^{1}H NMR (DMSO-d_6) δ 13.82 (br s, 1H, COOH), 7.77 (br s, 1H, H-2′), 7.72 (d, J = 7.8 Hz, 1H, H-4′), 7.44 (d, J = 7.7 Hz, 1H, H-6′), 7.40 (t, J = 7.7 Hz, 1H, H-5′), 4.90 (t, J = 1.8 Hz, 2H, Cp), 4.39 (t, J = 1.7 Hz, 2H, Cp), 4.03 (s, 5H, Cp′). $^{13}C\{^{1}H\}$ NMR (DMSO-d_6) δ 154.4 (C-1), 140.4 (C), 129.8 (C-6′), 129.2 (C-5′), 129.1 (C-2′), 128.2 (C-4′), 119.1 (C-1′), 84.7 (C-3), 82.9 (Cp_{ipso}), 81.6 (C-2), 69.5 (Cp′), 69.4 (Cp), 66.5 (Cp). IR (KBr, ν, cm^{-1}) 2215 (C≡C), 1683 (C=O). ESI-MS (m/z) calc. for $C_{19}H_{14}FeO_2$ 330.0 $[M]^+$, found: 329.9 $[M]^+$. Elemental analysis calc. for $C_{19}H_{14}FeO_2$ C 69.12, H 4.27, found C 68.98, H 4.24%.

(4-ferrocenylphenyl)propynoic acid 9d

Compound **9d** was prepared in 82% yield (1.37 g) starting from 1.46 g (5.10 mmol) of **8d** according to general procedure C. The product was isolated by column chromatography using a gradient of MeOH in DCM starting from 1% to 10% of MeOH (v/v) as eluent. M.p. 156-

157°C. ^{1}H NMR (DMSO-d_6) δ 13.73 (br. s, 1H, COOH), 7.61 (d, J = 8.3 Hz, 2H, H-3′/5′), 7.53 (d, J = 8.3 Hz, 2H, H-2′/6′), 4.87 (t, J = 1.6 Hz, 2H, Cp), 4.42 (t, J = 1.6 Hz, 2H, Cp), 4.02 (s, 5H, Cp′); ^{13}C{^{1}H} NMR (DMSO-d_6) δ 154.5 (C-1), 142.9 (C-1′), 132.8 (C-2′/6′), 126.1 (C-3′/5′), 115.6 (C-4′), 85.2 (C-3), 82.7 (Cp_{ipso}), 81.9 (C-2), 69.8 (Cp), 69.6 (Cp′), 66.7 (Cp). IR (KBr, ν, cm^{-1}) 2198 (C≡C), 1668 (C=O). ESI-MS (*m/z*) calc. for $C_{19}H_{14}FeO_2$ 330.0 [M]$^+$, found 329.8 [M]$^+$. Elemental analysis calc. for $C_{19}H_{14}FeO_2$ C 69.12, H 4.27, found C 68.92, H 4.41%.

General procedure D: Synthesis of 1-acyl-prolinamides 10a–e

First, 1.10 eq od N,N′-diisopropylcarbodiimide was added to a stirred solution of 1.00 eq of propynoic acid **9a–e** (C = 0.2 M), 1.00 eq of L-prolinamide, and 1.00 eq of 1-hydroxybenzotriazole in DMF, and the resulting solution was stirred for 18 h at RT. Next, two volumes of ethyl acetate followed by an equal volume of 1 M HCl were added, and the products were isolated with ethyl acetate. The organic solution was washed with brine, dried over sodium sulfate, and evaporated to dryness. The desired products were isolated by column chromatography on silica.

(*S*)-1-(3-ferrocenylpropynoyl)prolinamide 10a

Compound **10a** was prepared in 70% yield (133 mg) starting from 143 mg (0.563 mmol) of **9a** according to general procedure D. The product was isolated by column chromatography using a gradient of MeOH in DCM starting from 1% to 3% of MeOH (v/v) as eluent with a mixture of two rotamers, major A and minor B. M.p. > 210°C (dec.). ^{1}H NMR (DMSO-d_6) δ 7.62 (br s, 0.6 H, NH, rotamer A), 7.41 (br s, 0.4 H, NH, rotamer B), 7.18 (br s, 0.6 H, NH, rotamer A), 6.97 (br s, 0.4 H, NH, rotamer B), 4.65 (br s, 0.8 H, Cp, rotamer A and B), 4.62-4.60 (m, 1.2 H, Cp, rotamer A and B), 4.46 (dd, J = 8.6, 2.9 Hz, 0.7 H, H-2, rotamer A), 4.42-4.39 (m, 2H, Cp, rotamer A and B), 4.29 (s, 2H, Cp′, rotamer B), 4.28 (s, 2.8 H, Cp′, rotamer A), 4.21 (dd, J = 8.6, 3.6 Hz, 0.4 H, H-2, rotamer B), 3.72-3.68 (m, 0.9 H, H-5, rotamer B), 3.47-3.38 (m, 1.4

H, H-5, rotamer A), 2.30-2.23 (m, 0.7 H, H-3, rotamer A), 2.17-2.11 (m, 0.4 H, H-3, rotamer B), 1.94-1.87 (m, 1.5 H, H-4, rotamer A and B), 1.87-1.81 (m, 1.9 H, H-4, rotamer A and B); $^{13}C\{^1H\}$ NMR (DMSO-d_6) δ 173.5 (C=O, rotamer A), 172.9 (C=O, rotamer B), 152.2 (C=O, C-1′, rotamer A), 151.8 (C=O, C-1′, rotamer B), 89.3 (C-2′/3′, rotamer B), 89.1 (C-2′/3′, rotamer A), 79.9 (Cp_{ipso}), 72.2 (Cp, rotamer A), 72.0 (Cp, rotamer B), 71.9 (Cp, rotamer B), 70.2 (Cp′, rotamer A), 70.0 (Cp′, rotamer B), 69.95 (Cp, rotamer B), 69.93 (Cp, rotamer B), 61.1 (C-2, rotamer A), 58.9 (C-2, rotamer B), 48.3 (C-5, rotamer B), 45.9 (C-5, rotamer A), 31.1 (C-3, rotamer A), 29.9 (C-3, rotamer B), 23.7 (C-4, rotamer B), 22.7 (C-4, rotamer A). IR (KBr, ν, cm^{-1}) 3327, 2928, 2850, 1626, 1575. ESI-MS (*m/z*) calc. for $C_{18}H_{18}FeN_2O_2$ 350.1 $[M]^+$, found 350.8 $[M]^+$. Elemental analysis – despite repeated attempts, including extended drying of the sample, satisfactory elemental analysis results could not be obtained for this compound – calc. for $C_{18}H_{18}FeN_2O_2$ C 61.74, H 5.18, N 8.00. found C 58.53, H 7.97, N 9.73%.

(*S*)-1-[3-(2-ferrocenylphenyl)propynoyl]prolinamide 10b

Compound **10b** was prepared in 94% yield (365 mg) starting from 300 mg (0.909 mmol) of **9b** according to general procedure D. The product was isolated by column chromatography using a gradient of MeOH in DCM starting from 1% to 2% of MeOH (v/v) as eluent with M.p. > 210°C (dec.) and a mixture of rotamers, major A and minor B. M.p. 109-110°C. 1:1 mixture of rotamers. ^1H NMR (DMSO-d_6) δ 7.75 (d, J = 7.5 Hz, 0.5 H, CH_{Ar}), 7.56 (d, J = 7.6 Hz, 0.5 H, CH_{Ar}), 7.63 (br s, 0.5 H, NH), 7.57-7.54 (m, 1H, CH_{Ar}), 7.46-7.40 (m, 1.5 H, CH_{Ar}), 7.30-7.23 (m, 1H, CH_{Ar}), 7.22 (br s, 0.5 H, NH), 7.00 (br s, 0.5 H, NH), 4.96-4.94 (m, 2H, Cp), 4.58 (dd, J = 8.6, 3.2 Hz, 0.5 H, H-2), 4.41 (br. s, 2H, Cp), 4.27 (dd, J = 8.6, 3.8 Hz, 0.5 H, H-2), 4.14 (s, 2.5H, Cp′), 4.13 (s, 2.5H, Cp′), 3.75-3.72 (m, 1H, H-5), 3.54-3.50 (m, 0.5 H, H-5), 3.49-3.45 (m, 0.5 H, H-5), 2.30-2.24 (m, 0.5 H, H-3), 2.19-2.13 (m, 0.5 H, H-3), 1.97-1.91 (m, 1H, H-3), 1.89-1.83 (m, 2H, H-4). $^{13}C\{^1H\}$ NMR (DMSO-d_6), signals from both rotamers are presented,

δ 173.5 (CONH$_2$), 172.8 (CONH$_2$), 152.3 (C≡$\underline{\text{C}}$CO), 151.8 (C≡$\underline{\text{C}}$CO), 142.05 (C$_{Ar}$), 142.00 (C$_{Ar}$), 134.8 (CH$_{Ar}$), 134.5 (CH$_{Ar}$), 130.2 (CH$_{Ar}$), 130.2 (CH$_{Ar}$), 129.4 (CH$_{Ar}$), 129.0 (CH$_{Ar}$), 126.1 (CH$_{Ar}$), 125.8 (CH$_{Ar}$), 117.0 (C$_{Ar}$), 116.9 (C$_{Ar}$), 88.7 ($\underline{\text{C}}$≡CCO), 88.2 ($\underline{\text{C}}$≡CCO), 86.5 (C≡$\underline{\text{C}}$CO), 86.4 (C≡$\underline{\text{C}}$CO), 83.5 (Cp$_{ipso}$), 83.2 (Cp$_{ipso}$), 69.67 (Cp′), 69.65 (Cp′), 69.1 (Cp), 69.0 (Cp), 68.9 (Cp), 68.8 (Cp), 68.7 (Cp), 68.5 (Cp), 68.4 (Cp), 61.1 (C-2), 59.0 (C-2), 48.3 (C-5), 46.1 (C-5), 31.0 (C-3), 30.0 (C-3), 23.7 (C-4), 22.8 (C-4). IR (KBr, ν, cm^{-1}) 2204, 1686, 1617, 1459, 1409. MS (ESI, *m*/*z*) calc. for $C_{24}H_{22}FeN_2O_2$ 426.1 [M]$^+$, found 426.0 [M]$^+$. Elemental analysis calc. for $C_{24}H_{22}FeN_2O_2$ C 67.62, H 5.20, N 6.57, found C 67.40, H 5.50, N 6.40.

(*S*)-1-[3-(3-ferrocenylphenyl)propynoyl]prolinamide 10c

Compound **10c** was prepared in 91% yield (304 mg) starting from 260 mg (0.788 mmol) of **9c** according to general procedure D. The product was isolated by column chromatography using a gradient of MeOH in DCM starting from 1% to 5% of MeOH (v/v) as eluent with a mixture of rotamers, major A and minor B. M.p. 101-102°C. ^{1}H NMR (DMSO-*d*$_6$) δ 7.75 (s, 0.4 H, CH$_{Ar}$), 7.70-7.68 (m, 2.3 H, CH$_{Ar}$, rotamer A and B), 7.45-7.36 (m, 2.5 H, CH$_{Ar}$, rotamer A and B), 7.26 (br s, 0.6 H, NH, rotamer A), 7.00 (br s, 0.4 H, NH, rotamer B), 4.89 (t, *J* = 1.7 Hz, 0.8 H, Cp), 4.88-4.87 (m, 1.2 H, Cp), 4.59 (dd, *J* = 8.6, 3.3 Hz, 0.6 H, H-2, rotamer A), 4.39 (t, *J* = 1.8 Hz, 2H, Cp), 4.27 (dd, *J* = 8.5, 3.8 Hz, 0.4 H, H-3, rotamer B), 4.02 (s, 2.8 H, Cp′), 4.03 (s, 2.1 H, Cp′), 3.83-3.78 (m, 0.9 H, H-5, rotamer B), 3.53-3.49 (m, 0.6 H, H-5, rotamer A), 3.48-3.43 (m, 0.6 H, H-5, rotamer A), 2.32-2.26 (m, 0.6 H, H-3, rotamer A), 2.19-2.15 (m, 0.4 H, H-3, rotamer B), 1.97-1.91 (m, 1.2 H, H-3 rotamer A, H-4, rotamer B), 1.88-1.84 (m, 2.1 H, H-4, rotamer A i B). ^{13}C{^{1}H} NMR (DMSO-*d*$_6$) δ 173.7 (CONH$_2$, rotamer A), 172.8 (CONH$_2$, rotamer B), 152.9 (C≡$\underline{\text{C}}$O, rotamer A), 151.5 (C≡$\underline{\text{C}}$O, rotamer B), 140.2 (C$_{Ar}$, rotamer B), 140.1 (C$_{Ar}$, rotamer A), 129.7 (CH$_{Ar}$, rotamer A), 129.5 (CH$_{Ar}$, rotamer B), 129.2 (CH$_{Ar}$, rotamer A), 129.0 (CH$_{Ar}$, rotamer B), 128.9 (CH$_{Ar}$, rotamer A), 127.9 (CH$_{Ar}$, rotamer B), 127.9

(CH_{Ar}), 119.8 (C_{Ar}), 88.1 (C≡C, rotamer B), 87.5 (C≡C, rotamer A), 83.0 (Cp_{ipso}, rotamer B), 83.0 (Cp_{ipso}, rotamer A), 82.7 (C≡C, rotamer B), 82.6 (C≡C, rotamer A), 69.47 (Cp′, rotamer A), 69.46 (Cp′, rotamer B), 69.32 (Cp), 69.30 (Cp), 66.48 (Cp), 66.45 (Cp), 66.43 (Cp), 61.2 (C-2, rotamer A), 59.0 (C-2, rotamer B), 48.5 (C-5, rotamer B), 46.1 (C-5, rotamer A), 31.0 (C-3, rotamer A), 30.0 (C-3, rotamer B), 23.7 (C-4, rotamer B), 22.8 (C-4, rotamer A). IR (KBr, ν, cm^{-1}) 3320, 3185, 3090, 2953, 2922, 2874, 2211, 1683, 1617, 1497, 1461, 1409, 1105, 796, 731, 689, 501, 487. ESI-MS (*m/z*) calc. for $C_{24}H_{22}FeN_2O_2$ 426.1 $[M]^+$, found 426.0 $[M]^+$. Elemental analysis calc. for $C_{24}H_{22}FeN_2O_2$ C 67.62, H 5.20, N 6.57, found C 67.46, H 5.48, N 6.67%.

(*S*)-1-[3-(4-ferrocenylphenyl)propynoyl]prolinamide 10d

Compound **10d** was prepared in 72% yield (149 mg) starting from 161 mg (0.909 mmol) of **9d** according to general procedure D. The product was isolated by column chromatography using a gradient of MeOH in DCM starting from 1% to 2% of MeOH (v/v) as eluent with a mixture of rotamers, major A and minor B. M.p. 99-100 °C. ^{1}H NMR (DMSO-d_6) δ 7.67 (br s, 0.6 H, NH, rotamer A), 7.62-7.59 (m, 2.1 H, H-3′/5′), 7,53 (d, J = 8.3 Hz, 0.9 H, H-2′/6′, rotamer B), 7.50 (d, J = 8.3 Hz, 1.2 H, H-2′/6′, rotamer A), 7.44 (br s, 0.4 H, NH, rotamer B), 7.22 (br s, 0.6 H, NH, rotamer A), 7.00 (br s, 0.4 H, NH, rotamer B), 4.89-4.88 (m, 2.1 H, Cp), 4.56 (dd, J = 8.6, 3.3 Hz, 0.6 H, H-2, rotamer A), 4.43-4.42 (m, 2.1 H, Cp), 4.26 (dd, J = 8.6, 3.7 Hz, 0.4 H, H-2, rotamer B), 4.03 (s, 2.2 H, Cp′, rotamer B), 4.02 (m, 2.8 H, Cp′, rotamer A), 3.81-3.74 (m, 0.8 H, H-6, rotamer B), 3.52-3.42 (m, 1.2 H, H-6, rotamer A), 2.30-2.24 (m, 0.6 H, H-3, rotamer A), 2.20-2.13 (m, 0.5 H, H-3, rotamer B), 1.97-1.91 (m, 1.2 H, H-3 rotamer A, H-4 rotamer B), 1.88-1.83 (m, 2.1 H, H-4, rotamer A and B). ^{13}C{^{1}H} NMR (DMSO-d_6) δ 173.6 ($CONH_2$, rotamer A), 172.8 ($CONH_2$, rotamer B), 152.1 (C≡C$\underline{C}$O, rotamer A), 151.7 (C≡CCO, rotamer B), 142.30 (C-1′, rotamer A), 142.26 (C-1′, rotamer B), 132.5 (C-2′/6′, rotamer A), 132.3 (C-

2′/6′, rotamer B), 126.0 (C-3′/5′, rotamer B), 125.9 (C-3′/5′, rotamer A), 116.46 (C-4′, rotamer A), 116.42 (C-4′, rotamer B), 88.6 (C≡C), 88.0 (C≡C), 83.0 (Cp_{ipso}), 82.93 (Cp_{ispo}), 82.91 (C≡C), 82.88 (C≡C), 69.7 (Cp′, rotamer B), 69.6 (Cp, rotamer A), 66.7 (Cp), 61.1 (C-2, rotamer A), 59.0 (C-2, rotamer B), 48.4 (C-5, rotamer B), 46.1 (C-5, rotamer A), 31.0 (C-3, rotamer A), 30.0 (C-3, rotamer B), 23.7 (C-4, rotamer B), 22.8 (C-4, rotamer A). IR (KBr, ν, cm^{-1}) 3307, 3185, 3088, 2923, 2875, 2205, 1693, 1617, 1601, 1524, 1409, 1105. ESI-MS (*m*/*z*) calc. for $C_{24}H_{22}FeN_2O_2$ 426.1 $[M]^+$, found 426.1 $[M]^+$. Elemental analysis calc. for $C_{24}H_{22}FeN_2O_2$ C 67.62, H 5.20, N 6.57, found C 67.57, H 5.38, N 6.39%.

(*S*)-1-(phenylpropynoyl)prolinamide 10e

Compound **10e** was prepared in 98% yield (817 mg) starting from 500 mg (3.43 mmol) of **10e** according to general procedure D. The product was isolated by column chromatography using a gradient of MeOH in DCM starting from 1% to 4% of MeOH (v/v) as eluent with a mixture of rotamers, major A and minor B. ^{1}H NMR (DMSO-d_6) δ 7.65-7.62 (m, 1.4 H, CH_{Ar}), 7.60-7.58 (m, 1.2 H, CH_{Ar}), 7.54-7.50 (m, 1H, CH_{Ar}), 7.48-7.44 (m, 2.4 H, CH_{Ar}), 7.19 (br s, 0.6 H, NH, rotamer A), 7.00 (br s, 0.4 H, NH, rotamer B), 4.54 (dd, *J* = 8.7, 3.4 Hz, 0.6 H, H-2, rotamer A), 4.26 (dd, *J* = 8.6, 3.8 Hz, 0.4 H, H-2, rotamer B), 3.79-3.73 (m, 0.9 H, H-5, rotamer B), 3.51-3.41 (m, 1.2 H, H-5, rotamer A), 2.30-2.23 (m, 0.6 H, H-3, rotamer A), 2.18-2.13 (m, 0.4 H, H-3, rotamer B), 1.95-1.90 (m, 1H, H-3 rotamer A, H-4 rotamer B), 1.89-1.82 (m, 2H, H-4, rotamer A and B). $^{13}C\{^1H\}$ NMR (DMSO-d_6) δ 173.5 ($CONH_2$, rotamer A), 172.8 ($CONH_2$, rotamer B), 151.9 (C≡C$\underline{C}$O, rotamer A), 151.5 (C≡C$\underline{C}$O, rotamer B), 132.4 (CH_{Ph}, rotamer A), 132.2 (CH_{Ph}, rotamer B), 130.5 (CH_{Ph}), 129.0 (CH_{Ph}, rotamer B), 128.9 (CH_{Ph}, rotamer A), 119.73 (C_{Ph}, rotamer A), 119.68 (C_{Ph}, rotamer B) 87.8 ($\underline{C}$≡CCO, rotamer B), 87.3 ($\underline{C}$≡CCO, rotamer A), 82.9 (C≡$\underline{C}$CO, rotamer B), 82.8 (C≡$\underline{C}$CO, rotamer A), 61.1 (C-2, rotamer A), 59.0 (C-2, rotamer B), 48.4 (C-5, rotamer B), 46.1 (C-5, rotamer A), 31.0 (C-3, rotamer A), 30.0 (C-

3, rotamer B), 23.7 (C-4, rotamer B), 22.8 (C-4, rotamer A); IR (KBr, ν, cm^{-1}) 3382, 3194, 2203, 1698, 1664, 1591, 1490, 1428, 1403, 1347, 1289, 761, 735, 695; ESI-MS (*m/z*) calc. for $C_{14}H_{15}N_2O_2$ $[M+H]^+$ 243.1, found: 243.0 $[M+H]^+$, Elemental analysis calc. for $C_{14}H_{14}N_2O_2$ C 69.41, H 5.82, N 11.56, found C 69.24, H 5.94, N 11.81%.

General procedure E: synthesis of 1,4-piperazino-2,5-diones 4a–e

A solution of 1.00 eq of amide **9a–e** (C = 0.09) and 1.00 eq of L-glutamic acid in a mixture of toluene-DMF (6:1, v:v) was added to a reaction flask. Next, 0.20–0.30 eq of trimethylphosphine (as 1 M solution in THF) was added to the obtained solution. Then, the reaction flask was closed with a PTFE cap and placed in a microwave reactor. The reaction was performed at 120°C until completion. Next, the volatiles were evaporated, and the products were isolated by chromatography on silica.

(*S,Z*)- and (*S,E*)-3-(ferrocenylmethylidene)hexahydropyrrolo[1,2-a]pyrazin-1,4-dione 4a

Compound **4a** was prepared in a total 77.5% yield (107 mg) starting from 138 mg (0.395 mmol) of **10a** according to general procedure E. The total reaction time was 60 min (2 × 30 min). The isomers (*S,Z*)- and (*R*,Z)-**4a** (74 mg, 53.6%, chiral HPLC analysis confirmed partial racemization, indicating a mixture of (*S,Z*):(*R,Z*) isomers in a 78:22 ratio) followed by (*S,E*)- and (*R,E*)-**4a** (33 mg, 23.9%) were isolated by column chromatography using a gradient of MeOH in DCM starting from 1% to 2% of MeOH (v/v) as eluent. Mixture of 78% of (*S,Z*)-**4a** and 22% of (*R,Z*)-**4a**. M.p. >220°C (dec). ^{1}H NMR (DMSO-d_6) δ 9.28 (s, 1H, H-2), 6.48 (s, 1H, CH=), 4.65 (br s, 2H, Cp), 4.42-4.41 (m, 1H, Cp), 4.39-4.38 (m, 1H, Cp), 4.34 (dd, *J* = 9.4, 6.5 Hz, 1H, H-8a), 4.17 (s, 5H, Cp′), 3.55-3.50 (m, 1H, H-6), 3.47-3.43 (m, 1H, H-6), 2.24-2.20 (m, 1H, H-8), 1.96-1.83 (m, 3H, H-7 and 8). ^{13}C{^{1}H} NMR (DMSO-d_6) δ 166.5 (C-1), 158.5 (C-4), 125.9 (C-3), 114.7 (CH=), 77.0 (Cp_{ipso}), 69.9 (Cp), 69.6 (Cp), 69.6 (Cp), 69.2 (Cp), 69.2

(Cp′), 58.2 (C-8a), 45.0 (C-6), 28.0 (C-8), 21.6 (C-7). IR (KBr, ν, cm^{-1}) 3085, 2928, 2883, 1686, 1628, 1441, 1382. ESI-MS (*m*/*z*) calc. for $C_{18}H_{18}FeN_2O_2$ 350.1 $[M]^+$, found 350.0 $[M]^+$. Elemental analysis calc. for $C_{18}H_{18}FeN_2O_2$ C 61.74, H 5.18, N 8.00 found C 61.57, H 5.25, N 8.09 %. R_f (HPLC) τ = 12.99 min; R_f (HPLC-chiral) τ_1 = 7.77 min (78%, (*S,Z*)-**4a**) τ_2 = 8.99 min (22%, (*R,Z*)-**4a**) (column Lux Cellulose-4; eluent: MeCN–water (9:1, v/v)). Mixture of (*S,E*)- and (*R,E*)-**4a** (these isomers were isolated as a mixture with (*S,Z*)- and (*R,Z*)-**4a)**. ^{1}H NMR (DMSO-d_6) δ 10.13 (br s, 1H, NH); 6.10 (s, 1H, CH=); 4.83 (br s, 1H, Cp); 4.64 (br s, 1H, Cp); 4.29-4.27 (m, 2H, Cp); 4.25–4.23 (m, 1H, H-8a); 4.13 (s, 5H, Cp′); 3.53-3.48 (m, 1H, H-6); 3.41-3.38 (m, 1H, H-6); 2.19-2.16 (m, 1H, H-8); 1.90-1.81 (m, 3H, H-7 and 8). ESI-MS (*m*/*z*) calc. for $C_{18}H_{18}FeN_2O_2$ 350.1 $[M]^+$, found: 350.0 $[M]^+$. R_f (HPLC) τ = 12.51 min.

(*S,Z*)-3-(2-ferrocenylbenzylidene)heksahydropyrrolo[1,2-a]pyrazin-1,4-dione 4b

Compound **4b** was prepared in 29.5% yield (82 mg) starting from 278 mg (0.652 mmol) of **10b** according to general procedure E. The total reaction time was 150 min (5 × 30 min). The product was isolated by column chromatography as a mixture of (*S,Z*)- and (*R,Z*)-isomers (chiral HPLC analysis confirmed partial racemization, indicating a mixture of (*S,Z*):(*R,Z*) isomers in a 90:10 ratio) using a gradient of MeOH in DCM starting from 1% to 3% of MeOH (v/v) as eluent. Mixture of 90% of (*S,Z*)-**4b** and 10% of (*R,Z*)-**4b** M.p. >135°C (dec). ^{1}H NMR (DMSO-*d₆*) δ 9.93 (s, 1H, NH), 7.63-7.62 (s, 1H, H-3′), 7.50-7.49 (m, 1H, H-6′), 7.28-7.24 (m, 3H, H-4′, 5′ and CH=), 4.63-4.62 (m, 1H, Cp), 4.49-4.48 (m, 1H, Cp), 4.41 (dd, *J* = 9.8, 6.9 Hz, 1H, H-8a), 4.40-4.38 (m, 2H, Cp), 4.20 (s, 5H, Cp′), 3.63-3.59 (m, 1H, H-6), 3.52-3.48 (m, 1H, H-6), 2.25-2.21 (m, 1H, H-8), 1.96-1.84 (m, 3H, H-7 and 8); $^{13}C\{^1H\}$ NMR (DMSO-d_6) δ 166.7 (C-1), 158.6 (C-4), 137.9 (C-1′), 131.2 (C-2′), 129.4 (C-3′), 129.2 (C-6′), 128.1 (C-3), 127.6 (C-4′ or 5′), 126.0 (C-4′ or 5′), 115.0 (CH=), 84.9 (Cp_{ipso}), 70.5 (Cp), 69.4 (Cp′), 69.0 (Cp), 68.3 (Cp), 67.6 (Cp), 58.4 (C-8a), 45.1 (C-6), 28.2 (C-8), 21.7 (C-7). IR (KBr, ν, cm^{-1}) 3191, 3081,

2955, 2874, 1694, 1629, 1435, 1382, 766, 742. ESI-MS (*m/z*) calc. for $C_{24}H_{22}FeN_2O_2$ 426.1 $[M]^+$, found 426.1 $[M]^+$. Elemental analysis calc. for $C_{24}H_{22}FeN_2O_2$ C 67.62, H 5.20, N 6.57 found C 67.56, H 5.48, N 6.56 %. R_f (HPLC) τ = 16.23 min. R_f (HPLC-chiral) τ_1 = 7.68 min (90%, (*S,Z*)-**4b**) τ_2 = 8.93 min (10%, (*R,Z*)-**4b**) (column Lux Cellulose-4; eluent: MeCN–water (9:1, v/v)).

(*S,Z*)- and (*S,E*)-3-(3-ferrocenylbenzylidene)heksahydropyrrolo[1,2-a]pyrazin-1,4-dione 4c

Compound **4c** was prepared in a total 55% yield (149 mg) starting from 271 mg (0.635 mmol) of **10c** according to general procedure E. The total reaction time was 90 min (3 × 30 min). The isomers (*S,Z*)- and (*R,Z*)-**4c** (113 mg, 41.7%, chiral HPLC analysis confirmed partial racemization, indicating a mixture of (*S,Z*):(*R,Z*) isomers in a 74:26 ratio) followed by (*S,E*)- and (*R,E*)-**4c** (36 mg, 13.3%) were isolated by column chromatography using a gradient of MeOH in DCM starting from 1% to 5% of MeOH (v/v) as eluent. Mixture of 74% of (*S,Z*)-**4c** and 26% of (*R,Z*)-**4c**. M.p. >205°C (dec). ^{1}H NMR (DMSO-d_6) δ 10.03 (br. s, 1H, NH), 7.65 (s, 1H, H-2′), 7.48-7.46 (m, 1H, H-5′), 7.40-7.38 (m, 1H, H-6′), 7.32 (t, J = 7.7 Hz, 1H, H-4′), 6.71 (s, 1H, CH=), 4.82 (t, J = 1.8 Hz, 2H, Cp), 4.42 (dd, J = 9.4, 6.5 Hz, 1H, H-8a), 4.35 (t, J = 1.8 Hz, 2H, Cp), 4.04 (s, 5H, Cp′), 3.58-3.54 (m, 1H, H-6), 3.50-3.46 (m, 1H, H-6), 2.24-2.20 (m, 1H, H-8), 1.97-1.84 (m, 3H, H-7 and 8). ^{13}C{^{1}H} NMR (DMSO-d_6) δ 167.2 (C-1), 158.6 (C-4), 139.4 (C-3′), 133.6 (C-1′), 128.6 (C-3), 128.5 (C-4′), 127.0 (C-2′), 126.6 (C-6′), 125.6 (C-5′), 114.9 (CH=), 84.5 (Cp_{ipso}), 69.4 (Cp′), 68.90 (Cp), 68.87 (Cp), 66.5 (Cp), 66.4 (Cp), 58.2 (C-8a), 45.2 (C-6), 27.9 (C-8), 21.6 (C-7). IR (KBr, ν, cm^{-1}) 3176, 3082, 2929, 2887, 1690, 1672, 1633, 1439, 1390, 805, 696; ESI-MS (*m/z*) calc. for $C_{24}H_{22}FeN_2O_2$ 426.1 $[M]^+$, found 426.1 $[M]^+$. Elemental analysis calc. for $C_{24}H_{22}FeN_2O_2$ C 67.62, H 5.20, N 6.57 found C 65.87, H 5.61, N 5.83%. R_f (HPLC) τ = 16.8 min. R_f (HPLC-chiral) τ_1 = 7.69 min (26%, (*R,Z*)-**4c**), τ_2

= 9.66 min (74%, (*S,Z*)-**4c**), (column Lux Cellulose-4; eluent: MeCN–water (9:1, v/v)). Mixture of (*S,E*)- and (*R,E*)-**4c** ^{1}H NMR (DMSO-d_6) δ 10.40 (br s, 1H, NH), 7.71 (s, 1H, H_{Ar}), 7.38 (d, J = 7.7 Hz, 1H, H_{Ar}), 7.31 (d, J = 7.8 Hz, 1H, H_{Ar}), 7.20 (t, J = 7.7 Hz, 1H, H_{Ar}), 6.38 (s, 1H, CH=), 4.72 (br. s, 2H, Cp), 4.40–4.37 (m, 1H, H-8a), 4.34-4.32 (m, 2H, Cp), 4.05 (s, 5H, Cp′), 3.54-3.50 (m, 1H, H-6), 3.45-3.41 (m, 1H, H-6), 2.23-2.20 (m, 1H, H-8), 1.93-1.80 (m, 3H, H-7 and 8). ESI-MS (*m*/*z*) calc. for $C_{24}H_{22}FeN_2O_2$ 426.1 $[M]^+$, found 426.1 $[M]^+$.

(*S,Z*)- and (*S,E*)-3-(4-ferrocenylbenzylidene)heksahydropyrrol[1,2-a]pyrazin-1,4-dione 4d

Compound **4d** was prepared in a total 38.4% yield (139 mg) starting from 362 mg (0.849 mmol) of **10d** according to general procedure E. The total reaction time was 60 min (2 × 30 min). The isomers (*S,Z*)- and (*R,Z*)-**4d** (126 mg, 34.8%, chiral HPLC analysis confirmed partial racemization, indicating a mixture of (*S,Z*):(*R,Z*) isomers in a 70:30 ratio) followed by (*S,E*)- and (*R,E*)-**4d** (13 mg, 3.6%) were isolated by column chromatography using a gradient of MeOH in DCM starting from 1% to 2% of MeOH (v/v) as eluent. Mixture of 70% of (*S,Z*)-**4d** and 30% of (*R,Z*)-**4d** M.p. >230°C (dec). ^{1}H NMR (DMSO-d_6) δ 10.06 (s, 1H, NH), 7.55 (d, J = 8.3 Hz, 2H, H-3′/5′), 7.47 (d, J = 8.3 Hz, 2H, H-2′/6′), 6.66 (s, 1H, CH=), 4.84-4.83 (m, 2H, Cp), 4.39 (dd, J = 9.5, 6.5 Hz, 1H, H-8a), 4.38 (t, J = 1.8 Hz, 2H, Cp), 4.04 (s, 5H, Cp′), 3.57-3.53 (m, 1H, H-6), 3.49-3.45 (m, 1H, H-6), 2.24-2.20 (m, 1H, H-8), 1.96-1.83 (m, 3H, H-7 and 8). $^{13}C\{^1H\}$ NMR (DMSO-d_6) δ 167.2 (C-1), 158.8 (C-4), 139.1 (C-1′), 130.9 (C-4′), 129.4 (C-2′/6′), 127.8 (C-3), 125.8 (C-3′/5′), 114.9 (CH=), 84.0 (Cp_{ipso}), 69.4 (Cp′), 69.19 (Cp), 69.17 (Cp), 66.4 (Cp), 66.3 (Cp), 58.2 (C-8a), 45.1 (C-6), 27.9 (C-8), 21.6 (C-7). IR (KBr, ν, cm^{-1}) 3282, 1686, 1664, 1623, 1605, 1434, 1386, 842, 827, 809, 512, 485. ESI-MS (*m*/*z*) calc. for $C_{24}H_{22}FeN_2O_2$ 426.1 $[M]^+$, found 426.1 $[M]^+$. Elemental analysis calc. for $C_{24}H_{22}FeN_2O_2$ C 67.62, H 5.20, N 6.57 found C 67.45, H 5.28, N 6.57 %. R_f (HPLC) τ=16.8 min. R_f (HPLC-

chiral) τ_1=10.7 min ((*R,Z*)-**4d**, 30%), τ_2=12.0 min ((*S,Z*)-**4d**, 70%), (column Lux Cellulose-4; eluent: MeCN–water (9:1, v/v)). Mixture of (*S,E*)- and (*R,E*)-**4a**. ^{1}H NMR (DMSO-d$_6$) δ 10.36 (s, 1H, H-2), 7.51 (d, *J* = 8.4 Hz, 2H, H-3′/5′), 7.44 (d, *J* = 8.4 Hz, 2H, H-2′/6′), 7.20 (s, 1H, CH=), 4.79 (t, *J* = 1.8 Hz, 2H, Cp), 4.37-4.34 (m, 3H, Cp and H-8a), 4.02 (s, 5H, Cp′), 3.55-3.50 (m, 1H, H-6), 3.50-3.42 (m, 1H, H-6), 2.21-2.19 (m, 1H, H-8), 2.02-1.83 (m, 3H, H-7 and 8). ESI-MS (*m/z*) calc. for $C_{24}H_{22}FeN_2O_2$ 426.1 $[M]^+$, found 426.1 $[M]^+$.

(*S,Z*)- and (*S,E*)-3-benzylideneheksahydropyrrol[1,2-a]pyrazin-1,4-dione 4e

Compound **4e** was prepared in a total 60.6% yield (194 mg) starting from 320 mg (1.32 mmol) of **10e** according to general procedure E. The total reaction time was 60 min (2 × 30 min). The isomers (*S,Z*)- and (*R,Z*)-**4e** (162 mg, 50.6%, chiral HPLC analysis confirmed partial racemization, indicating a mixture of (*S,Z*):(*R,Z*) isomers in a 74:26 ratio) followed by (*S,E*)- and (*R,E*)-**4e** (32 mg, 10%) were isolated by column chromatography using a gradient of MeOH in DCM starting from 1% to 3% of MeOH (v/v) as eluent. Mixture of 74% of (*S,Z*)-**4e** and 26% of (*R,Z*)-**4e** M.p. 162-163°C (dec.). ^{1}H NMR (DMSO-*d$_6$*) δ 10.04 (s, 1H, H-2), 7.54 (d, *J* = 7.4 Hz, 2H, H-2′/6′), 7.40 (t, *J* = 7.7 Hz, 2H, H-3′/5′), 7.30 (t, *J* = 7.4 Hz, 1H, H-4′), 6.67 (s, 1H, CH=), 4.39 (dd, *J* = 9.5, 6.6 Hz, 1H, H-8a), 3.57-3.52 (m, 1H, H-6), 3.48-3.44 (m, 1H, H-6), 2.23-2.19 (m, 1H, H-8), 1.96-1.82 (m, 3H, H-7 and 8). ^{13}C{^{1}H} NMR (DMSO-*d$_6$*) δ 167.7 (C-1), 158.6 (C-4), 133.5 (C-1″), 129.3 (C-2″/6″), 128.6 (C-3″/C-5″ and C-3), 127.9 (C-4″), 114.6 (CH=), 58.2 (C-8a), 45.2 (C-8), 28.0 (C-6), 21.6 (C-7). IR (KBr, ν, cm^{-1}) 3176, 3146, 3106, 2980, 1694, 1616, 1451, 1383, 1308, 695. ESI-MS (*m/z*) calc. for $C_{14}H_{15}N_2O_2$ $[M+H]^+$ 243.1, found 243.3 $[M+H]^+$. Elemental analysis calc. for $C_{14}H_{14}N_2O_2$ C 69.41, H 5.82, N 11.56, found C 69.34, H 5.76, N 11.69 %. R_f(HPLC) τ=6.3 min. R_f(HPLC-chiral) τ_1=14.2 min (26%, (*R,Z*)-**4e**), τ_2=22.9 min (74% (*S,Z*)-**4e**) (column Lux Cellulose-4; eluent: n-hexane–IPA (3:1, v/v)); Mixture of (*S,E*)- and (*R,E*)-**4e**. ^{1}H NMR (DMSO-d$_6$) δ 10.38 (br s, 1H, H-2), 7.51 (d, *J* = 7.5

Hz, 2H, H-2′/6′), 7.27 (t, J = 7.5 Hz, 2H, H-3′/5′), 7.22 (t, J = 7.3 Hz, 1H, H-4′), 6.35 (s, 1H, CH=), 4.36 (dd, J = 9.0, 6.9 Hz, 1H, H-8a), 3.52-3.48 (m, 1H, H-6), 3.43-3.39 (m, 1H, H-6), 2.21-2.18 (m, 1H, H-8), 1.92-1.85 (m, 3H, H-7 and 8). $^{13}C\{^{1}H\}$ NMR (DMSO-d_6) δ 167.3 (C-1), 157.4 (C-4), 134.2 (C-1′), 129.9 (C-2′/6′), 128.8 (C-3), 127.6 (C-3′/5′), 127.3 (C-4′), 119.2 (CH=), 58.4 (C-8a), 45.3 (C-8), 28.3 (C-6), 21.7 (C-7).

Crystallography

The X-ray intensity data for the abovementioned compounds were collected on an Agilent Supernova 4 circle diffractometer system equipped with either molybdenum (Mo Kα) or silver (Ag Kα) microsource and an Eos CCD detector. Data were collected at 100K for **4e**, due to weak diffraction; the remaining samples were investigated at RT. Low temperature of a sample was maintained, when necessary, by placing it in a cold nitrogen stream, using Oxford Cryosystems cooling devices. The X-ray intensity data were integrated with the CrysAlis171[4] software; this process included correction for absorption effects using the multiscan method (SCALE3 ABSPACK[5]).

The structures were solved by direct methods (SXELXS[6]) or intrinsic phasing (SHELXT[7]) or charge flipping (olex.solve[8]) and refined using the full-matrix least squares procedure with SHELXL[9] within the OLEX2[8] graphical interface. Figures were generated using the OLEX2[8] software. Structural data were deposited with CCDC and assigned the deposition numbers CCDC 2207258–2207261.

Biological Assays

Cell lines

The normal lung fibroblast MRC-5 and colorectal adenocarcinoma SW620 cell lines used in this experiment were purchased from American Type Culture Collection (USA) via LGC

Standards. Multidrug-resistant variants of SW620 cells were developed in our laboratory as described previously.[10, 11] All cells were cultured under standard conditions (37°C, 5% CO_2, 100% humidity) in high-glucose DMEM supplemented with GlutaMaxTM, HEPES (ThermoFisher Scientific, USA), and 10% fetal bovine serum (EURx, Poland). Cells were routinely tested for potential *Mycoplasma* contamination using the MycoProbe Mycoplasma Detection kit (R&D System, USA).

Viability assay [12]

In total, 10^4 cells in 100 µL of complete medium were plated per well of a 96-well plate and left overnight to allow cells to adhere to the surface. Then, the cells were exposed to a desired concentration of tested compound(s). Stock solutions were prepared in DMSO and used immediately. The final DMSO concentration was constant and nontoxic (0.1% v/v). After 70-h incubation, neutral red was added to the final concentration of 1 mM. After another 2 h, the medium was aspirated, and cells were washed twice with PBS. Dye was released using 100 µL of a solubilizer (1% acetic acid in 50% ethanol) on an orbital shaker (10 min). Absorbance was read at 540 nm using EnVision Multilabel Plate Reader (Perkin Elmer, USA). Results were expressed as % of control. The IC_{50} parameters were calculated using GraphPad Prism version 10 (GraphPad Software, Boston, Massachusetts USA).

The enhancement of reversal potency (ERP) value determination

For MDR reversal assays, cytotoxic agents were co-administered with compounds **4a–4e** at equimolar concentrations in a range of 10 nM to 100 µM. IC_{50} values were determined in ABCB1-overexpressing SW620 cell line variants. The enhancement of reversal potency (ERP) was calculated using the following formula: ERP = IC_{50}(drug)MDR / IC_{50}(drug + compound)MDR. All experiments were performed in triplicate (n = 3).

Transport assays

SW620 cells and their drug-resistant variants overexpressing ABCB1 (SW620V), ABCC1 (SW620M), or ABCG2 (SW620Mito) were trypsinized and suspended in a complete cell-culture medium prewarmed to 37°C at a final density of approximately 1×10^6/mL. The tested compounds were added from DMSO stock solutions to a final concentration of 10 μM. Respective samples were supplemented with a specific fluorescent marker (100 nM calcein AM for ABCB1, 100 nM BCECF AM for ABCC1, and 1 μM pheophorbide A for ABCG2). For the control, given protein-specific inhibitors (10 μM verapamil for ABCB1, 25 μM MK-571 for ABCC1, and 10 μM Ko143 for ABCG2) were added to parallel, tested compound-free, samples. For ABCB1 and ABCG2 assays (dye accumulation assays), intracellular fluorescence was measured by flow cytometry (FACSymphony A1, Becton, Dickinson and Company, New Jersey, USA) immediately after dye addition (time 0) and every 3–5 min up to approximately 30 min using a flow cytometer set at 488 nm excitation and 530/30 nm emission for the calcein/ABCB1 assay and 400 nm excitation and 655/8 nm emission for the pheophorbide A/ABCG2 assay. For the BCECF/ABCC1 assay (dye exclusion assay), the sample was preincubated at 37°C for 10 min, centrifuged (400 × *g*, 10 min, RT), and then resuspended in fresh medium. The intracellular BCECF concentration was then immediately measured at 488 nm excitation and 530/30 nm emission (time 0). Further measurements were performed at 15-min intervals. All samples were incubated at 37°C between measurements. The accumulation/exclusion curves were plotted, and the slope of the initial accumulation rate or dye retention half time was evaluated using the GraphPad Prism software version 10 (GraphPad Software, Boston, Massachusetts USA). The difference between the accumulation rate/half time value for dye only and the tested compound/inhibitor samples was expressed as a given protein activity measure.

Antibody-binding assays

A total of 10^6 parental SW620 cells expressing low level of ABC proteins or their multidrug-resistant variant SW620V cells (overexpressing ABCB1) or SW620Mito cells (overexpressing ABCG2) were suspended in 1 mL of complete culture medium supplemented with solvent (DMSO), a positive control (vincristine/mitoxantrone), or an investigated compound at a final concentration of 1 μM. APC-conjugated UIC2 anti-ABCB1/FITC-conjugated 5D3 anti-ABCG2 antibody or the respective isotype control was then added to a final concentration of 5 μg/mL, and the samples were incubated at 37°C for 60 min. Then, the cells were washed three times (100 × *g* for 10 min) with fresh medium and analyzed using a FACSymphony A1 (Becton, Dickinson and Company, New Jersey, USA) instrument. Results were expressed as a difference of median of fluorescence of a given sample (specific binding of antibody) and the respective isotype control (unspecific binding).

Synergy determination.

To evaluate the potential interactions between putative ABC inhibitors and anticancer drugs, we utilized the Bliss independence model, as implemented in the SynergyFinder web application.[13, 14] Synergy scores and the most synergistic area scores were calculated based on the normalized dose–response matrices. A synergy score below −10 indicates likely antagonistic interactions, a score between −10 and 10 suggests additive effects, and a score above 10 is indicative of likely synergistic interactions.

Statistical analysis.

All experiments were performed at least three times, and the data were statistically analyzed using Prism (GraphPad).

Additional figures

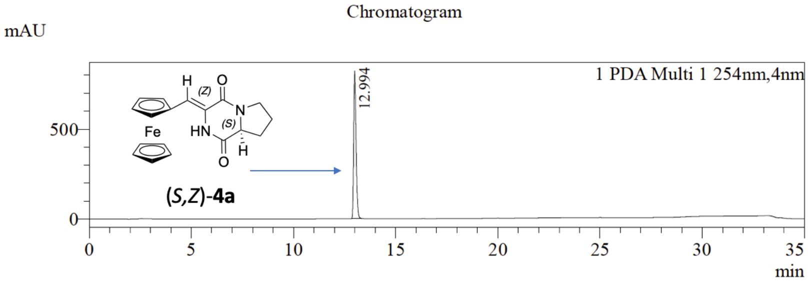

Method

<<Header>>

<<Pump>>
Pump A : LC-20AD
Total Flow : 1.0000 mL/min
B Conc. : 20.0 %

<<Autosampler>>
Autosampler Model : SIL-20A

<<Data Acquisition>>

<<Mobile Phase Name>>
Pump A Mobile Phase A : 0,1%TFA water
Pump A Mobile Phase B : 0,1% TFA MeCN

<<Column Infomation>>
Column Name : Kinetex 5u PFP 100A
Column ID : 00F-4602-E0
Column Length : 150 mm
Inner Diameter : 4.0 mm

Sample Information

Sample Name	: kch-70051F1_1
Time Acquired	: 8:40:58 AM
Time Processed	: 9:49:56 AM
Injection Volume	: 5

Method

<<LC Time Program>>

Time	Module	Command	Value	Comm
0.30	Pumps	B.Conc	20	
15.00	Pumps	B.Conc	80	
20.00	Pumps	B.Conc	80	
21.00	Pumps	B.Conc	100	
25.00	Pumps	B.Conc	100	
26.00	Pumps	B.Conc	20	
35.00	Controller	Stop		

Figure S1. HPLC chromatogram of (*S*,*Z*)-**4a**

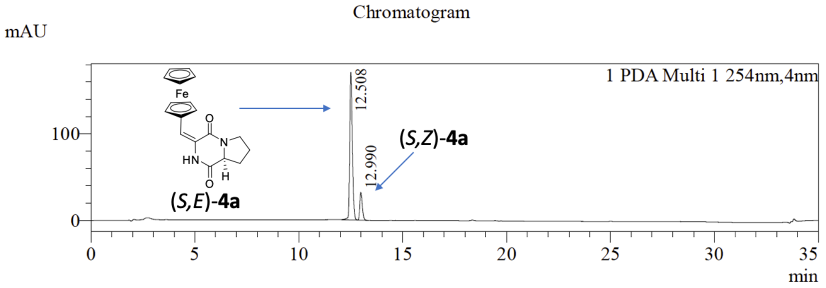

Method

<<Header>>

<<Pump>>
Pump A : LC-20AD
Total Flow : 1.0000 mL/min
B Conc. : 20.0 %

<<Autosampler>>
Autosampler Model : SIL-20A

<<Data Acquisition>>

<<Mobile Phase Name>>
Pump A Mobile Phase A : 0,1%TFA water
Pump A Mobile Phase B : 0,1% TFA MeCN

<<Column Infomation>>
Column Name : Kinetex 5u PFP 100A
Column ID : 00F-4602-E0
Column Length : 150 mm
Inner Diameter : 4.0 mm

Sample Information

Sample Name	: kch-70051F2_1
Time Acquired	: 9:22:43 AM
Time Processed	: 9:14:44 AM
Injection Volume	: 5

Method

<<LC Time Program>>

Time	Module	Command	Value	Comm
0.30	Pumps	B.Conc	20	
15.00	Pumps	B.Conc	80	
20.00	Pumps	B.Conc	80	
21.00	Pumps	B.Conc	100	
25.00	Pumps	B.Conc	100	
26.00	Pumps	B.Conc	20	
35.00	Controller	Stop		

Figure S2. HPLC chromatogram of (*S*,*E*)-**4a**

Chromatogram

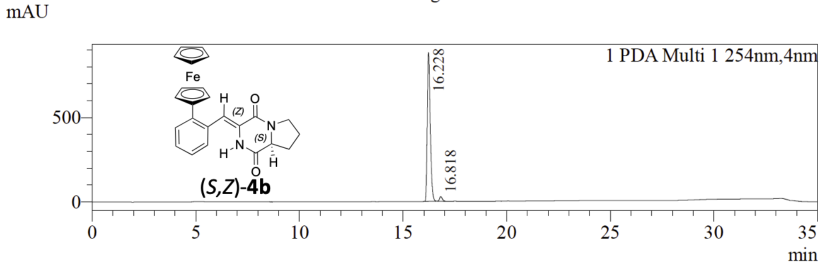

Method

<<Header>>	
<<Pump>>	
Pump A	: LC-20AD
Total Flow	: 1.0000 mL/min
B Conc.	: 20.0 %
<<Autosampler>>	
Autosampler Model	: SIL-20A
<<Data Acquisition>>	
<<Mobile Phase Name>>	
Pump A Mobile Phase A	: 0,1%TFA water
Pump A Mobile Phase B	: 0,1% TFA MeCN
<<Column Infomation>>	
Column Name	: Kinetex 5u PFP 100A
Column ID	: 00F-4602-E0
Column Length	: 150 mm
Inner Diameter	: 4.0 mm

Sample Information

Sample Name	: kch-70083F1_1
Time Acquired	: 9:43:10 AM
Time Processed	: 8:01:27 AM
Injection Volume	: 10

Method

<<LC Time Program>>

Time	Module	Command	Value	Comm
0.30	Pumps	B.Conc	20	
15.00	Pumps	B.Conc	80	
20.00	Pumps	B.Conc	80	
21.00	Pumps	B.Conc	100	
25.00	Pumps	B.Conc	100	
26.00	Pumps	B.Conc	20	
35.00	Controller	Stop		

Figure S3. HPLC chromatogram of (*S*,*Z*)-**4b**

Chromatogram

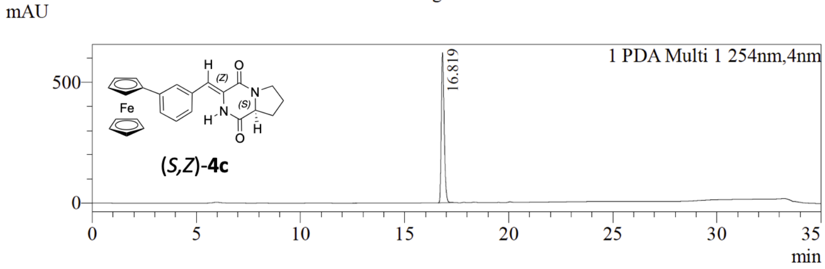

Method

<<Header>>	
<<Pump>>	
Pump A	: LC-20AD
Total Flow	: 1.0000 mL/min
B Conc.	: 20.0 %
<<Autosampler>>	
Autosampler Model	: SIL-20A
<<Data Acquisition>>	
<<Mobile Phase Name>>	
Pump A Mobile Phase A	: 0,1%TFA water
Pump A Mobile Phase B	: 0,1% TFA MeCN
<<Column Infomation>>	
Column Name	: Kinetex 5u PFP 100A
Column ID	: 00F-4602-E0
Column Length	: 150 mm
Inner Diameter	: 4.0 mm

Sample Information

Sample Name	: kch-70078F1_1
Time Acquired	: 7:55:56 AM
Time Processed	: 9:48:10 AM
Injection Volume	: 5

Method

<<LC Time Program>>

Time	Module	Command	Value	Comm
0.30	Pumps	B.Conc	20	
15.00	Pumps	B.Conc	80	
20.00	Pumps	B.Conc	80	
21.00	Pumps	B.Conc	100	
25.00	Pumps	B.Conc	100	
26.00	Pumps	B.Conc	20	
35.00	Controller	Stop		

Figure S4. HPLC chromatogram of (*S*,*Z*)-**4c**

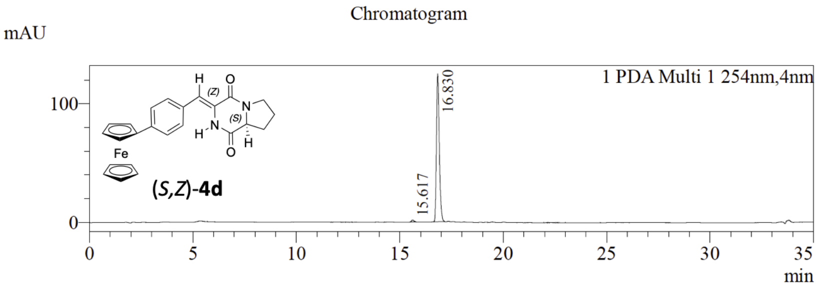

Sample Information

Sample Name	: kch-70080F1_1
Time Acquired	: 9:08:21 AM
Time Processed	: 9:47:41 AM
Injection Volume	: 5

Method

<<Header>>

<<Pump>>

Pump A	: LC-20AD
Total Flow	: 1.0000 mL/min
B Conc.	: 20.0 %

<<Autosampler>>

Autosampler Model	: SIL-20A

<<Data Acquisition>>

<<Mobile Phase Name>>

Pump A Mobile Phase A	: 0,1%TFA water
Pump A Mobile Phase B	: 0,1% TFA MeCN

<<Column Infomation>>

Column Name	: Kinetex 5u PFP 100A
Column ID	: 00F-4602-E0
Column Length	: 150 mm
Inner Diameter	: 4.0 mm

Method

<<LC Time Program>>

Time	Module	Command	Value	Comm
0.30	Pumps	B.Conc	20	
15.00	Pumps	B.Conc	80	
20.00	Pumps	B.Conc	80	
21.00	Pumps	B.Conc	100	
25.00	Pumps	B.Conc	100	
26.00	Pumps	B.Conc	20	
35.00	Controller	Stop		

Figure S5. HPLC chromatogram of (*S*,*Z*)-**4d**

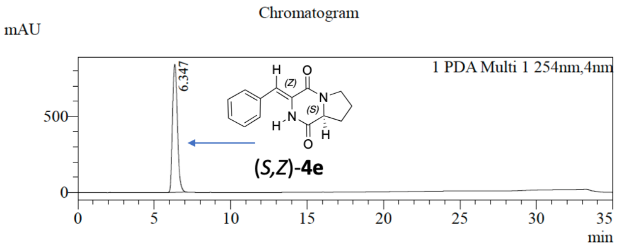

Sample Information

Sample Name	: kch-70096F1_1
Time Acquired	: 10:19:07 AM
Time Processed	: 8:36:54 PM
Injection Volume	: 10

Method

<<Pump>>

Pump A	: LC-20AD
Total Flow	: 1.0000 mL/min
B Conc.	: 20.0 %

<<Mobile Phase Name>>

Pump A Mobile Phase A	: 0,1%TFA water
Pump A Mobile Phase B	: 0,1% TFA MeCN

<<Column Infomation>>

Column Name	: Kinetex 5u PFP 100A
Column Length	: 150 mm
Inner Diameter	: 4.0 mm

<<PDA>>

Start Wavelength	: 200 nm
End Wavelength	: 600 nm

Method

<<LC Time Program>>

Time	Module	Command	Value
0.30	Pumps	B.Conc	20
15.00	Pumps	B.Conc	80
20.00	Pumps	B.Conc	80
21.00	Pumps	B.Conc	100
25.00	Pumps	B.Conc	100
26.00	Pumps	B.Conc	20
35.00	Controller	Stop	

Figure S6. HPLC chromatogram of (*S*,*Z*)-**4e**

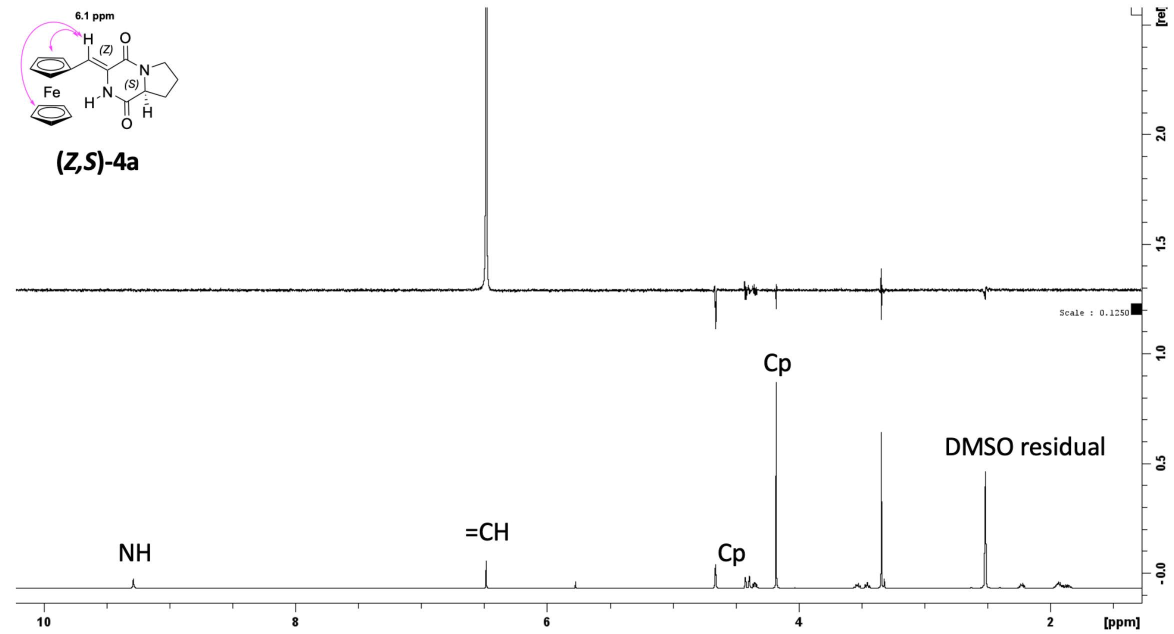

Figure S7. selROESY spectrum (top) of **(Z,S)-4a** irradiation at H-1′ signal (δ = 6.1 ppm)

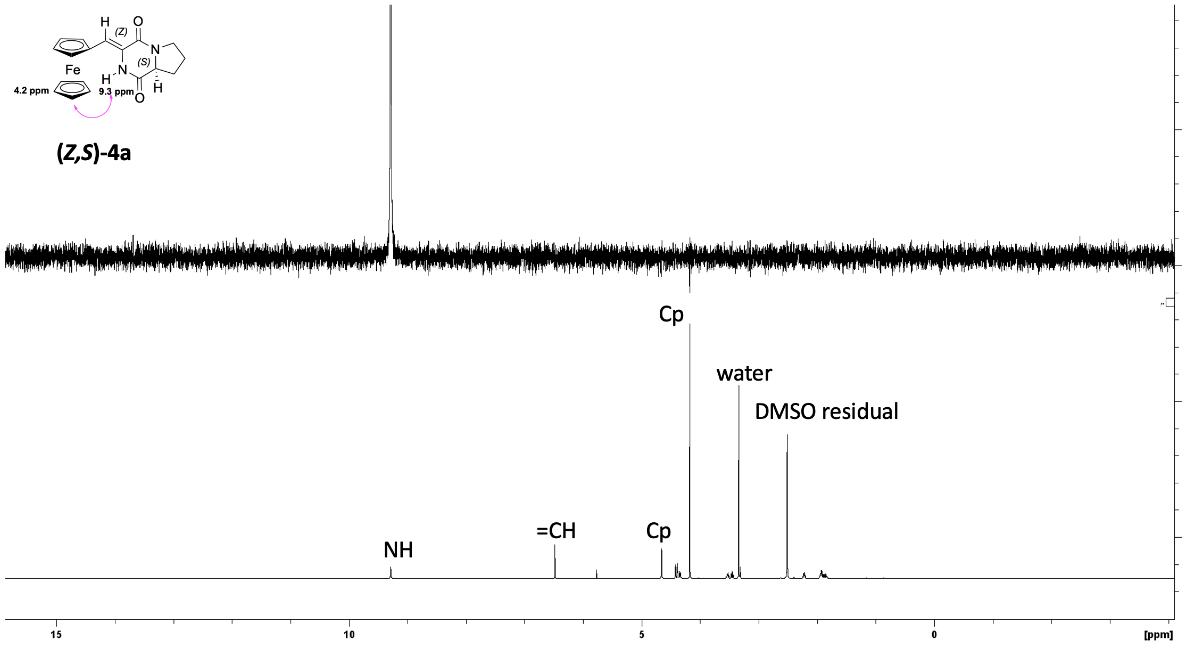

Figure S8. selROESY spectraum (top) of (*Z,S*)-**4a** irradiation at NH signal (δ = 9.3 ppm)

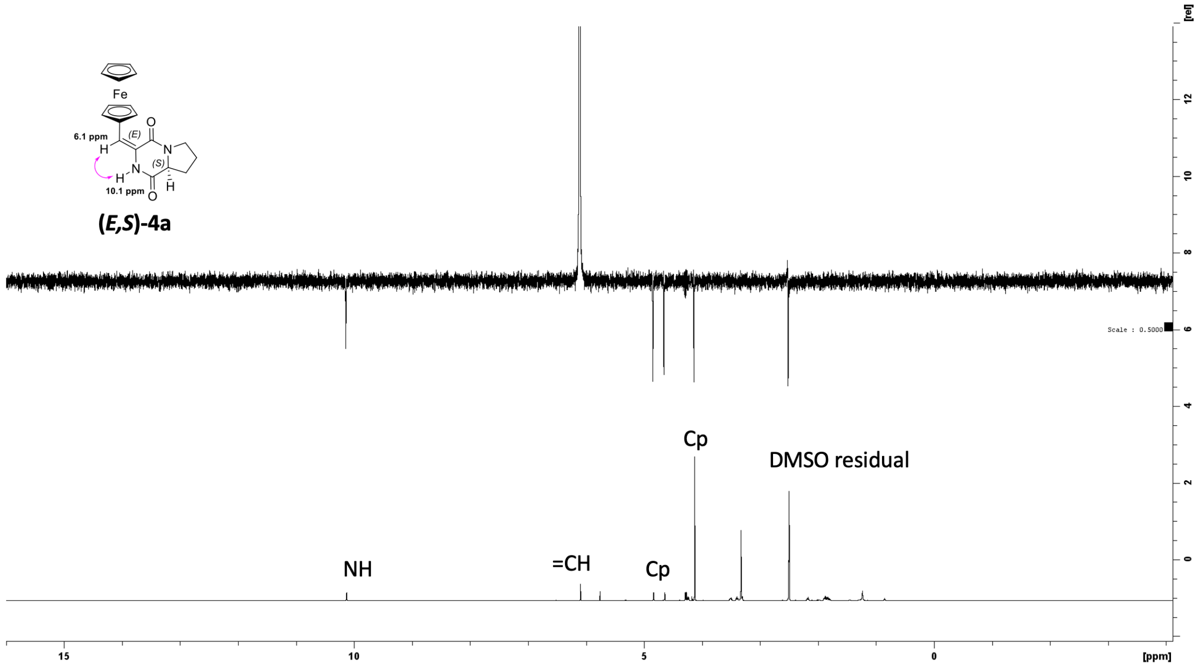

Figure S9. selROESY spectrum (top) of (*E,S*)-**4a** irradiation at H-1′ signal (δ = 6.1 ppm)

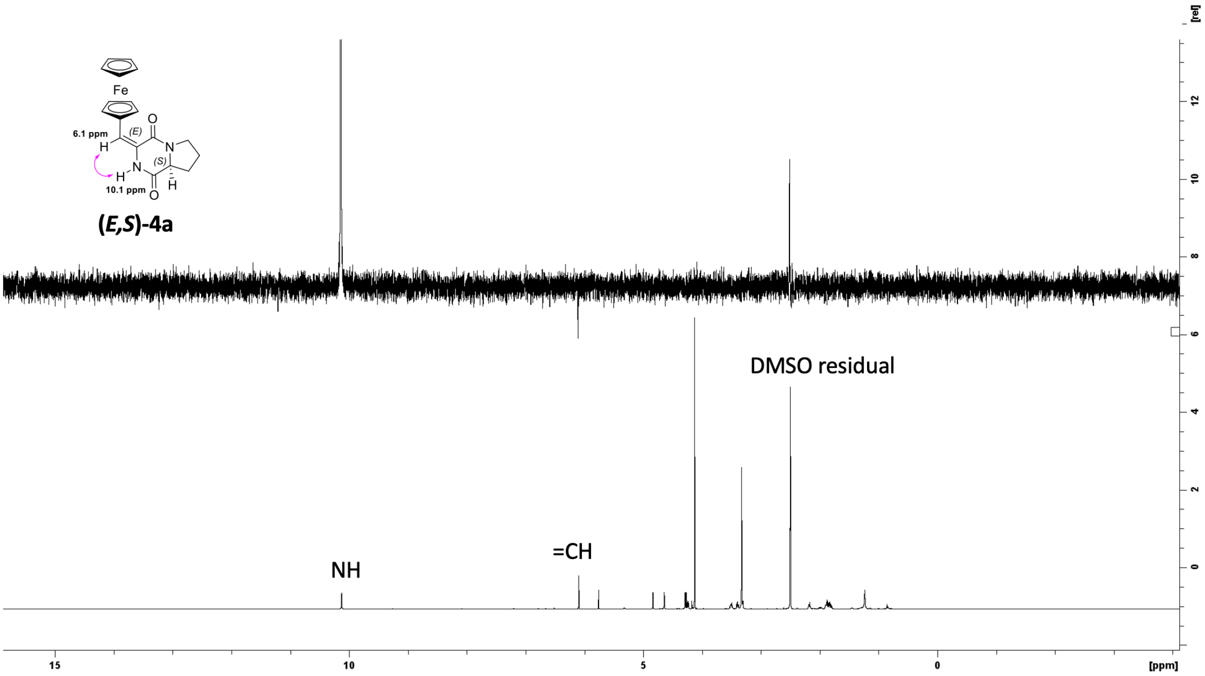

Figure S10. selROESY spectrum (top) of (*E,S*)-**4a** irradiation at H-1′ signal (δ = 10.1 ppm)

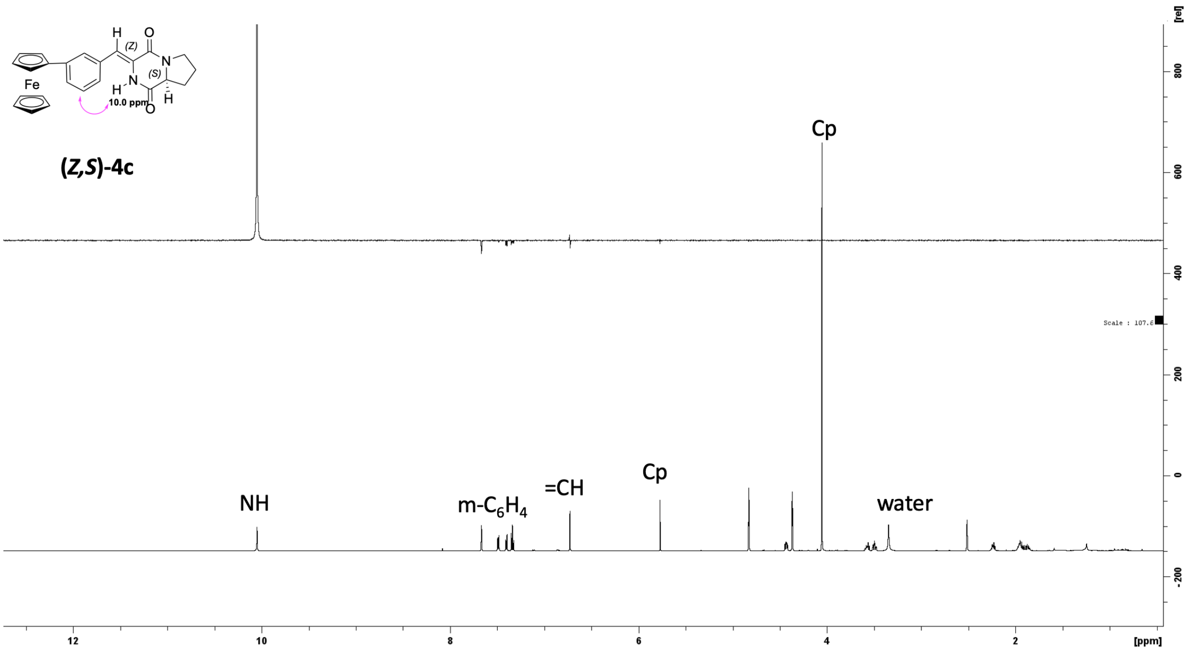

Figure S11. selROESY spectrum (top) of (*Z,S*)-**4c** irradiation at NH signal (δ = 10.0 ppm)

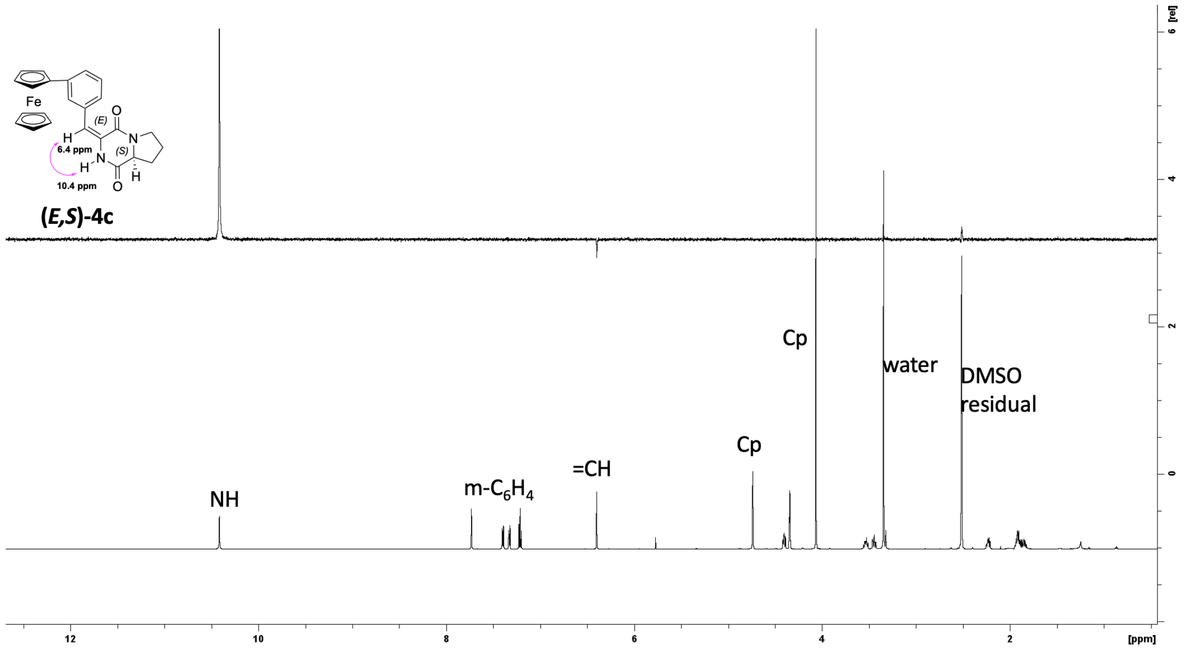

Figure S12. selROESY spectrum (top) of (*E,S*)-**4c** irradiation at NH signal (δ = 10.4 ppm)

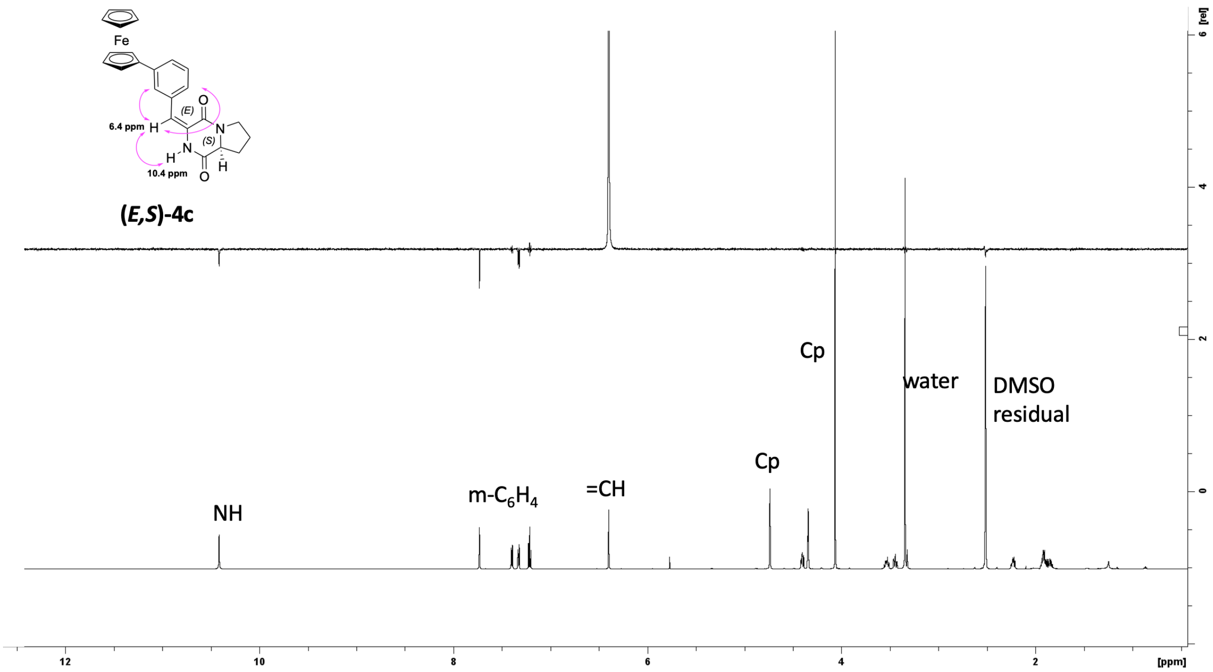

Figure S13. selROESY spectrum (top) of (*E,S*)-**4c** irradiation at H-1′ signal (δ = 6.4 ppm)

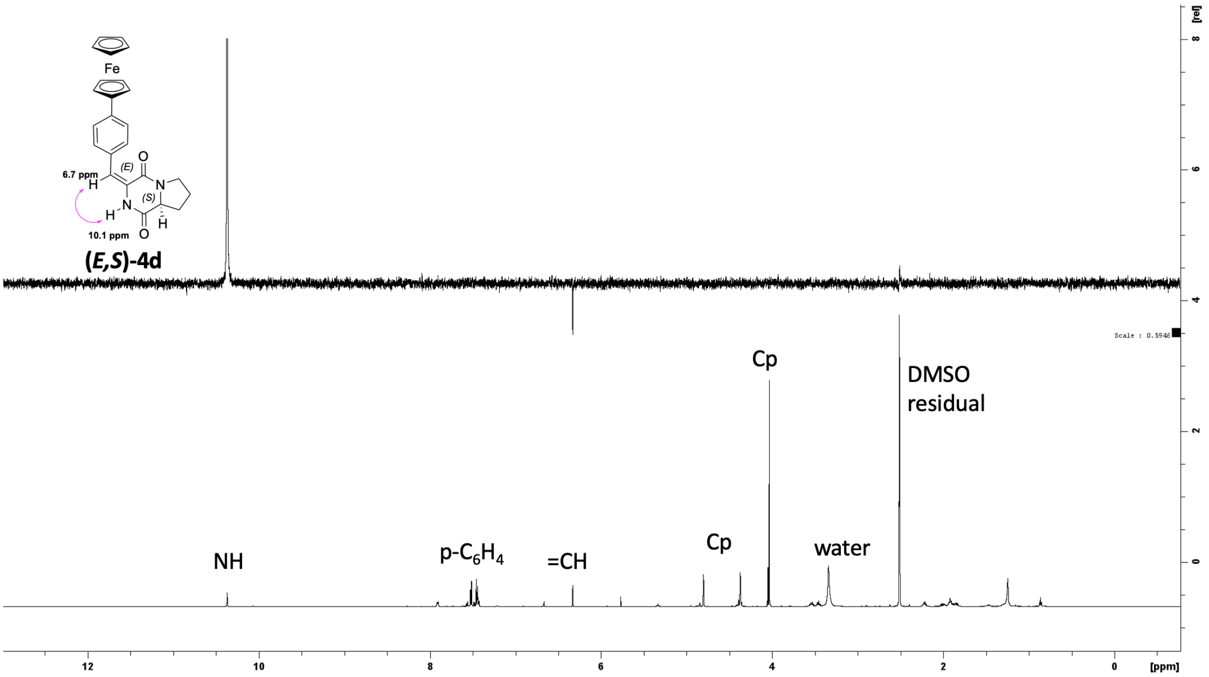

Figure S14. selROESY spectrum (top) of (*E,S*)-**4d** irradiation at NH signal (δ = 10.1 ppm)

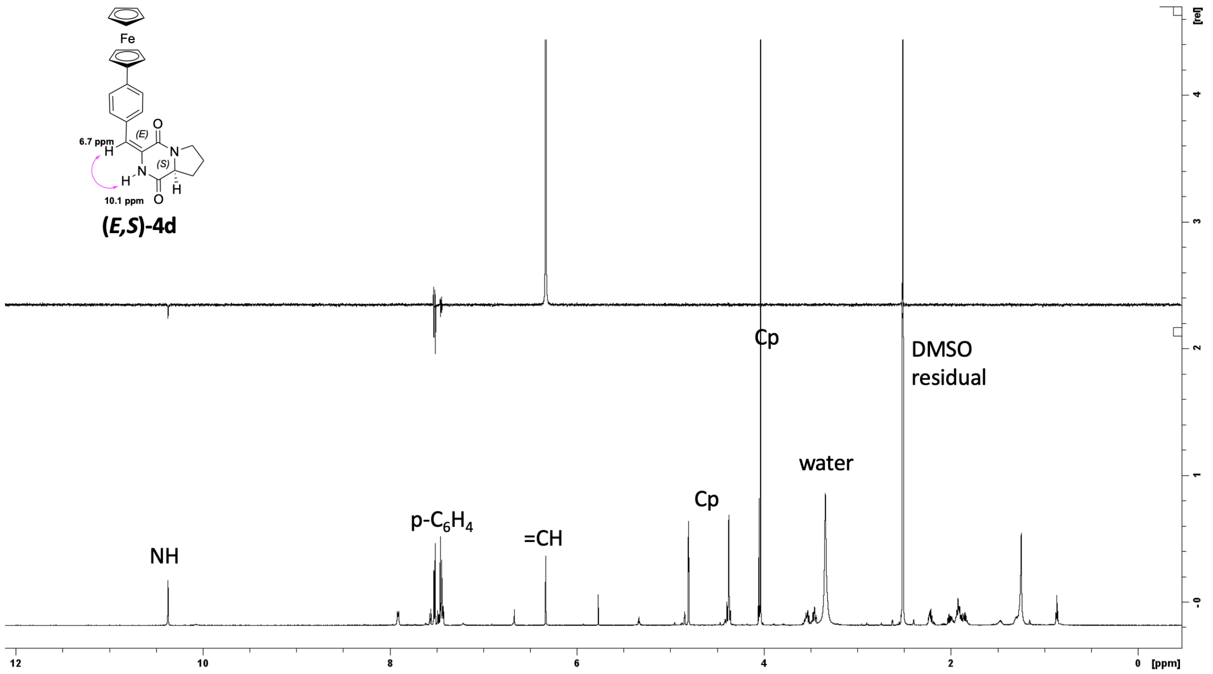

Figure S15. selROESY spectrum (top) of (*E*,*S*)-**4d** irradiation at H-1′ signal (δ = 6.7 ppm)

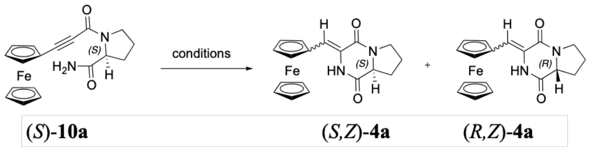

Conditions
a) Me_3P, Glu, DMF-toluene (1:6), 80 °C, 12 h
Yield of **4a** 4 %

Lux Cellulose-3
n-hexane-IPA

b) Me_3P, Glu, DMF-toluene (1:6), MW, 120 °C, 60 min
Yield of **4a** 54 %

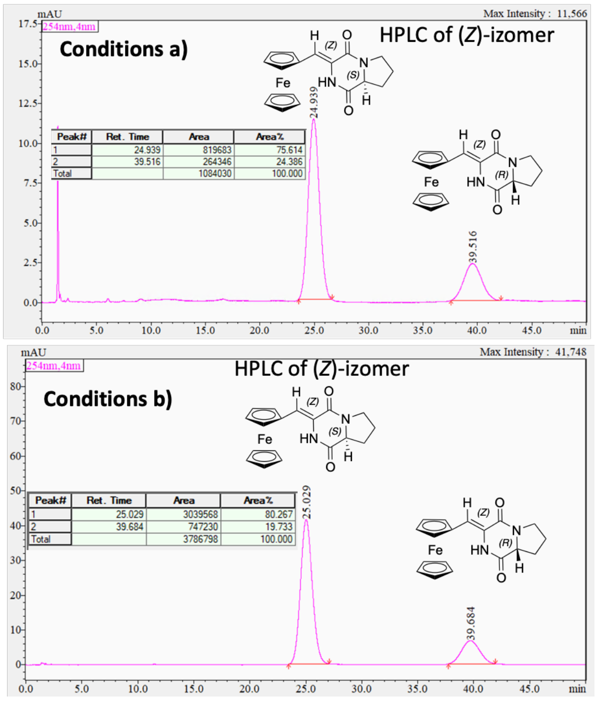

Peak#	Ret. Time	Area	Area%
1	24.939	819683	75.614
2	39.516	264346	24.386
Total		1084030	100.000

Peak#	Ret. Time	Area	Area%
1	25.029	3039568	80.267
2	39.684	747230	19.733
Total		3786798	100.000

Figure S16. The impact of the reaction condition on the reaction yields and racemization during the synthesis of (*S*,*Z*)-**4a**.

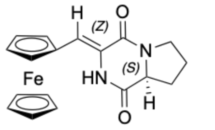

(*S*,*Z*)-**4a**

Lux Cellulose-4
MeCN-water 9:1

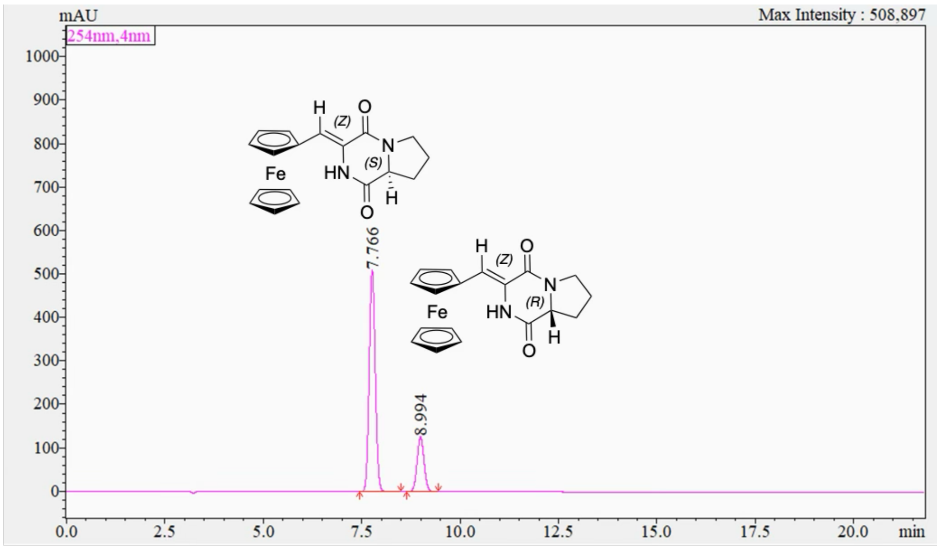

Peak#	Ret. Time	Area	Area%
1	7.766	5503364	77.963
2	8.994	1555605	22.037
Total		7058969	100.000

Figure S17. HPLC chromatogram of (*S*,*Z*)-**4a** on Lux Cellulose-4

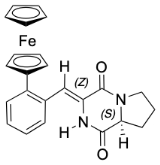

(*S*,*Z*)-**4b**

Lux Cellulose-4
MeCN-water 9:1

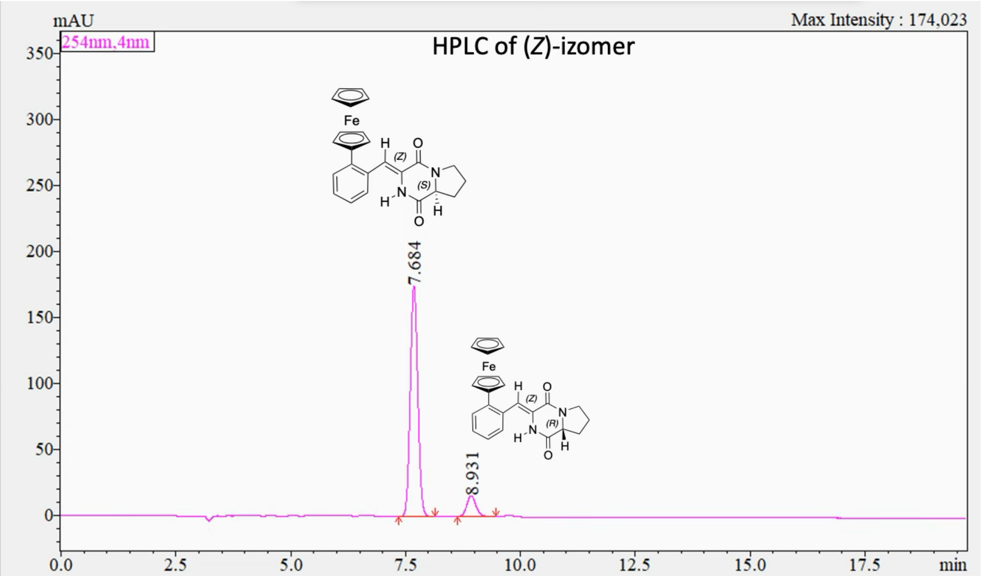

Peak#	Ret. Time	Area	Area%
1	7.684	1986342	89.987
2	8.931	221026	10.013
Total		2207368	100.000

Figure S18. HPLC chromatogram of (*S*,*Z*)-**4b** on Lux Cellulose-4

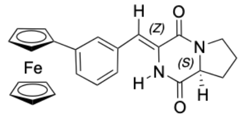

(*S*,*Z*)-**4c**

Lux Cellulose-4
MeCN-water 9:1

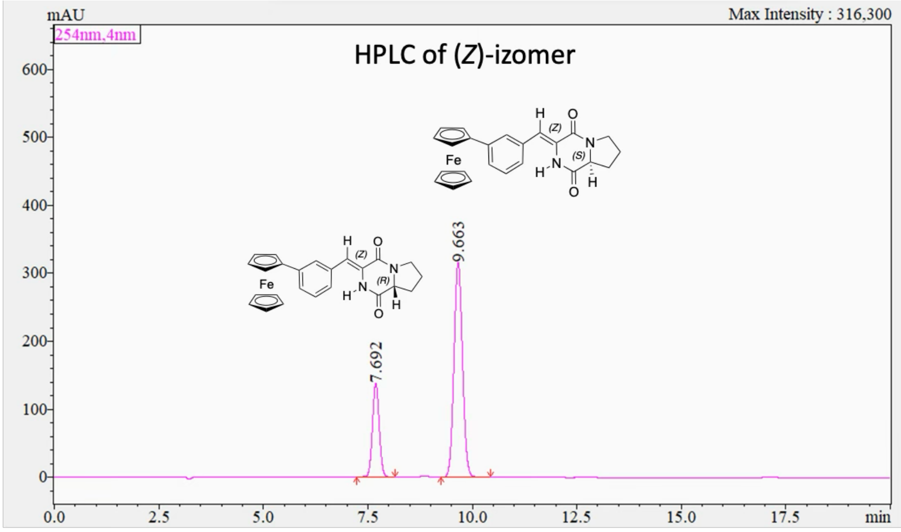

Peak#	Ret. Time	Area	Area%
1	7.692	1558862	25.795
2	9.663	4484416	74.205
Total		6043278	100.000

Figure S19. HPLC chromatogram of (*S*,*Z*)-**4c** on Lux Cellulose-4

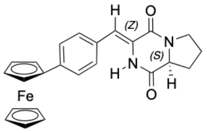

(*S*,*Z*)-**4d**

Lux Cellulose-4
MeCN-water 9:1

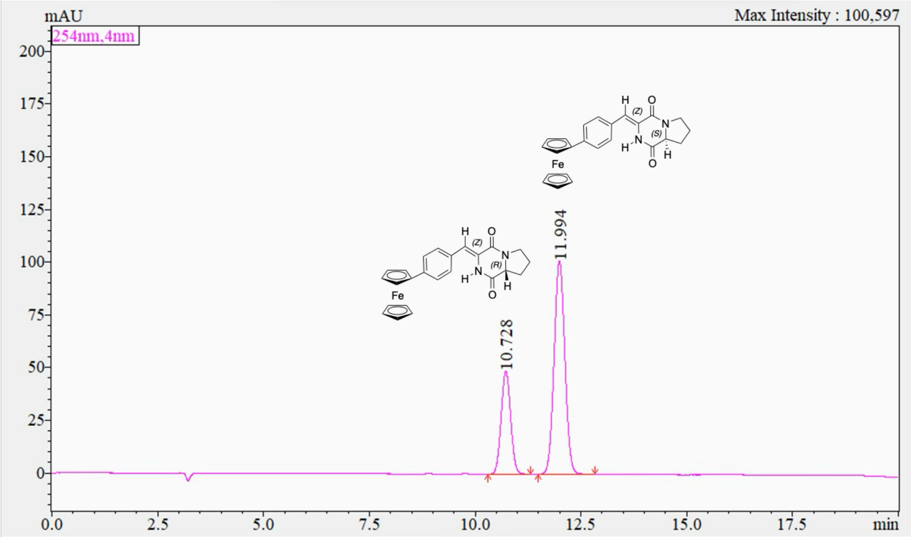

Peak#	Ret. Time	Area	Area%
1	10.728	766938	30.032
2	11.994	1786822	69.968
Total		2553761	100.000

Figure S20. HPLC chromatogram of (*S*,*Z*)-**4d** on Lux Cellulose-4

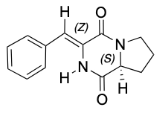

(*S*,*Z*)-**4e**

Lux Cellulose-4
n-hexane-IPA 3:1

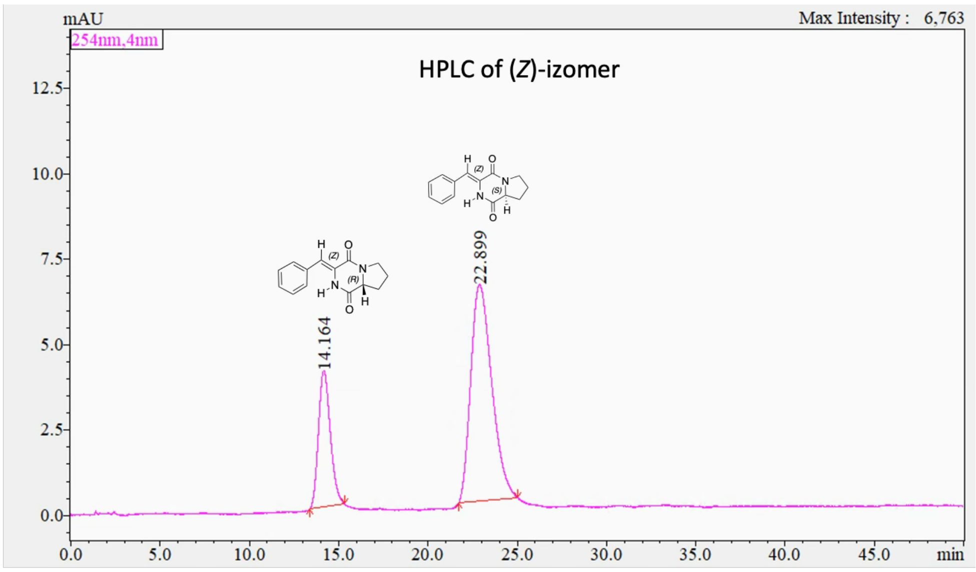

Peak#	Ret. Time	Area	Area%
1	14.164	184086	26.333
2	22.899	514990	73.667
Total		699075	100.000

Figure S21. HPLC chromatogram of (*S*,*Z*)-**4e** on Lux Cellulose-4

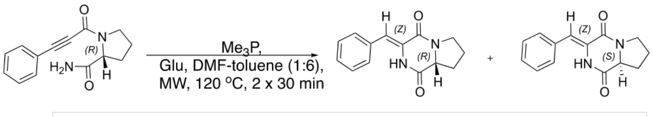

(*R*)-**10e** (*R*,*Z*)-**4e** (*S*,*Z*)-**4a**

Peak#	Ret. Time	Area	Area%
1	14.093	707962	64.742
2	22.943	385544	35.258
Total		1093506	100.000

Lux celulose 3
n-hexane-IPA

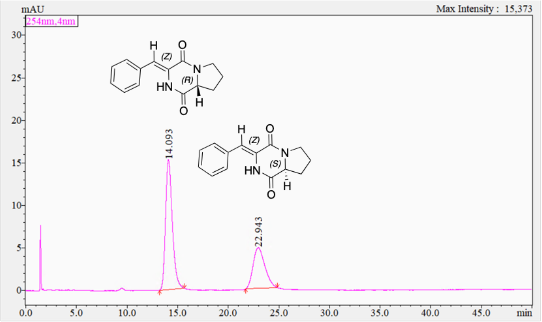

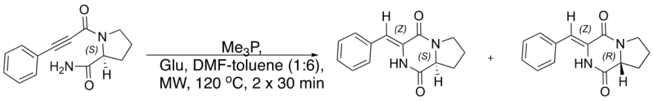

(*S*)-**10e** (*S*,*Z*)-**4e** (*R*,*Z*)-**4a**

Peak#	Ret. Time	Area	Area%
1	14.124	196756	31.389
2	22.854	430078	68.611
Total		626835	100.000

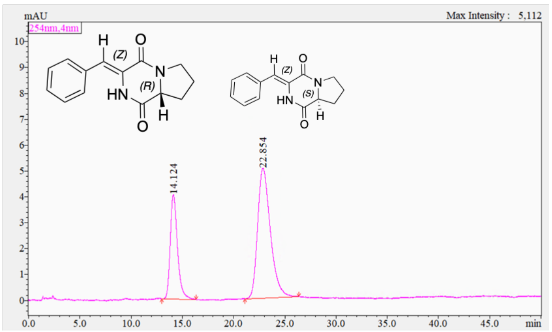

Figure S22. HPLC analysis of **4e** obtained by cyclization of (*R*)- or (*S*)-**11e** under optimized reaction conditions.

X-ray data

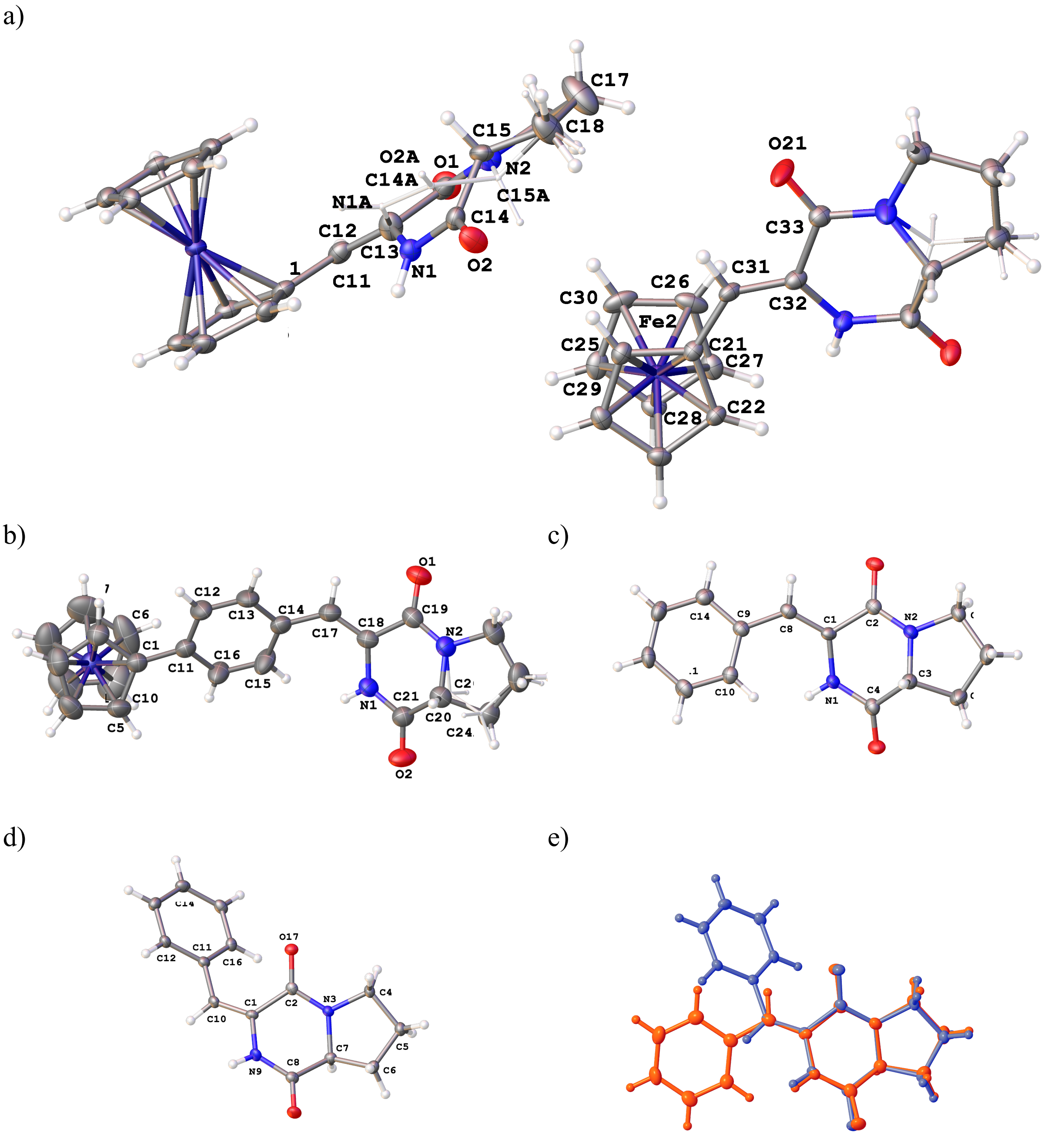

Figure S23. ORTEP representation of investigated compounds in their crystal structures. H atom labels inherited from the adjacent C/N atoms and omitted in graphics for clarity. Atomic displacement parameters represented at 50% probability level. Minor conformers of the disordered structures represented as light thin lines; a) **4a**, b) **4d**, c) **4e** (*P*4$_2$bc), d) **(E)-4e** (*P*2$_1$), e) overlay of molecular conformations of **4e** from *P*4$_2$bc (orange) and *P*2$_1$ (blue) polymorphic structures.

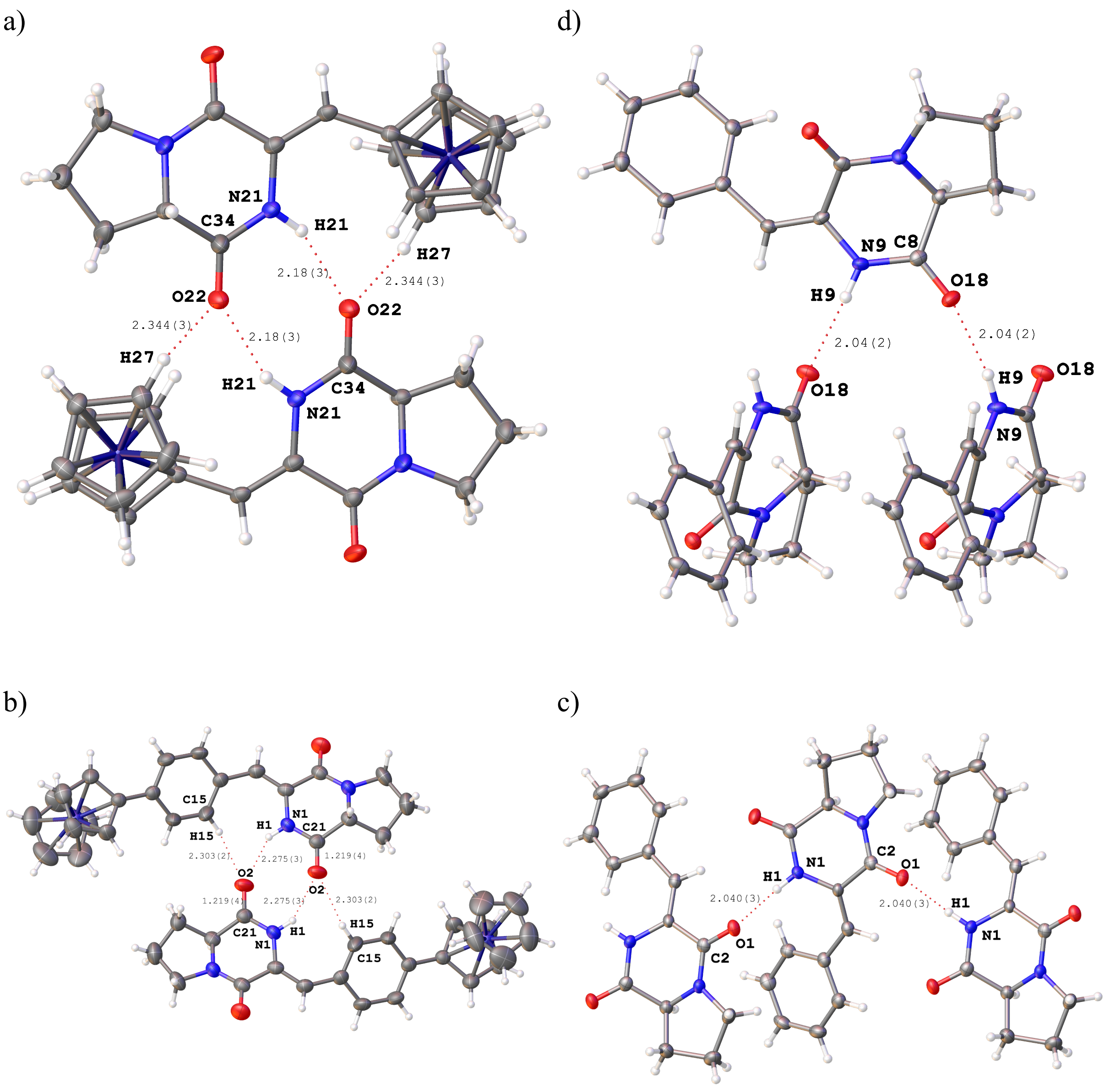

Figure S24. H-bond motifs present in the crystal structures of a) **4a**, b) **4d**, c) **4e** ($P4_2bc$), d) **(E)-4e** ($P2_1$). Closed ring motifs are present for the ferrocenyl compounds, while two distinct infinite ribbons are formed in the case of **4e** in its two polymorphs. All H atoms were visible in the residual density maps, but were added geometrically and refined mostly in riding approximation.

Table S1. Crystallographic Data

**Compound**	**(*Z*)-4a**	**(Z)-4d**	**(*Z*)-4e**	**(*E*)-4e**
Empirical formula	$C_{18}H_{18}FeN_2O_2$	$C_{24}H_{22}FeN_2O_2$	$C_{14}H_{14}N_2O_2$	$C_{14}H_{14}N_2O_2$
CCDC number	2207259	2207260	2207261	2207258
Formula weight	350.19	426.28	242.27	242.27
Crystal system	triclinic	monoclinic	tetragonal	monoclinic
Space group	*P* -1	*P* 2_1/c	*P* 4_2 b c	*P* 2_1
**R : S enantiomer ratio**	**1:1**	**1:1**	**1:1**	**7:3**
a/Å	10.1533(4)	13.6466(14)	14.8061(5)	7.4145(4)
b/Å	12.7077(6)	18.9059(13)	14.8061(5)	6.2908(3)
c/Å	13.2049(7)	7.7181(7)	11.0059(5)	12.5846(8)
α/°	63.588(5)	90	90	90
β/°	86.501(4)	101.178(9)	90	93.759(5)
γ/°	83.708(4)	90	90	90
Volume/Å^3	1516.59(14)	1953.5(3)	2412.71(19)	585.72(6)
Z	4	4	8	2
ρ_{calc} mg/mm^3	1.534	1.449	1.334	1.374
F(000)	728	888	1024	256
μ/mm^{-1}	1.006	0.795	0.091	0.094
Max. transmission	1	1	1	1
Min. transmission	0.84116	0.724	0.59993	0.69567
Absorption corr.	multi-scan	gaussian	multi-scan	multi-scan
Crystal color	orange	dark orange	colourless	colourless
Crystal habit	plate	plate	needle	needle
Crystal size/mm	0.41<br>0.23<br>0.04	0.34<br>0.237<br>0.053	0.50<br>0.06<br>0.06	0.64<br>0.09<br>0.024
R_{int}	0.0532	0.0389	0.0681	0.0387
R_{sigma}	0.0516	0.083	0.0411	0.0484
Index ranges: h	14 -14	18 -18	20 -20	10 -10
k	18 -18	25 -26	20 -21	8 -8
l	17 -17	5 -10	14 -15	16 -16
Reflections collected	37099	9586	40682	10908
2Θ range : max	30.56	29.835	30.539	30.43
min	2.576	2.154	2.751	2.753
Temperature/K	100.0(1)	294(2)	100.0(1)	100.0(1)
X-ray wavelength/Å	0.71073	0.71073	0.71073	0.71073
Independent refl.	6733	3112	2969	2729

I > 2 σ(I)				
Independent refl.	8433	4988	3505	3174
Largest diff.:<br>peak /e $Å^{-3}$	0.714	0.437	0.265	0.252
hole /e $Å^{-3}$	-0.717	-0.432	-0.225	-0.252
Extinction coefficient	.	.	0.0033(8)	.
Goodness-of-fit on F^2	1.164	1.06	1.07	1.08
Parameters	471	281	168	166
Data	8433	4988	3505	3174
Restraints	8	39	2	2
R1 all data	0.0806	0.1181	0.0615	0.058
R1 [I>=2σ (I)]	0.0604	0.0682	0.0455	0.0448
wR2 all data	0.1242	0.1246	0.098	0.0998
wR2 [I>=2σ (I)]	0.1164	0.1061	0.0909	0.0923
Flack parameter	n/a	n/a	1.00(2)	-0.7(7)

Additional biological data

Table S2. Effect of coadministration of **4a-e** and doxorubicin, etoposide, or vincristine at equal concentration, expressed as IC_{50} value [µM] of drugs in ABCB1-overexpressing SW620 cell line variants; ERP values were calculated using the following formula: ERP = $IC_{50(drug)MDR}/IC_{50(drug+\mathbf{4})MDR}$. n = 3

**Doxorubicin (Dox) (ABCB1)**			**ERP**	**Etoposide (VP16) (ABCB1)**			**ERP**	**Vincristine (VINC) (ABCB1)**			**ERP**
	**SW620**	**SW620D**			**SW620**	**SW620E**			**SW620**	**SW620V**	
**Dox**	0.342 (0.288–0.405)	4.26 (3.74–4.85)	-	**VP16**	2.60 (2.23–3.04)	16.0 (14.5–17.6)	-	**VINC**	0.125 (0.096–0.162)	24.3 (16.7–35.2)	-
**Dox+** (*S,Z*)-**4a**	0.396 (0.333–0.470)	1.47 (1.27–1.68)	**2.90**	**VP16+** (*S,Z*)-**4a**	3.44 (3.18–3.73)	2.85 (2.11–3.86)	**5.61**	**VINC+** (*S,Z*)-**4a**	0.108 (0.085–0.135)	4.05 (3.01–5.44)	**6.00**
**Dox+** (*S,Z*)-**4b**	0.342 (0.288–0.405)	1.82 (1.57–2.11)	**2.34**	**VP16+** (*S,Z*)-**4b**	1.83 (1.56–2.15)	7.21 (6.28–8.26)	**2.22**	**VINC+** (*S,Z*)-**4b**	0.117 (0.102–0.134)	2.54 (1.98–3.26)	**9.57**
**Dox+** (*S,Z*)-**4c**	0.144 (0.134–0.155)	1.75 (1.46–2.10)	**2.43**	**VP16+** (*S,Z*)-**4c**	3.50 (2.91–4.20)	9.92 (7.93–12.4)	1.61	**VINC+** (*S,Z*)-**4c**	0.073 (0.059–0.090)	3.67 (3.07–4.38)	**6.62**
**Dox+** (*S,E*)-**4c**	0.344 (0.280–0.421)	5.56 (4.86–6.35)	0.77	**VP16+** (*S,E*)-**4c**	3.22 (2.83–3.66)	18.4 (16.2–21.0)	0.87	**VINC+** (*S,E*)-**4c**	0.126 (0.094–0.170)	30.1 (20.3–47.8)	0.81
**Dox+** (*S,Z*)-**4d**	0.381 (0.300–0.485)	1.49 (1.23–1.80)	**2.86**	**VP16+** (*S,Z*)-**4d**	2.06 (1.85–2.30)	2.74 (2.04–3.66)	**5.84**	**VINC+** (*S,Z*)-**4d**	0.127 (0.090–0.179)	1.92 (1.52–2.42)	**12.7**
**Dox+** (*S,Z*)-**4e**	0.408 (0.324–0.512)	6.11 (4.85–7.73)	0.70	**VP16+** (*S,Z*)-**4e**	3.28 (2.79–3.86)	18.2 (15.5–21.4)	0.88	**VINC+** (*S,Z*)-**4e**	0.125 (0.099–0.157)	19.0 (13.3–28.1)	1.27
**Dox+** (*S,E*)-**4e**	0.598 (0.563–0.635)	5.55 (4.81–6.42)	0.77	**VP16+** (*S,E*)-**4e**	2.71 (2.38–3.10)	18.1 (15.8–20.7)	0.88	**VINC+** (*S,E*)-**4e**	0.127 (0.101–0.159)	17.7 (13.9–22.4)	1.37

Table S3. Effect of coadministration of **4a-e** and methotrexate, cisplatin, or mitoxantrone at equal concentration, expressed as IC_{50} value [μM] of drugs in ABCC1- or ABCG2-overexpressing SW620 cell line variants; ERP values were calculated using the following formula: $ERP = IC_{50(drug)MDR}/IC_{50(drug+\mathbf{4})MDR}$. n = 3.

**Methotrexate (MTX) ABCC1**			**ERP**	**Cisplatin (cDDP) ABCG2**			**ERP**	**Mitoxantrone (Mito) ABCG2**			**ERP**
	**SW620**	**SW620M**			**SW620**	**SW620C**			**SW620**	**SW620Mito**	
**MTX**	6.28 (5.55–7.10)	54.1 (45.3–64.5)	-	**cDDP**	4.34 (3.51–5.38)	27.9 (25.8–30.3)	-	**Mito**	0.151 (0.130–0.176)	6.36 (5.07–8.04)	-
**MTX+ (*S,Z*)-4a**	10.5 (8.10–13.5)	46.1 (39.6–53.7)	1.17	**cDDP+ (*S,Z*)-4a**	3.89 (3.16–4.79)	5.74 (5.22–6.30)	**4.86**	**Mito+ (*S,Z*)-4a**	0.158 (0.128–0.195)	1.38 (1.08–1.78)	**4.61**
**MTX+ (*S,Z*)-4b**	9.83 (8.68–11.1)	29.4 (25.1–34.8)	**1.84**	**cDDP+ (*S,Z*)-4b**	4.93 (4.06–6.00)	6.16 (5.41–7.01)	**4.53**	**Mito+ (*S,Z*)-4b**	0.172 (0.148–0.199)	0.698 (0.615–0.793)	**9.11**
**MTX+ (*S,Z*)-4c**	9.66 (7.85–11.9)	32.8 (27.9–38.9)	**1.65**	**cDDP+ (*S,Z*)-4c**	3.59 (2.88–4.49)	1.83 (1.56–2.14)	**15.2**	**Mito+ (*S,Z*)-4c**	0.120 (0.104–0.138)	1.41 (1.13–1.75)	**4.51**
**MTX+ (*S,E*)-4c**	9.98 (8.55–11.7)	46.7 (39.1–56.8)	1.16	**cDDP+ (*S,E*)-4c**	4.84 (3.89–6.03)	8.09 (7.09–9.23)	**3.45**	**Mito+ (*S,E*)-4c**	0.111 (0.093–0.132)	7.31 (6.02–8.90)	0.87
**MTX+ (*S,Z*)-4d**	6.08 (5.52–6.69)	64.2 (54.9–76.5)	0.84	**cDDP+ (*S,Z*)-4d**	3.40 (2.79–4.16)	10.8 (9.03–12.9)	**2.58**	**Mito+ (*S,Z*)-4d**	0.164 (0.131–0.205)	0.619 (0.537–0.713)	**10.3**
**MTX+ (*S,Z*)-4e**	10.5 (9.01–12.2)	42.3 (36.0–50.5)	1.28	**cDDP+ (*S,Z*)-4e**	4.15 (3.31–5.23)	39.8 (34.5–46.2)	0.70	**Mito+ (*S,Z*)-4e**	0.160 (0.130–0.198)	6.31 (5.15–7.80)	1.01
**MTX+ (*S,E*)-4e**	6.91 (6.03–7.93)	50.1 (42.5–60.2)	1.08	**cDDP+ (*S,E*)-4e**	4.42 (3.33–5.90)	14.1 (13.8–20.0)	**1.98**	**Mito+ (*S,E*)-4e**	0.118 (0.103–0.136)	7.75 (6.02–10.0)	0.82

Copies of the NMR spectra

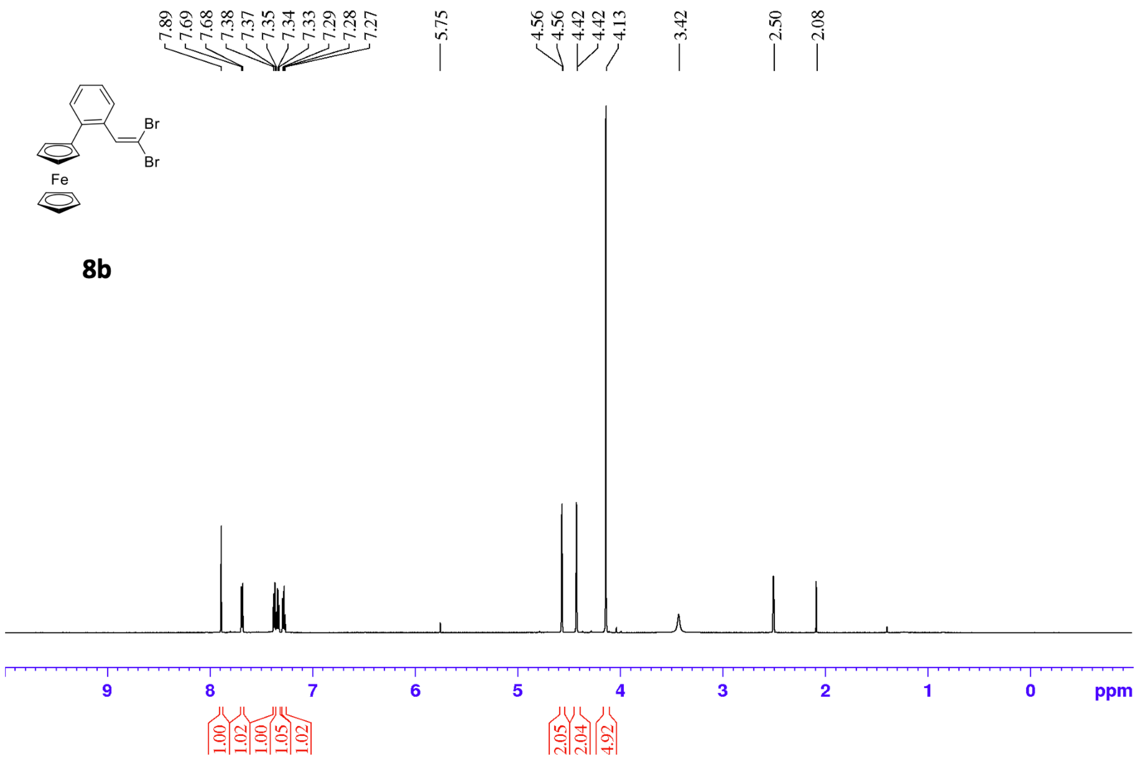

Figure S26. ^{1}H NMR spectrum of **8b** in DMSO-d$_6$

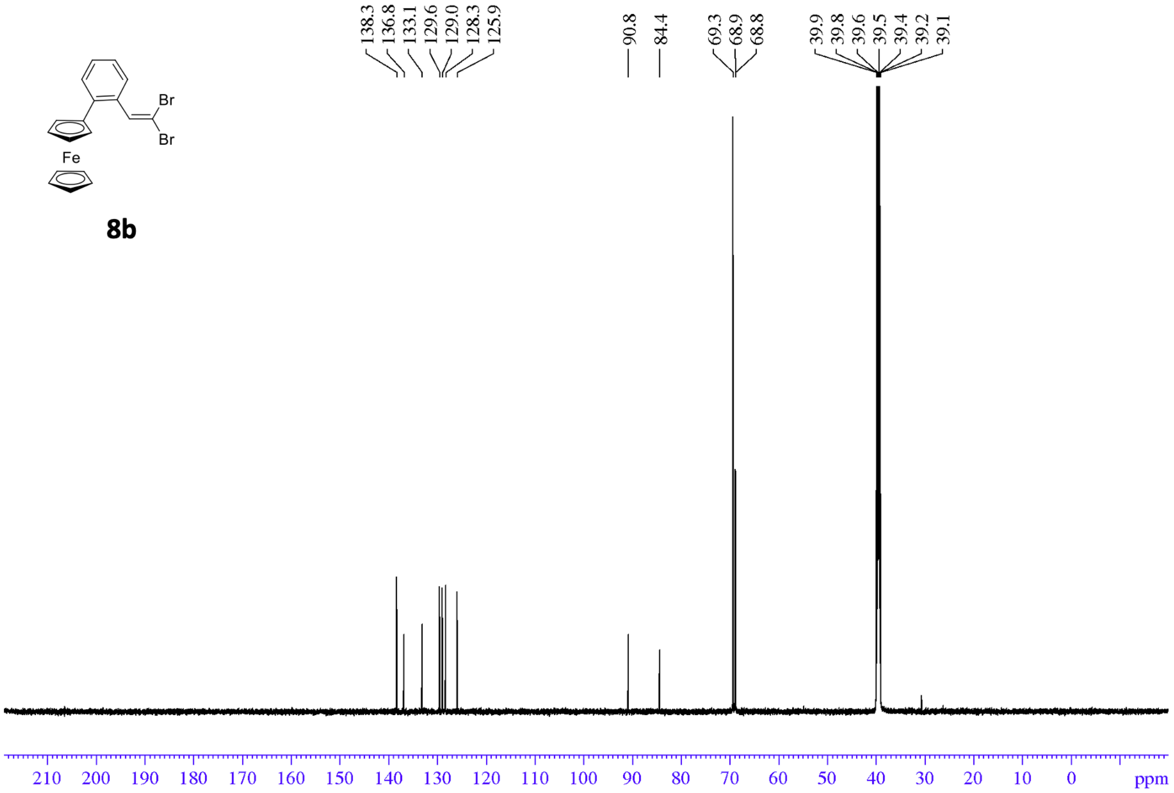

Figure S27. $^{13}C\{^{1}H\}$ NMR spectrum of **8b** in DMSO-d$_6$

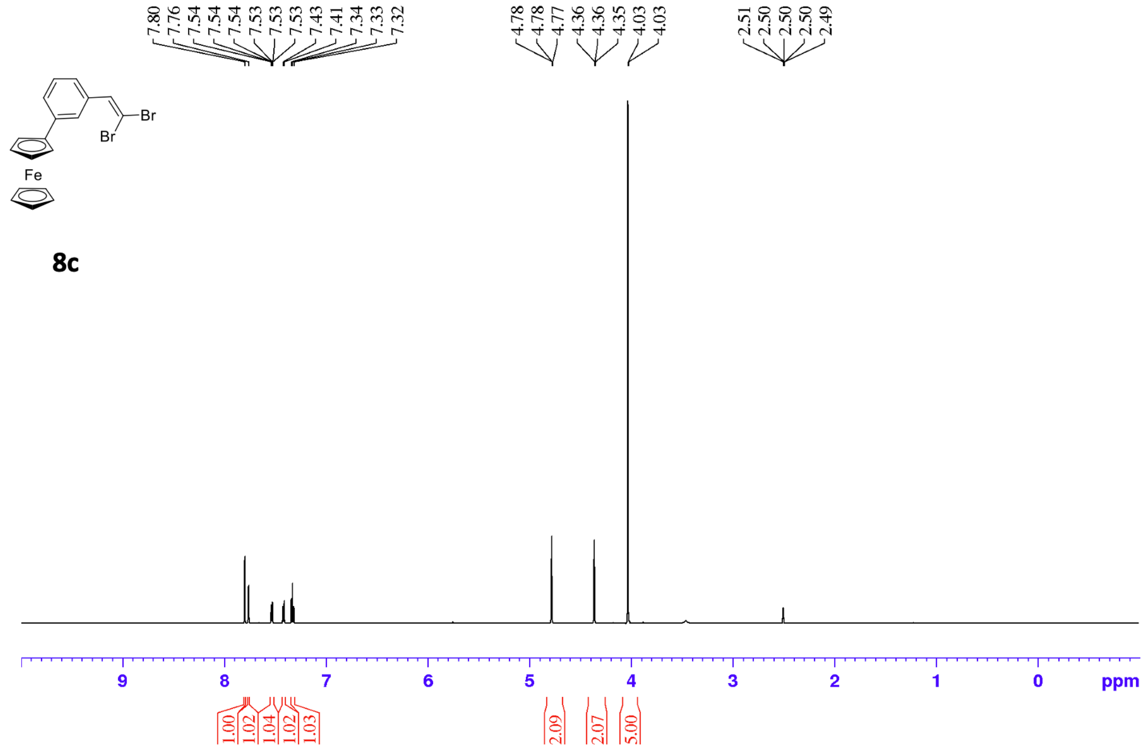

Figure S28. ^{1}H NMR spectrum of **8c** in DMSO-d$_6$

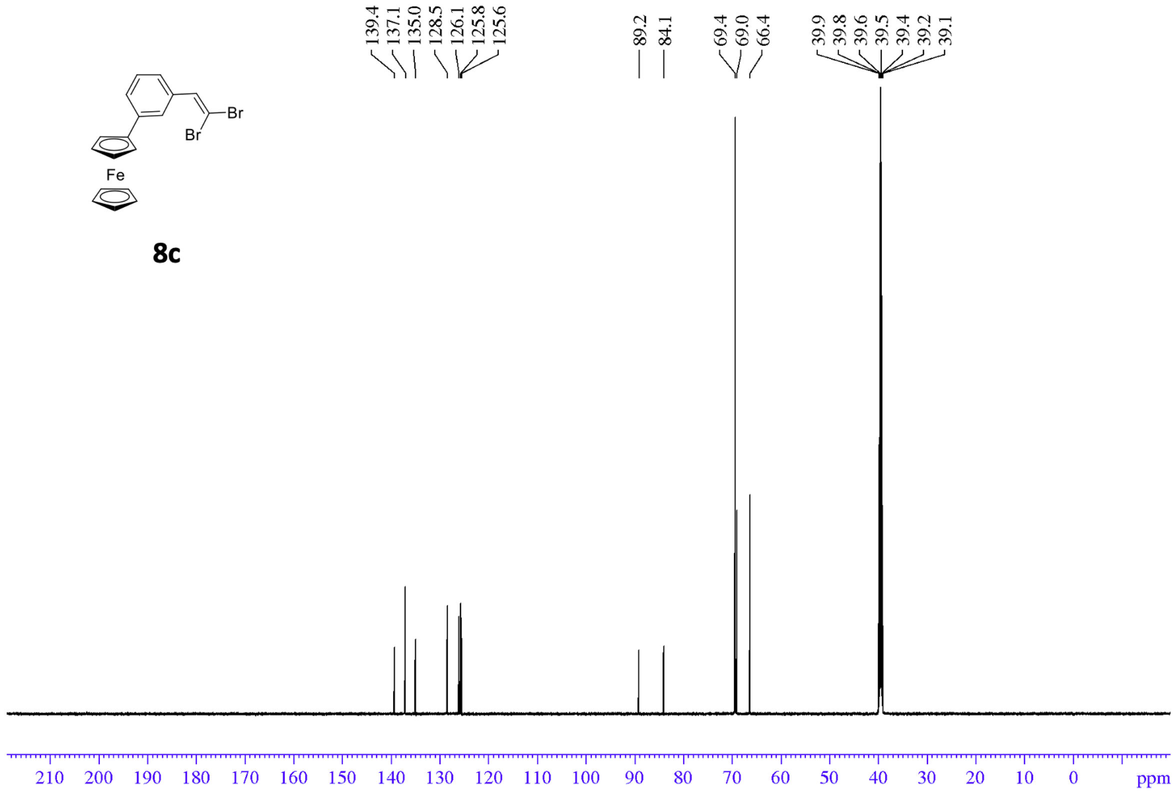

Figure S29. $^{13}C\{^{1}H\}$ NMR spectrum of **8c** in DMSO-d$_6$

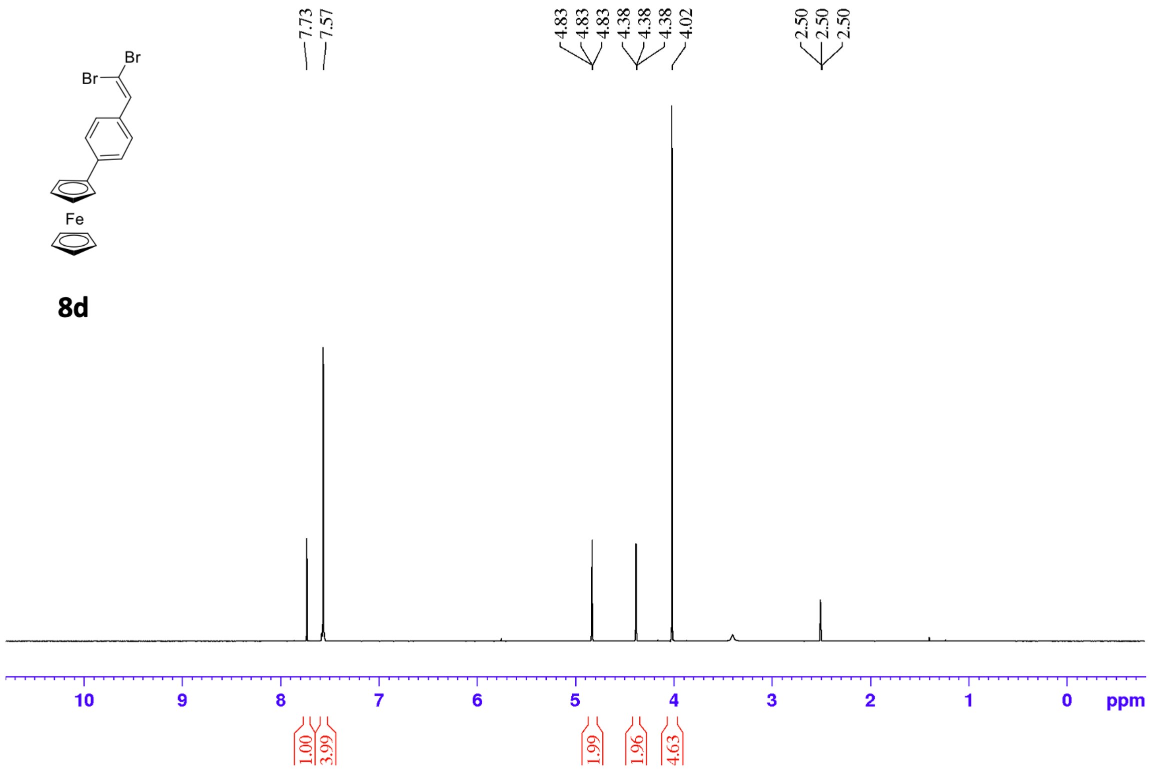

Figure S30. ^{1}H NMR spectrum of **8d** in DMSO-d_6

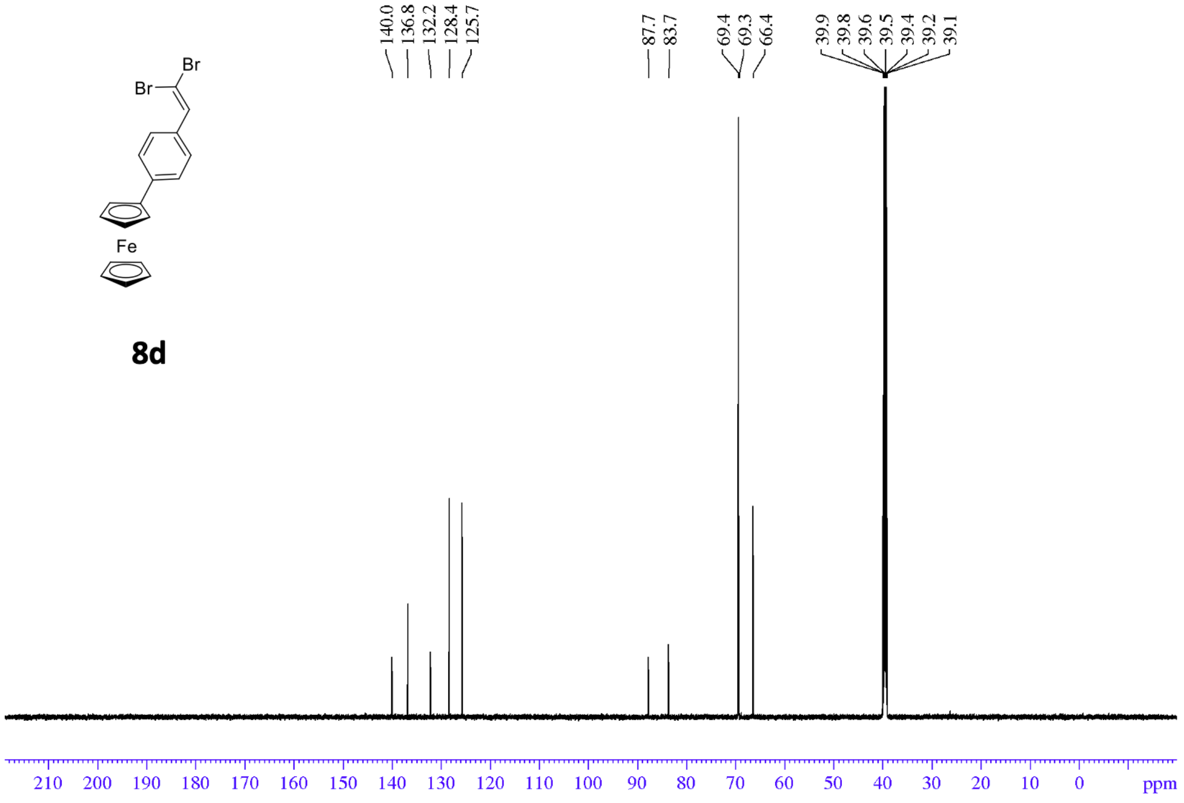

Figure S31. $^{13}C\{^{1}H\}$ NMR spectrum of **8d** in DMSO-d_6

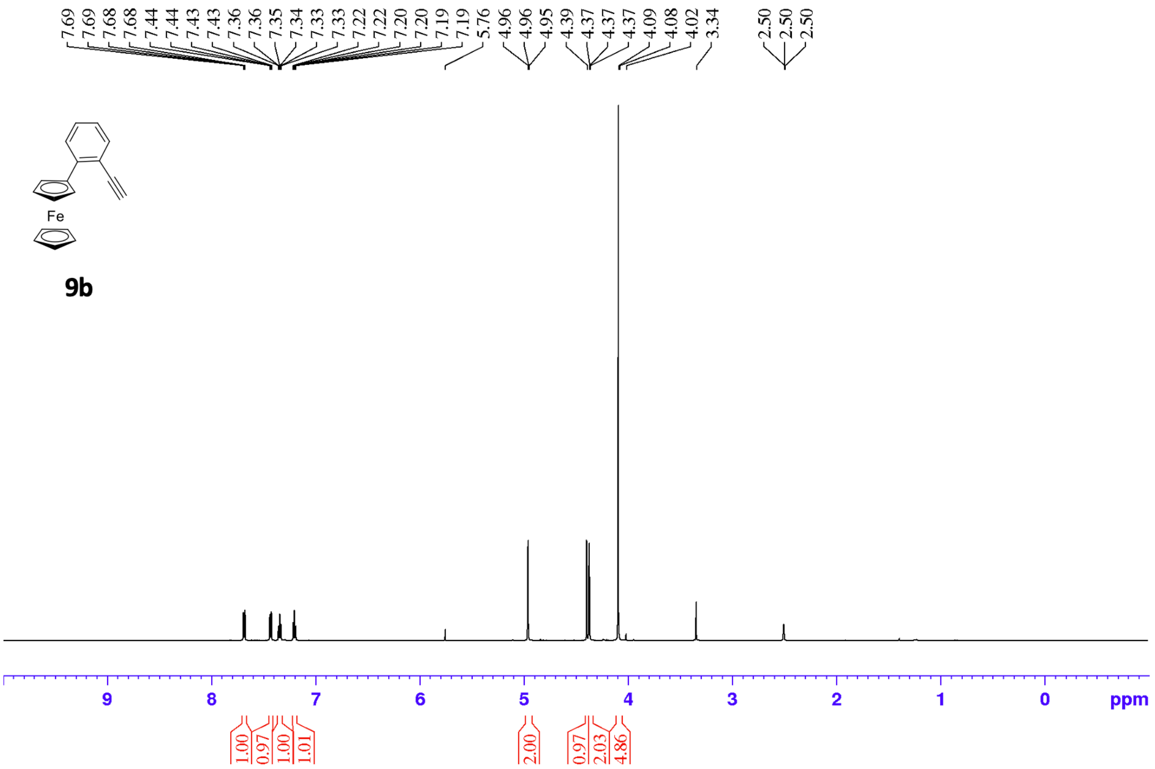

Figure S32. ^{1}H NMR spectrum of **9b** in DMSO-d_6

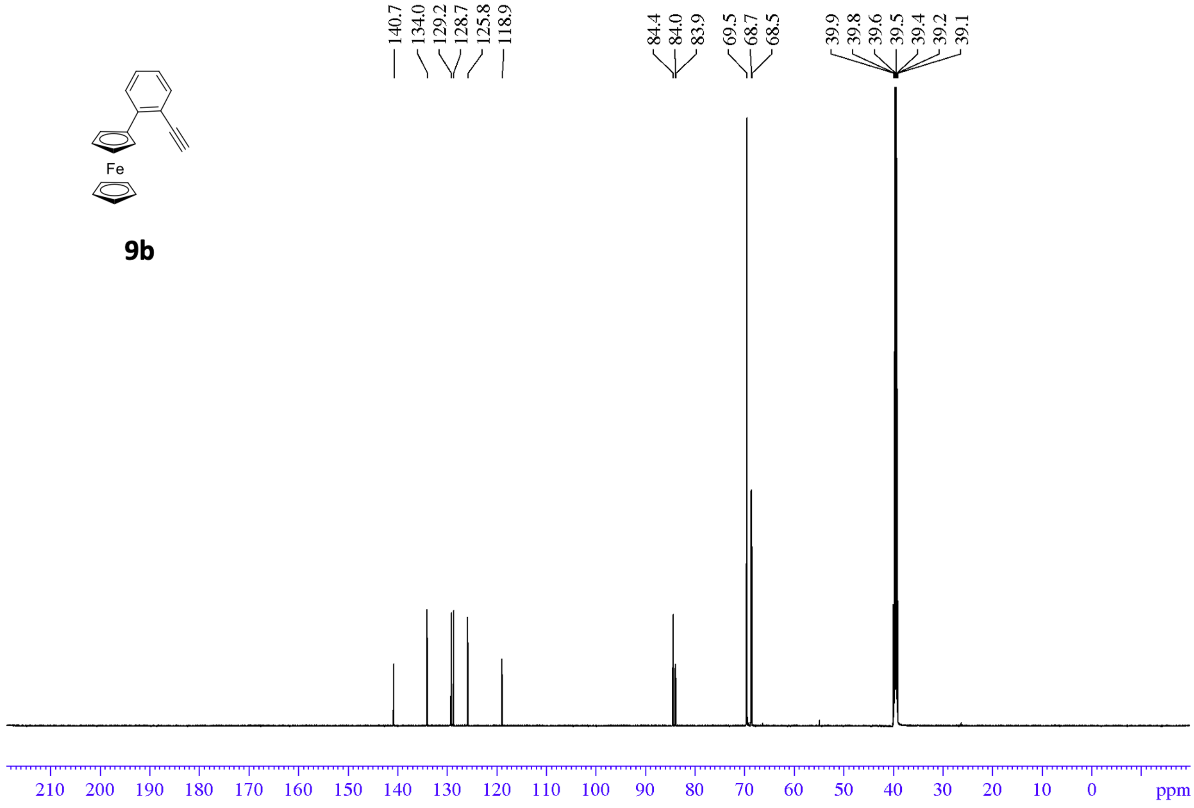

Figure S33. ^{13}C{^{1}H} NMR spectrum of **9b** in DMSO-d_6

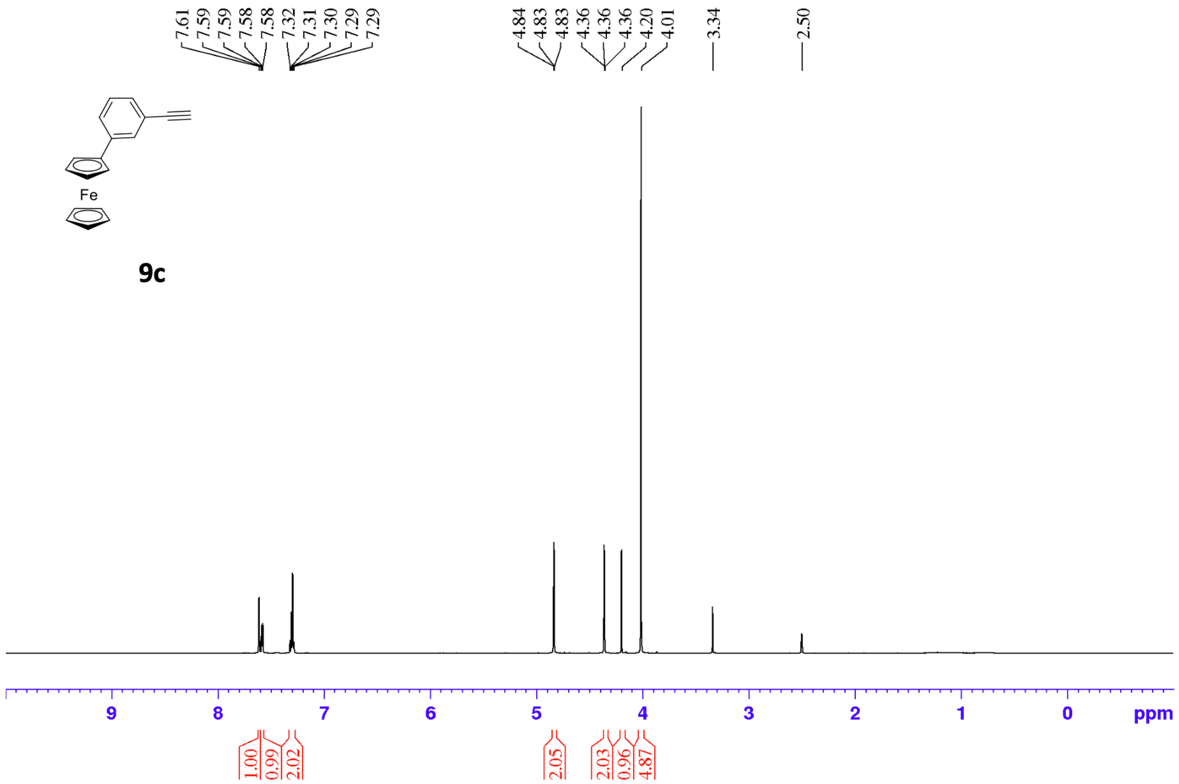

Figure S34. ^{1}H NMR spectrum of **9c** in DMSO-d_6

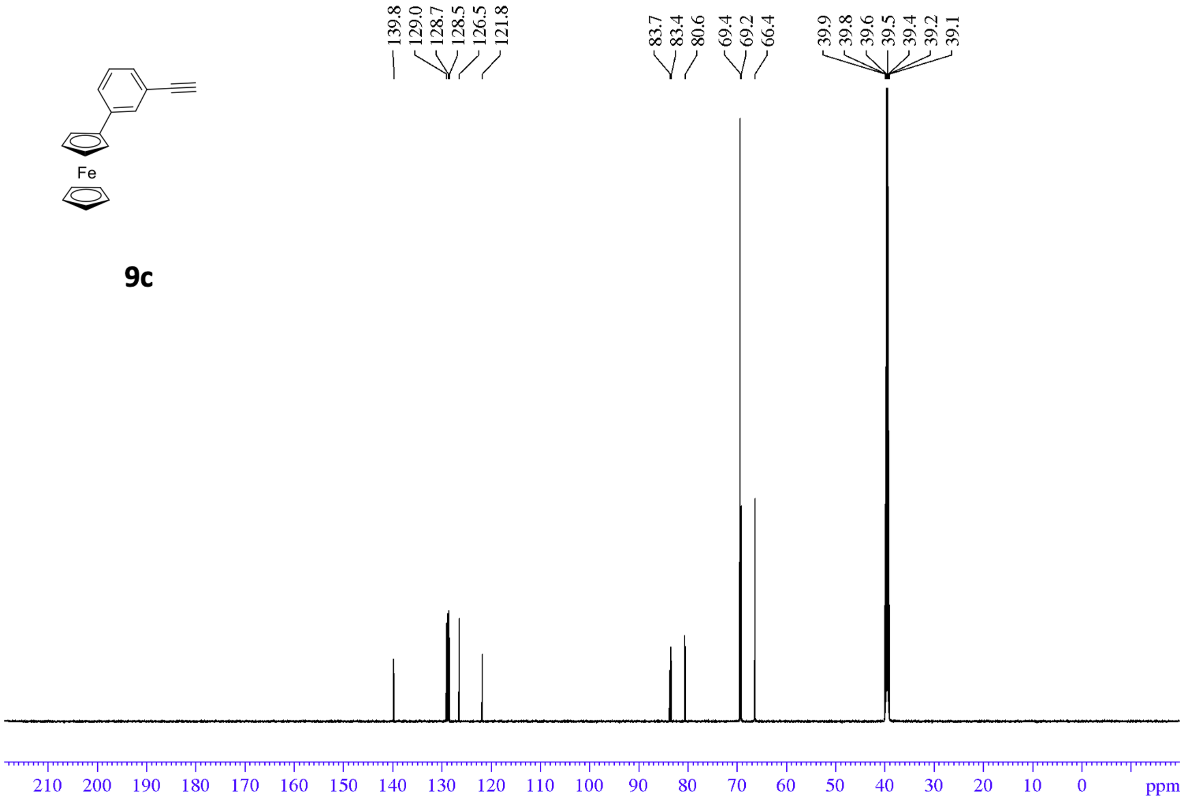

Figure S35. $^{13}C\{^{1}H\}$ NMR spectrum of **9c** in DMSO-d_6

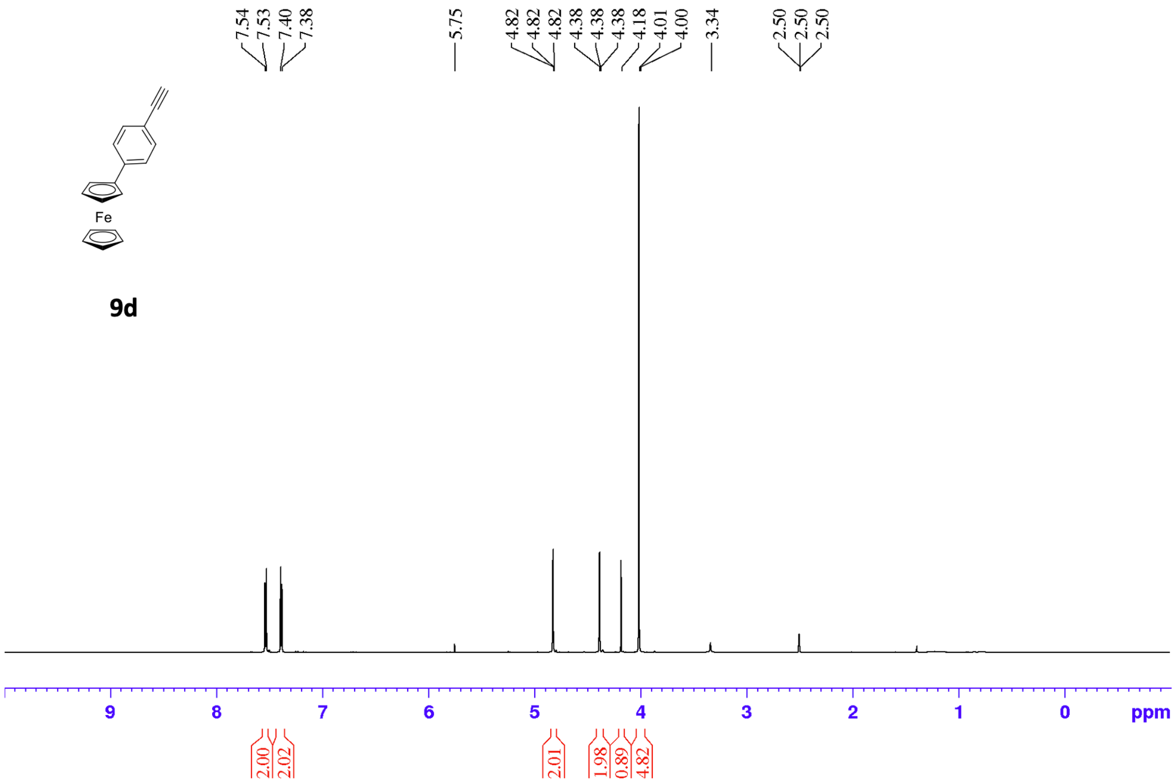

Figure S36. ^{1}H NMR spectrum of **9d** in DMSO-d_6

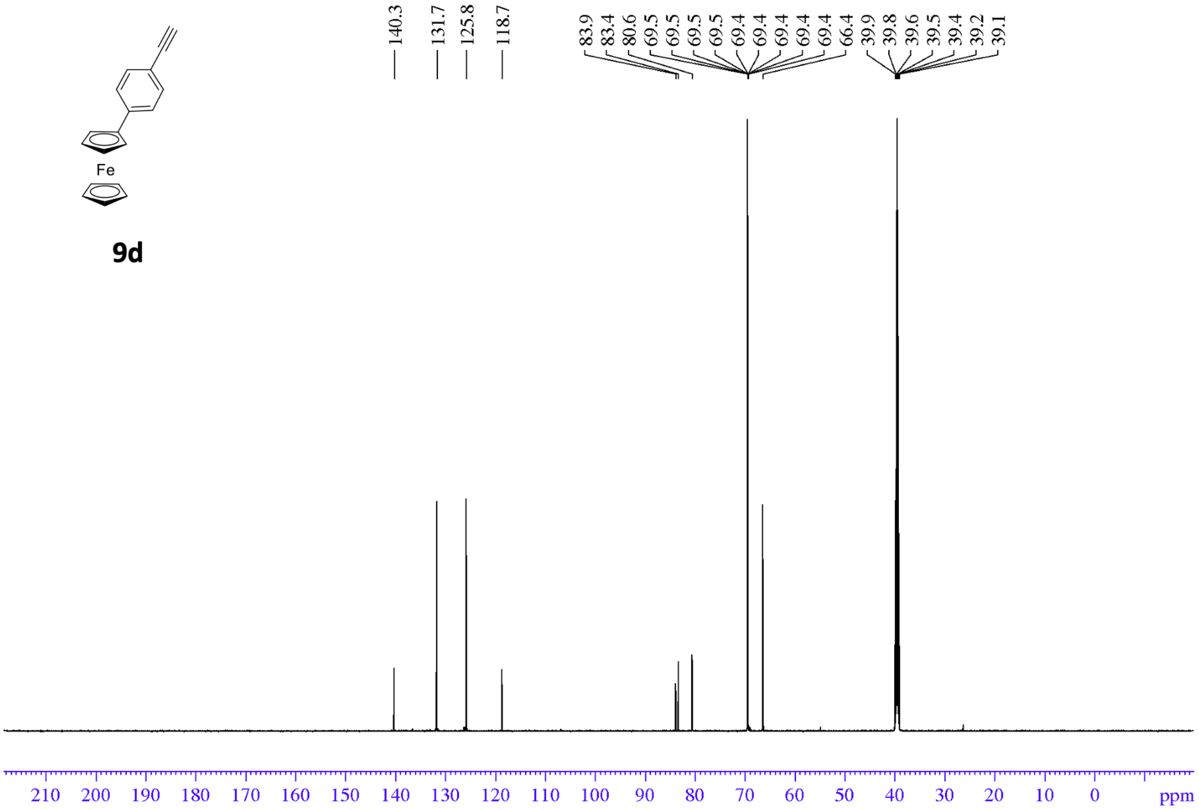

Figure S37. $^{13}C\{^{1}H\}$ NMR spectrum of **9d** in DMSO-d_6

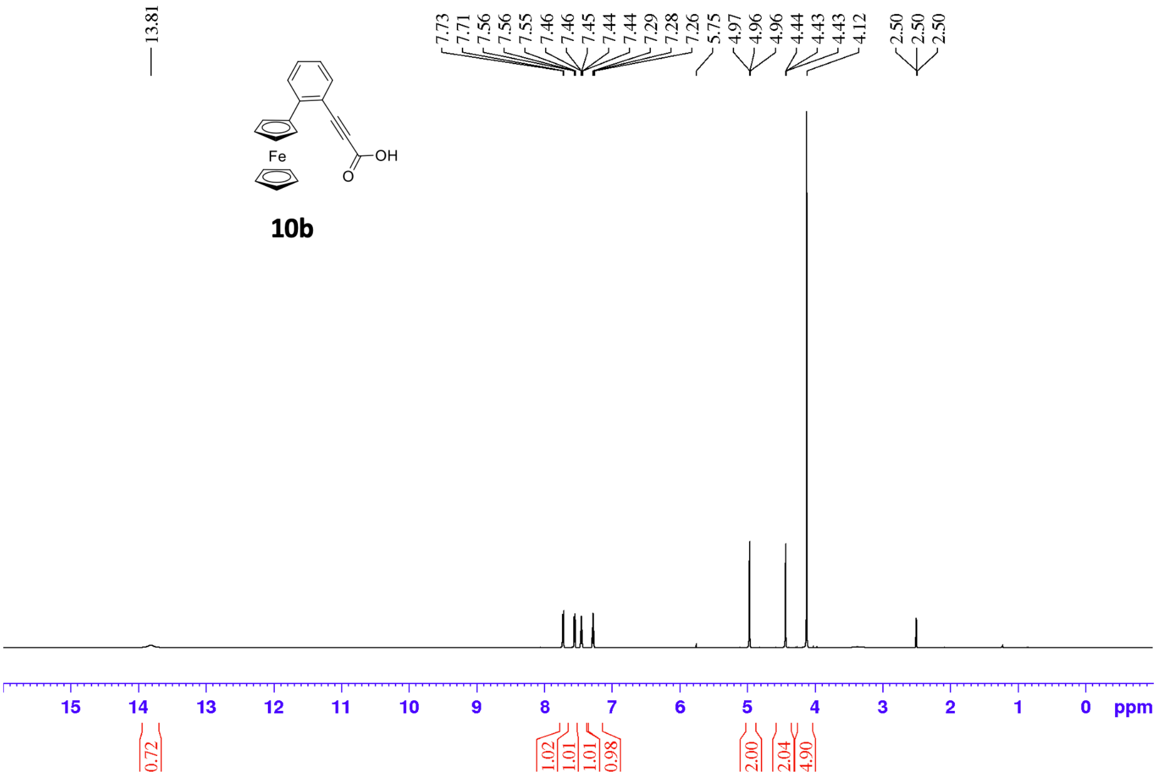

Figure S38. ^{1}H NMR spectrum of **10b** in DMSO-d_6

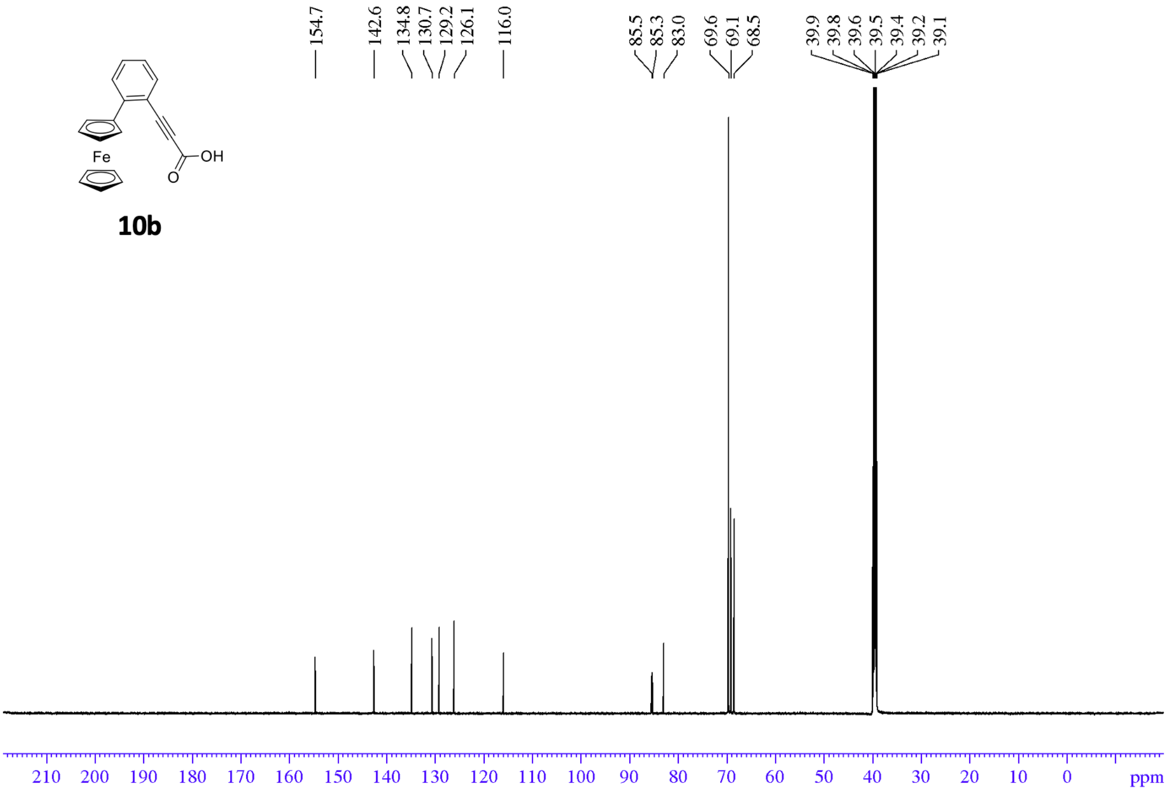

Figure S39. $^{13}C\{^{1}H\}$ NMR spectrum of **10b** in DMSO-d_6

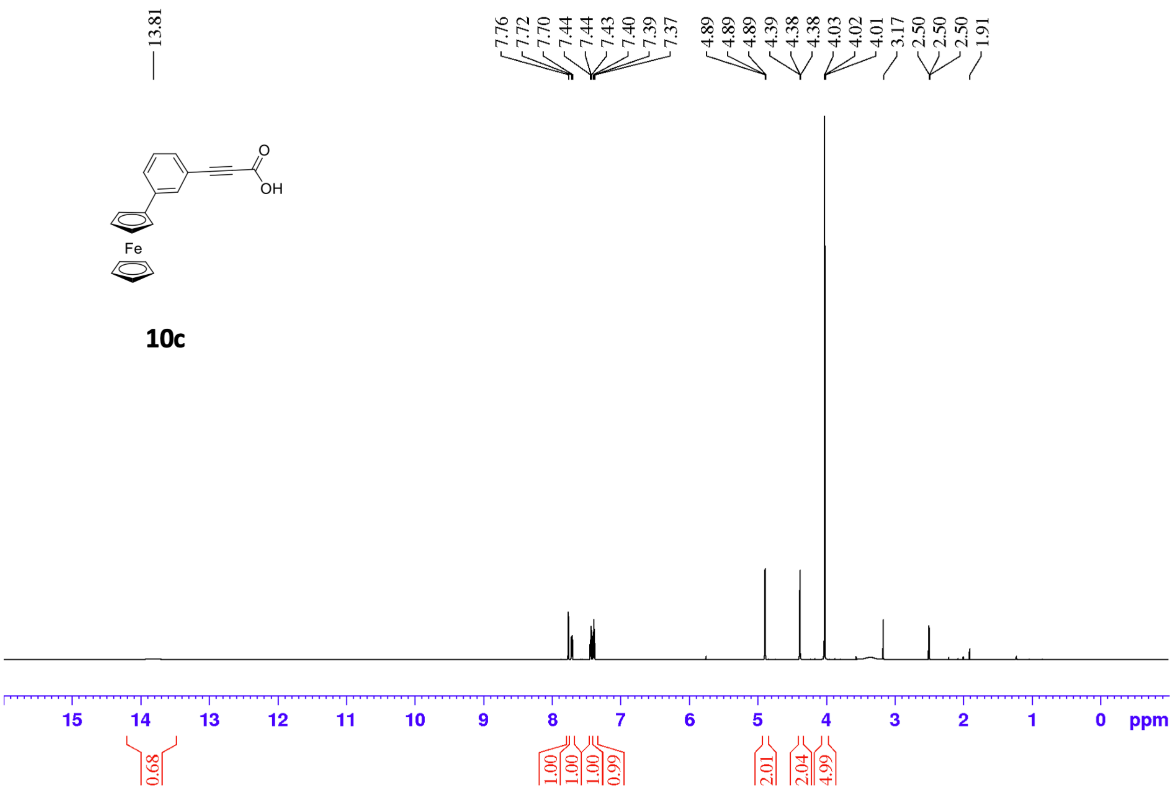

Figure S40. ^{1}H NMR spectrum of **10c** in DMSO-d_6

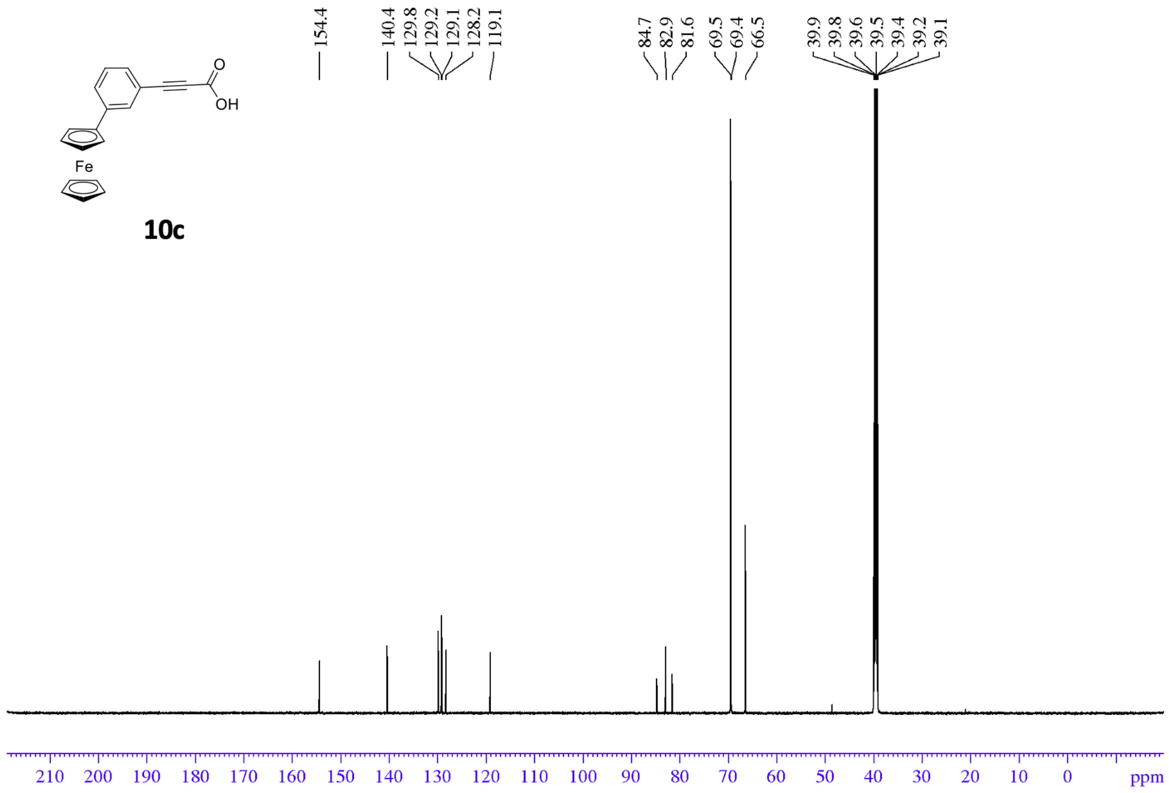

Figure S41. ^{13}C{^{1}H} NMR spectrum of **10c** in DMSO-d_6

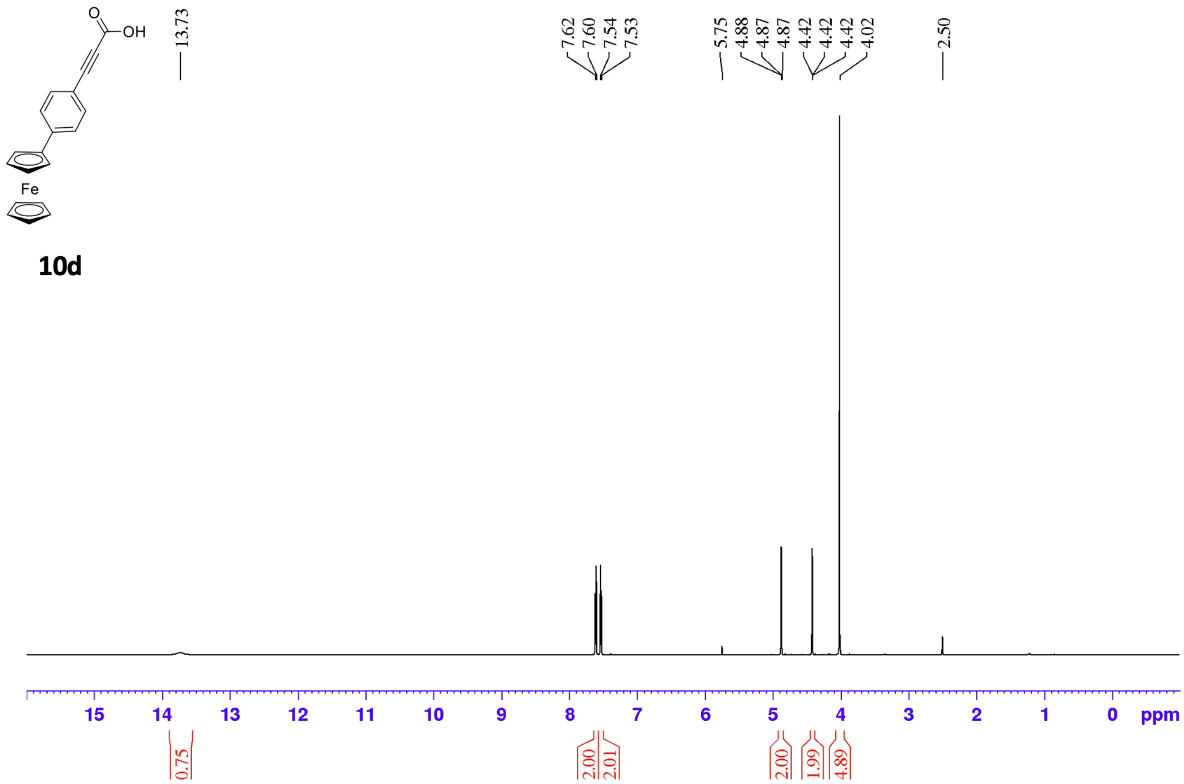

Figure S42. ^{1}H NMR spectrum of **10d** in DMSO-d$_6$

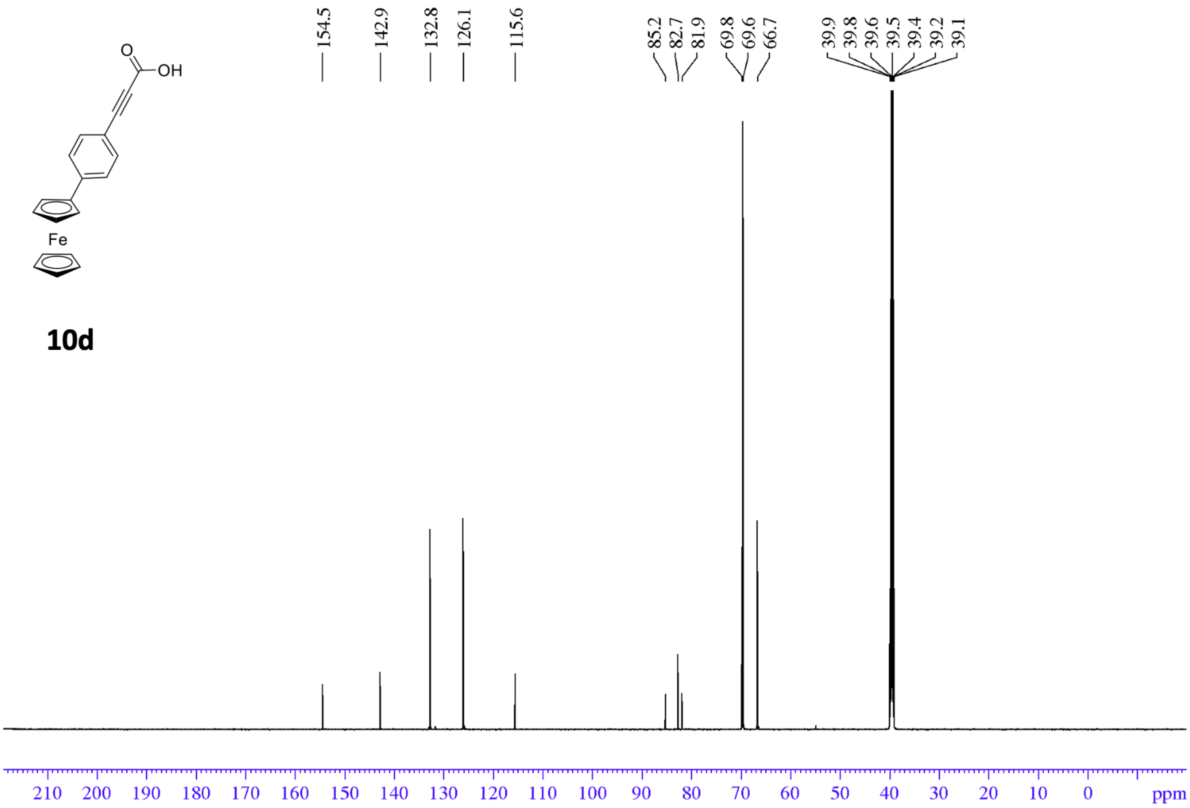

Figure S43. ^{13}C{^{1}H} NMR spectrum of **10d** in DMSO-d$_6$

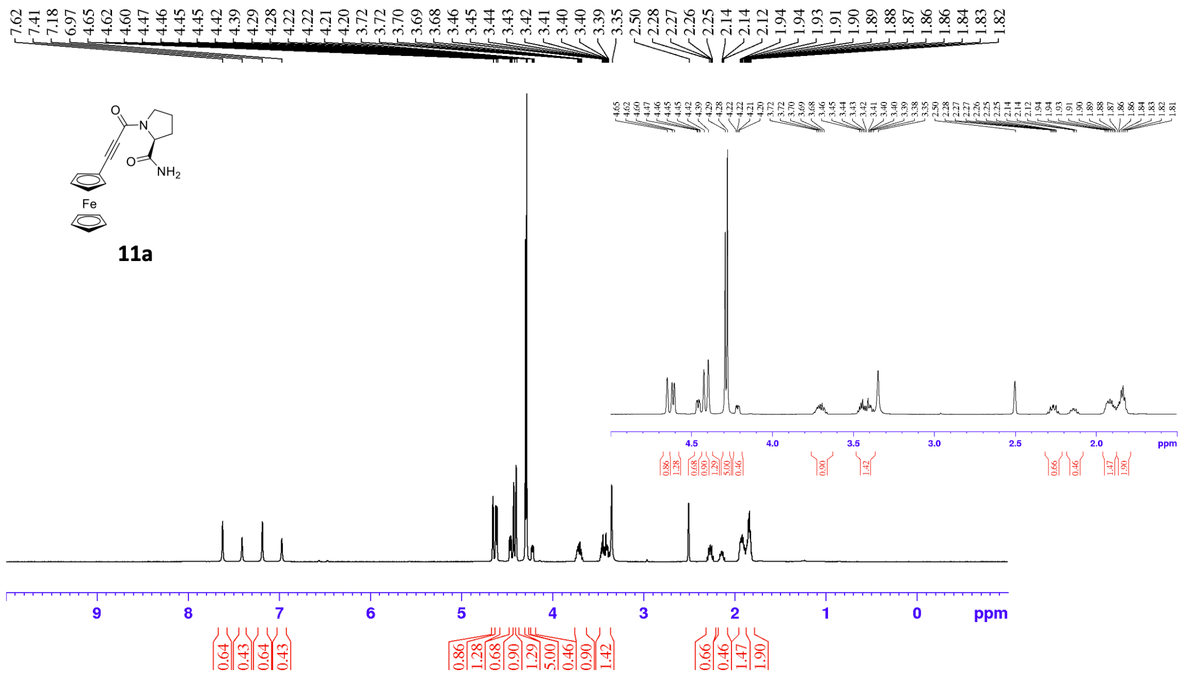

Figure S44. ^{1}H NMR spectrum of **11a** in DMSO-d$_6$

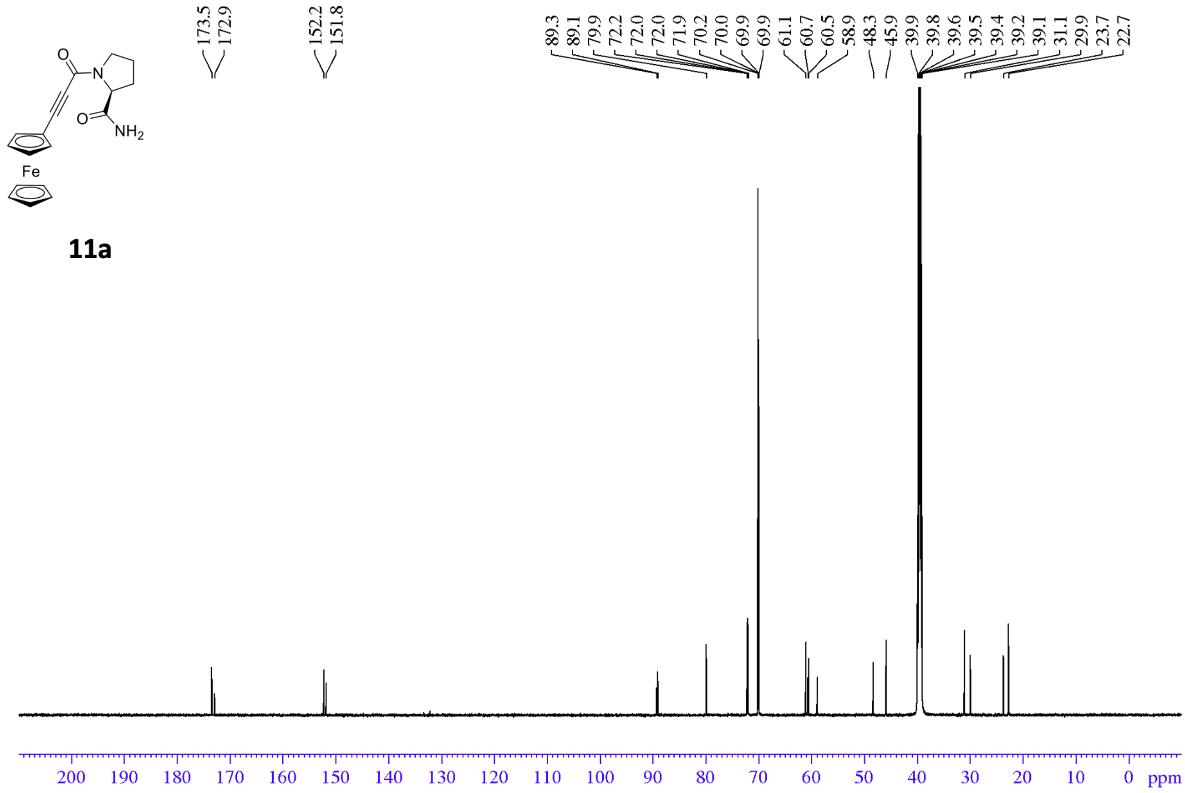

Figure S45. $^{13}C\{^{1}H\}$ NMR spectrum of **11a** in DMSO-d$_6$

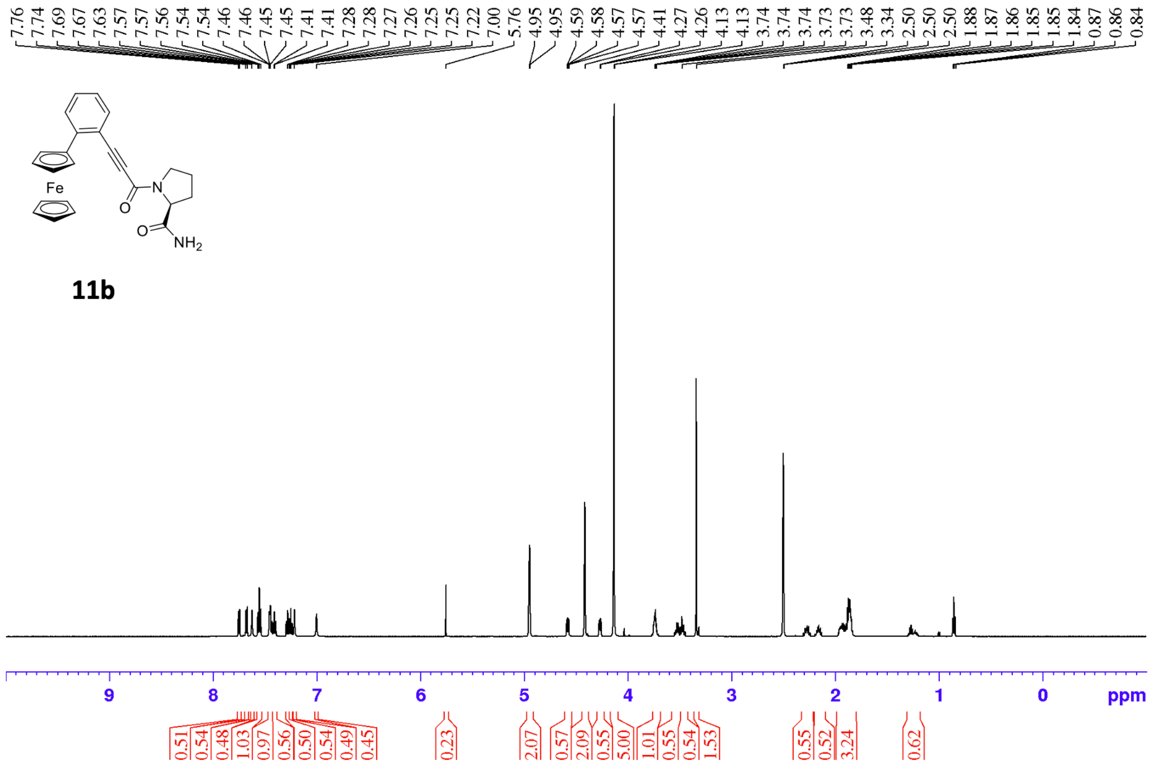

Figure S46. ^{1}H NMR spectrum of **11b** in DMSO-d_6

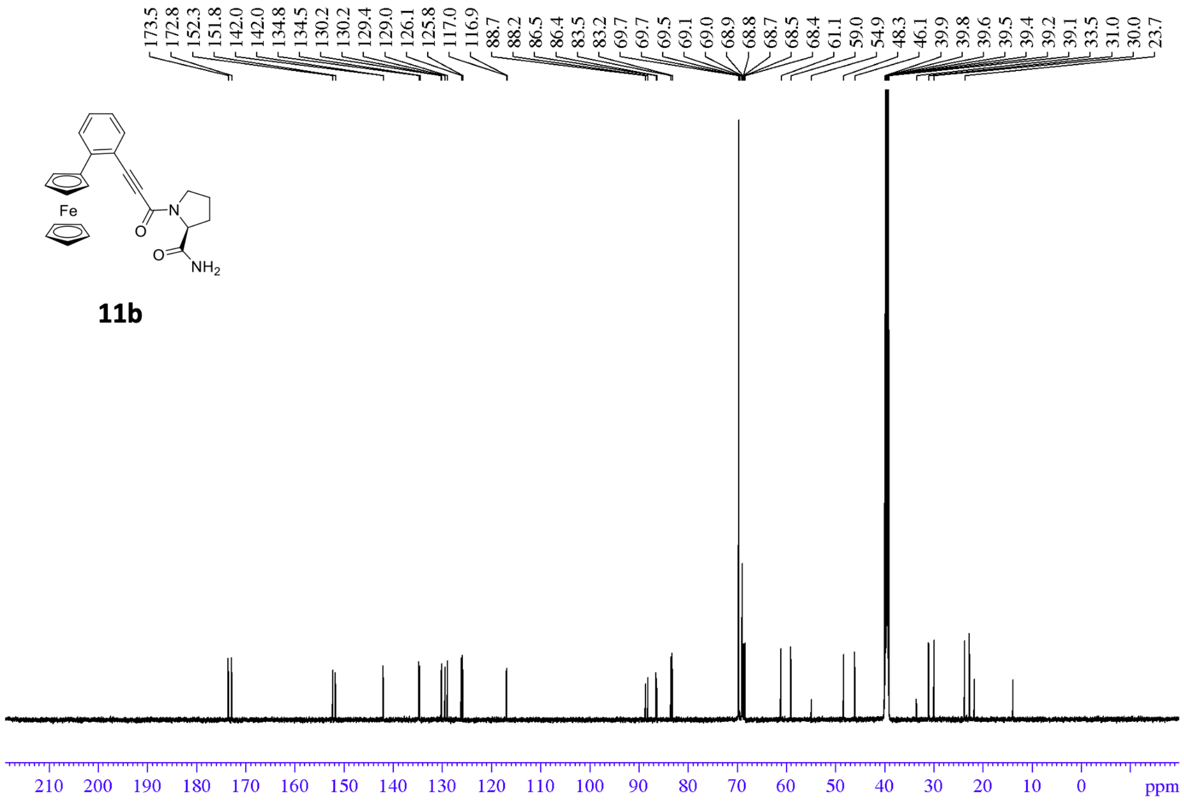

Figure S47. $^{13}C\{^{1}H\}$ NMR spectrum of **11b** in DMSO-d_6

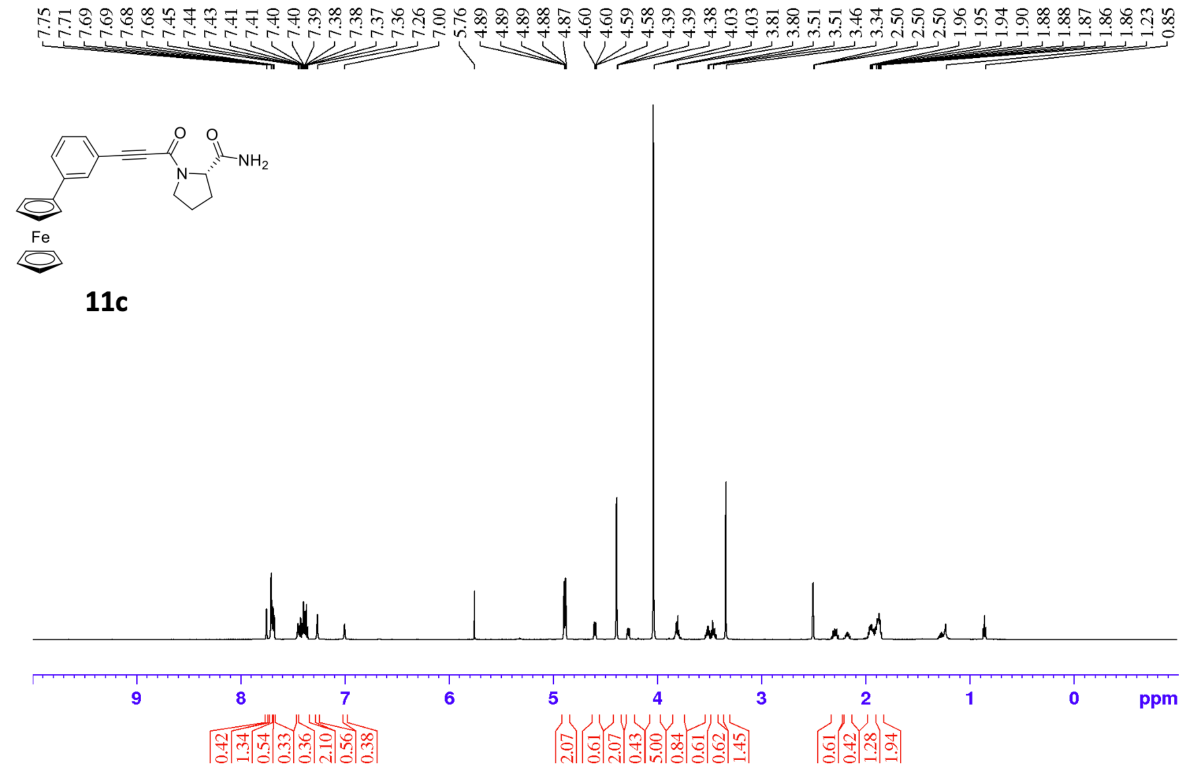

Figure S48. ^{1}H NMR spectrum of **11c** in DMSO-d6

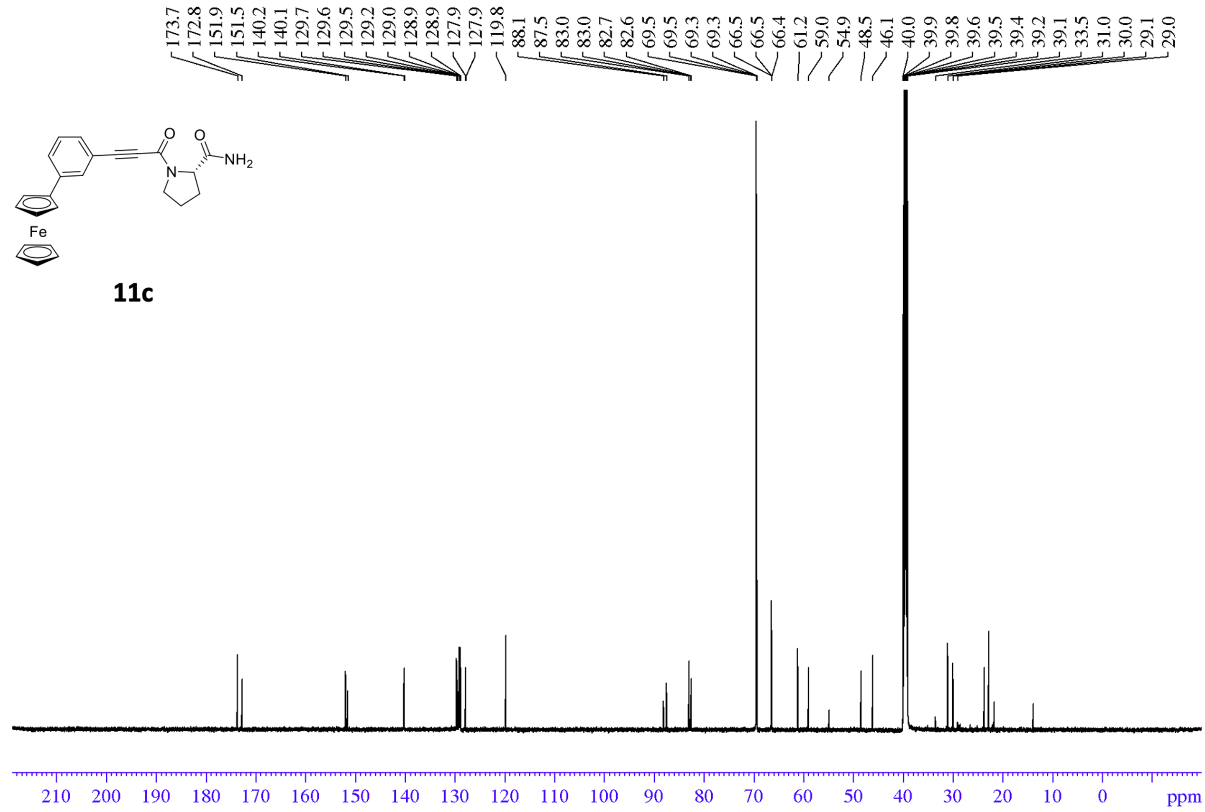

Figure S49. $^{13}C\{^{1}H\}$ NMR spectrum of **11c** in DMSO-d6

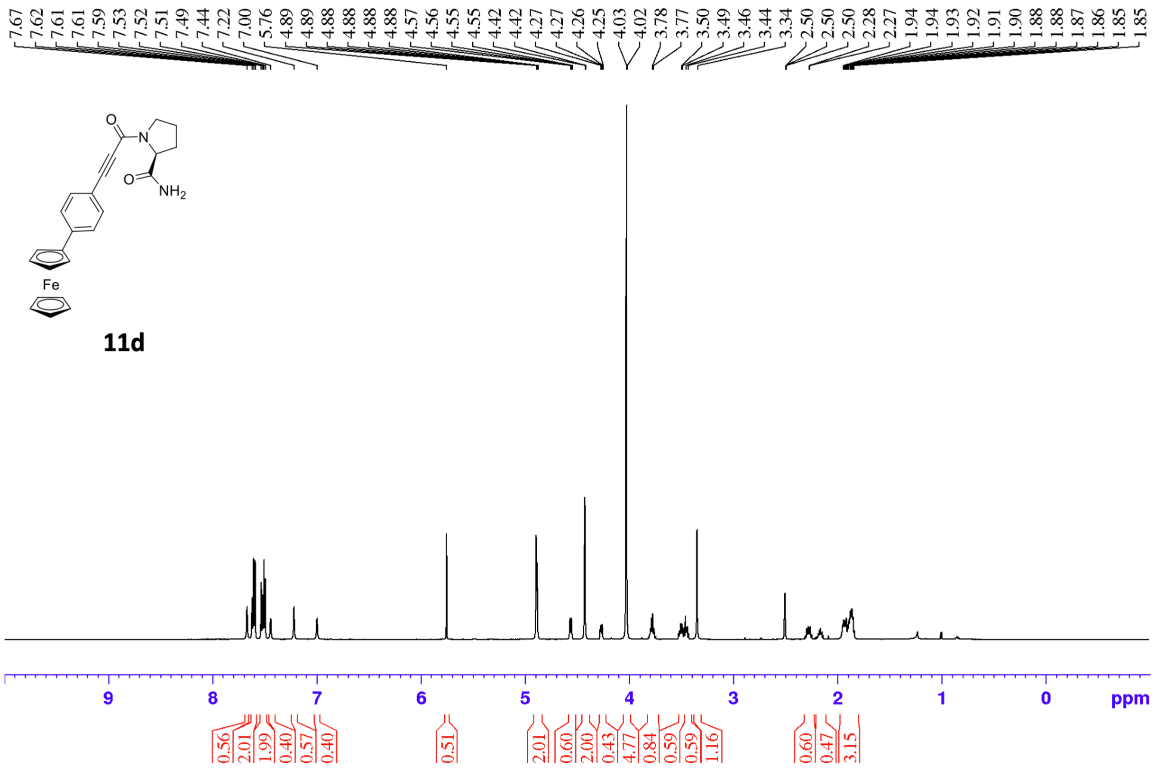

Figure S50. ^{1}H NMR spectrum of **11d** in DMSO-d$_6$

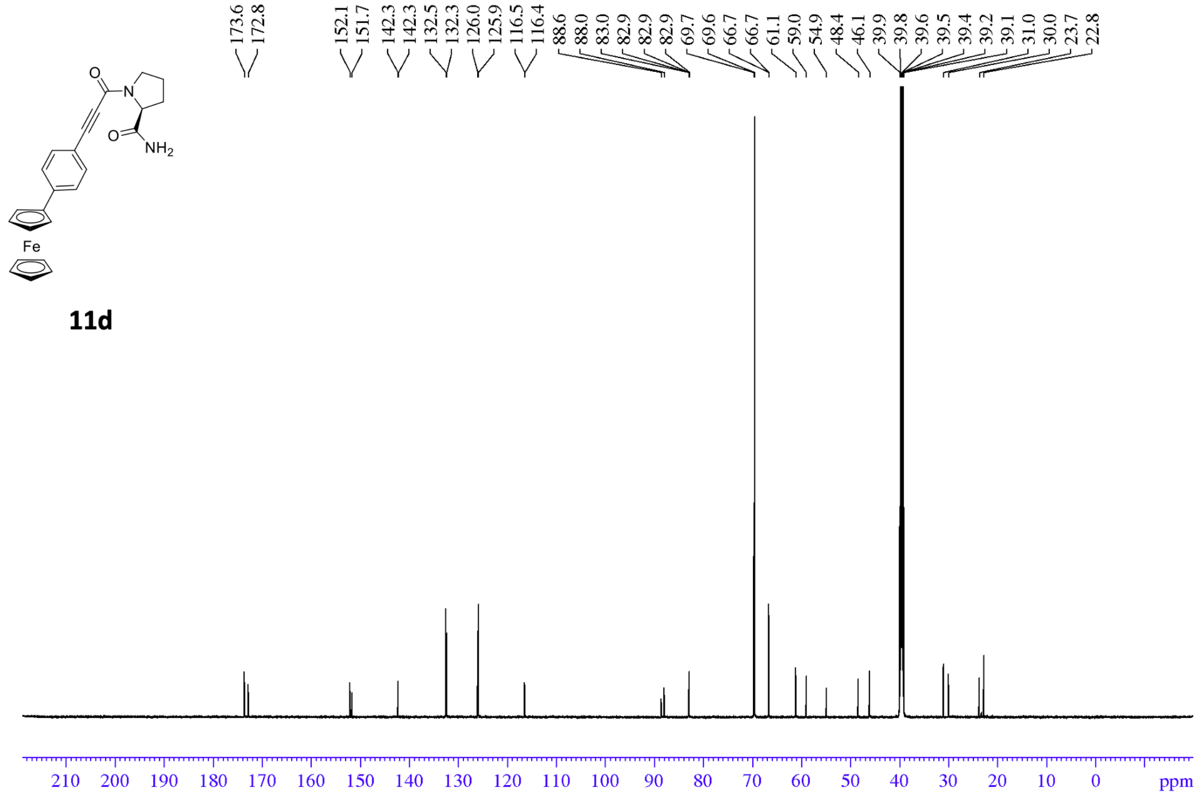

Figure S51. $^{13}C\{^{1}H\}$ NMR spectrum of **11d** in DMSO-d$_6$

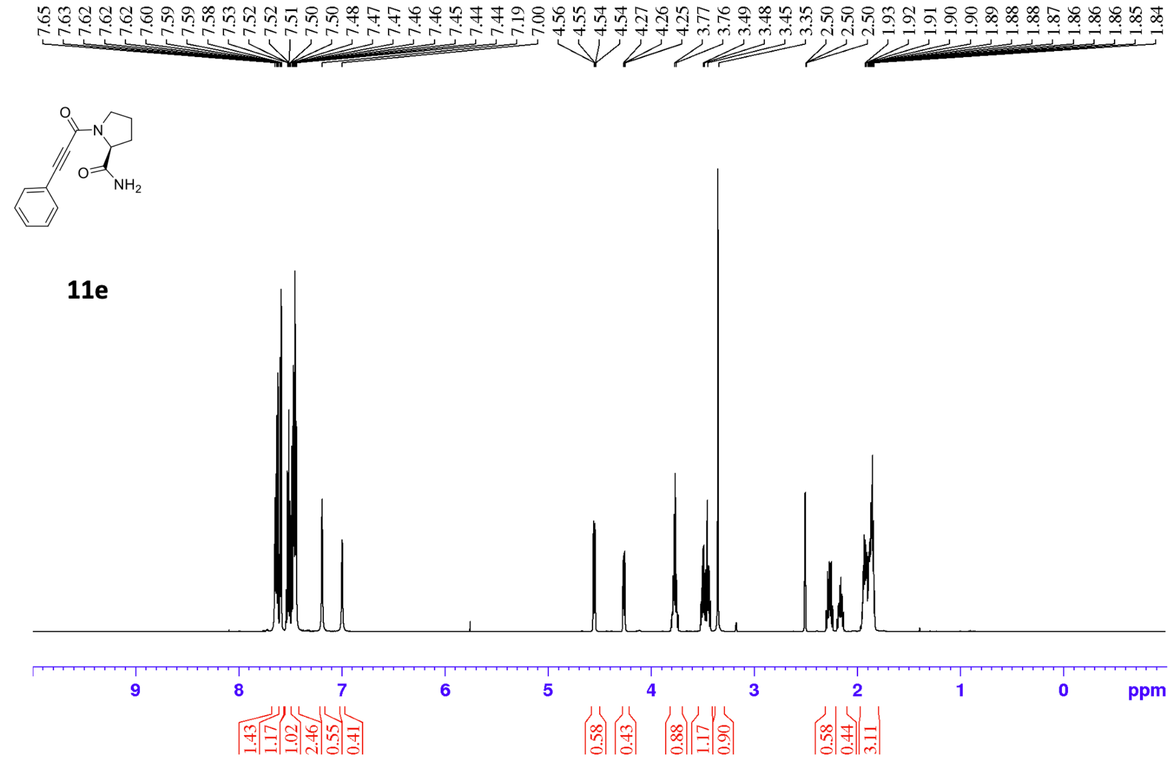

Figure S52. ^{1}H NMR spectrum of **11e** in DMSO-d6

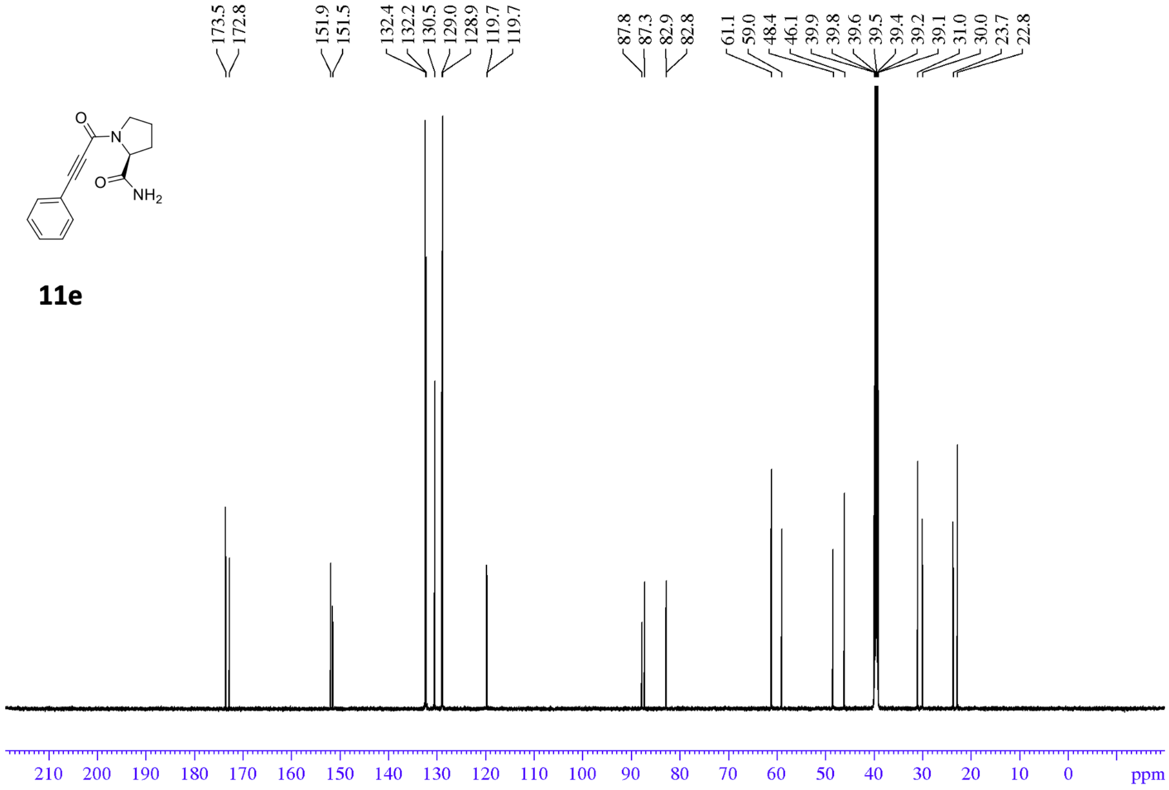

Figure S53. $^{13}C\{^{1}H\}$ NMR spectrum of **11e** in DMSO-d6

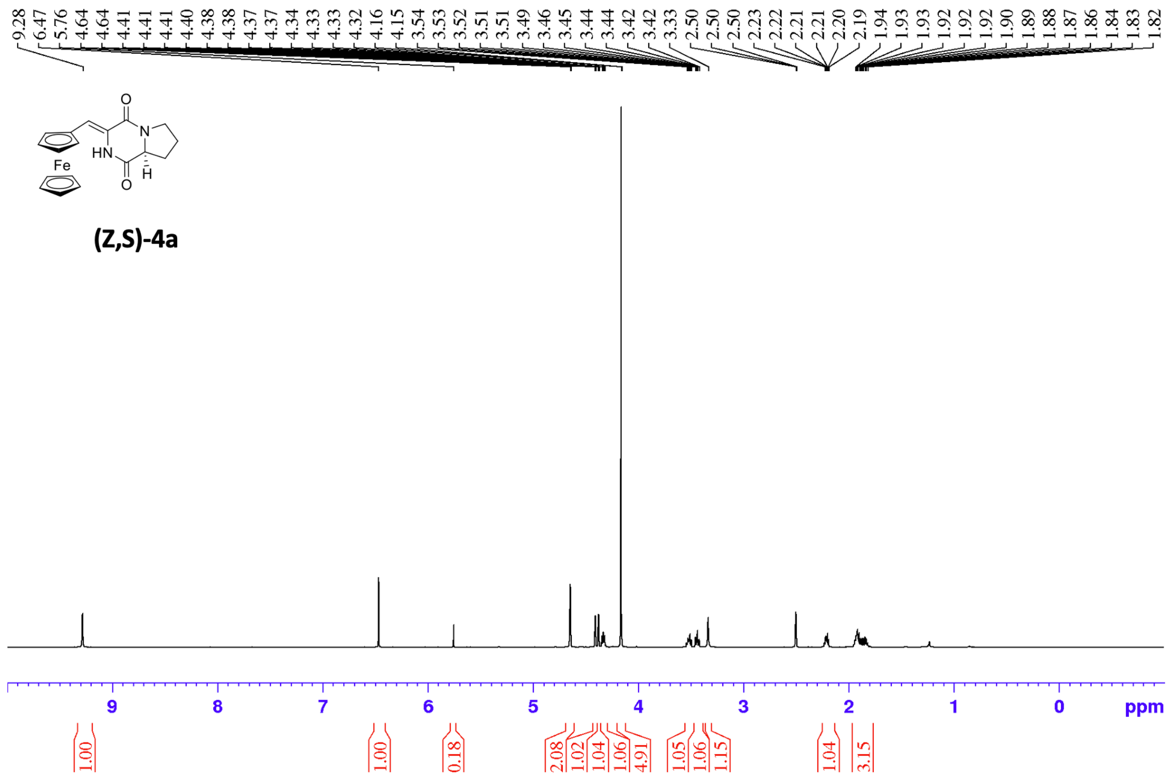

Figure S54. ^{1}H NMR spectrum of (*S,Z*)-**4a** in DMSO-d_6

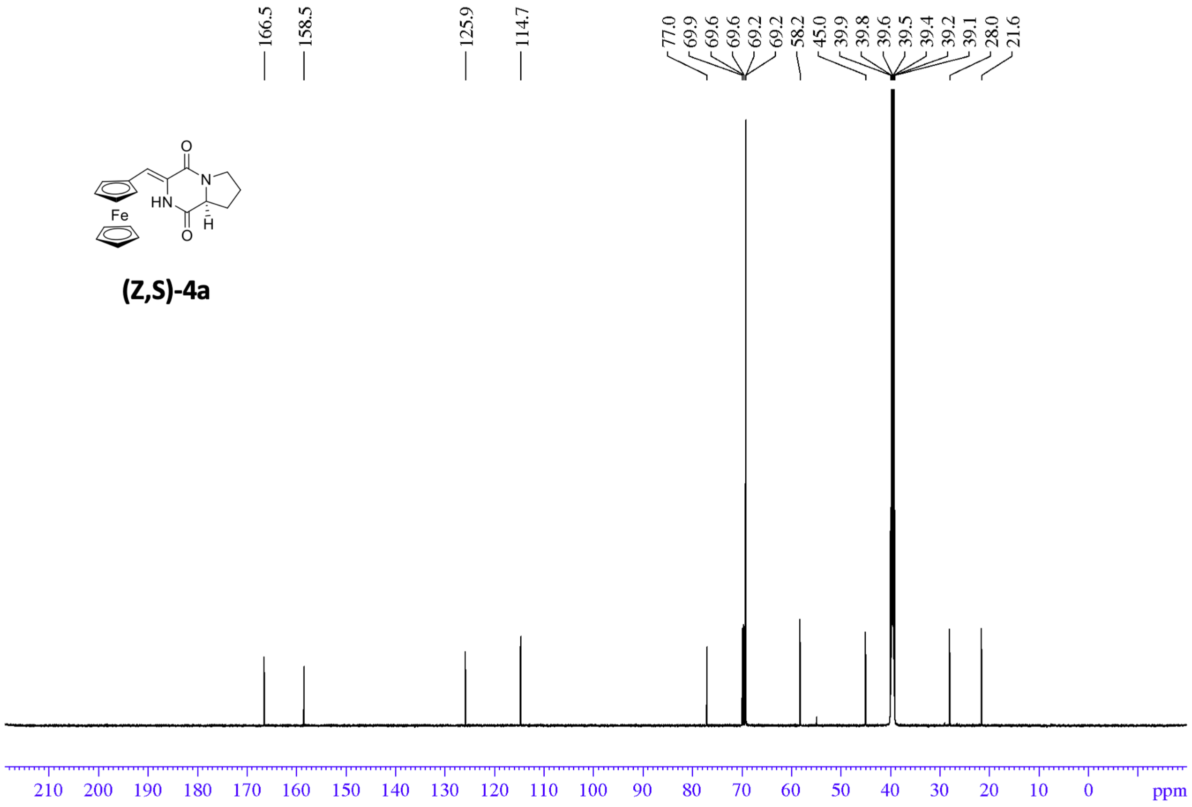

Figure S55. ^{13}C{^{1}H} NMR spectrum of (*S,Z*)-**4a** in DMSO-d_6

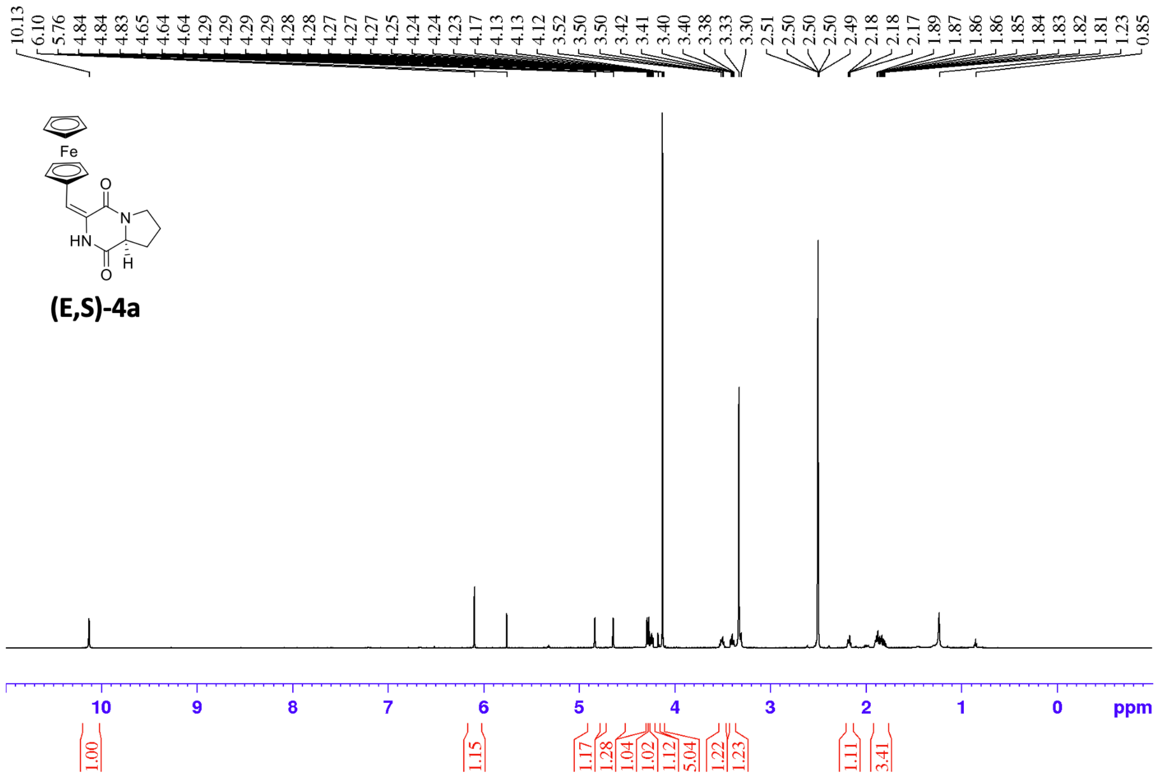

Figure S56. ^{1}H NMR spectrum of (*S,E*)-**4a** in DMSO-d6

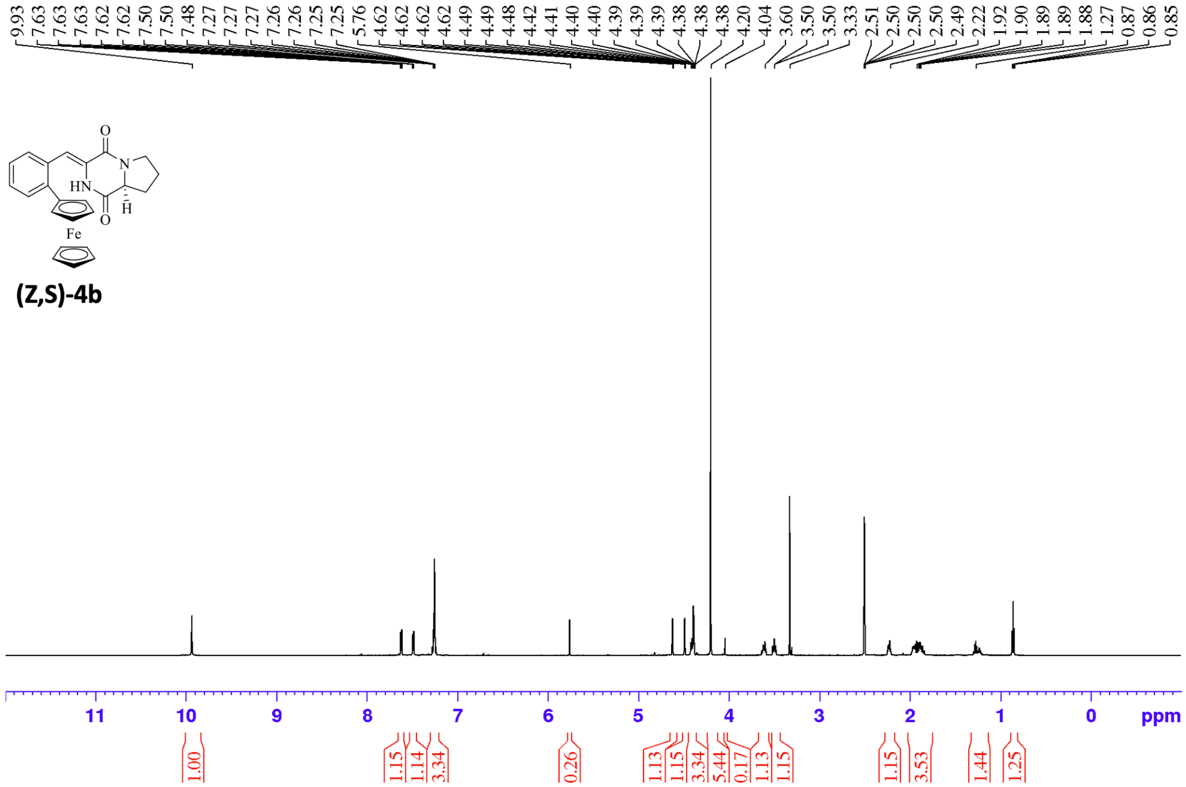

Figure S57. ^{1}H NMR spectrum of (*S,Z*)-**4b** in DMSO-d6

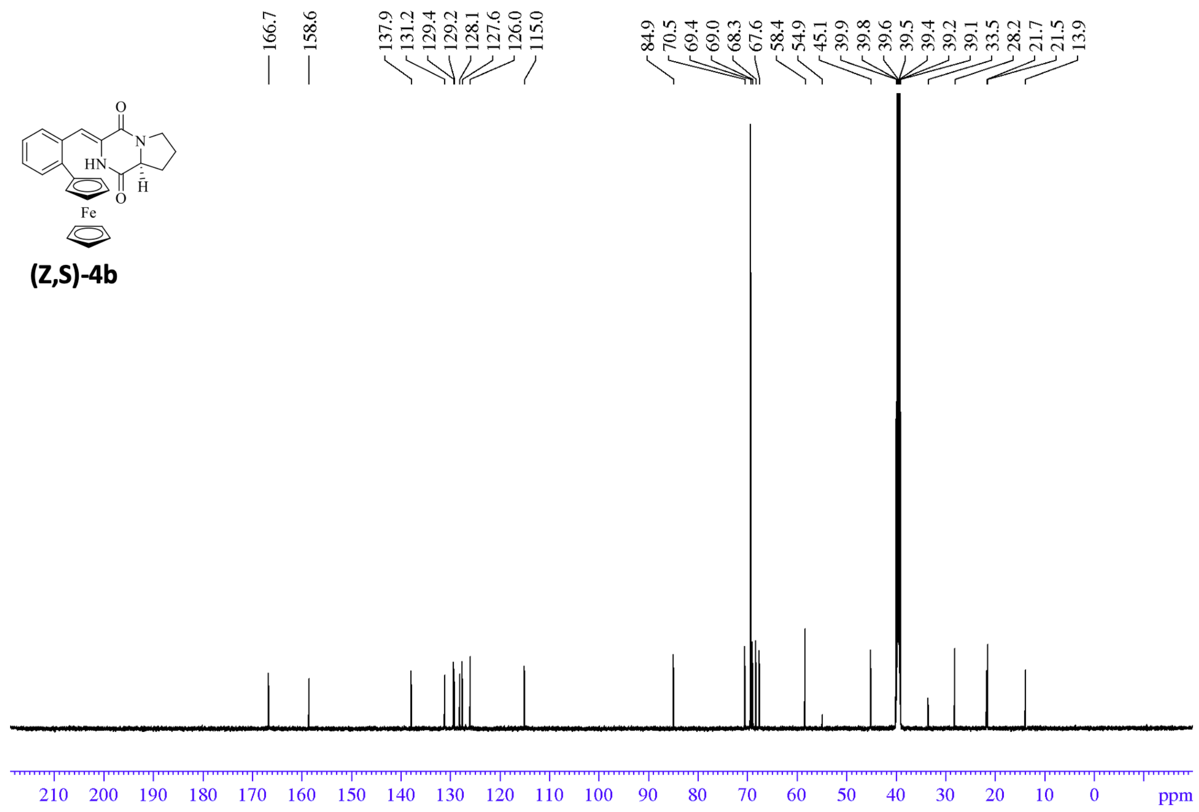

Figure S58. $^{13}C\{^{1}H\}$ NMR spectrum of (*S*,*Z*)-**4b** in DMSO-d$_6$

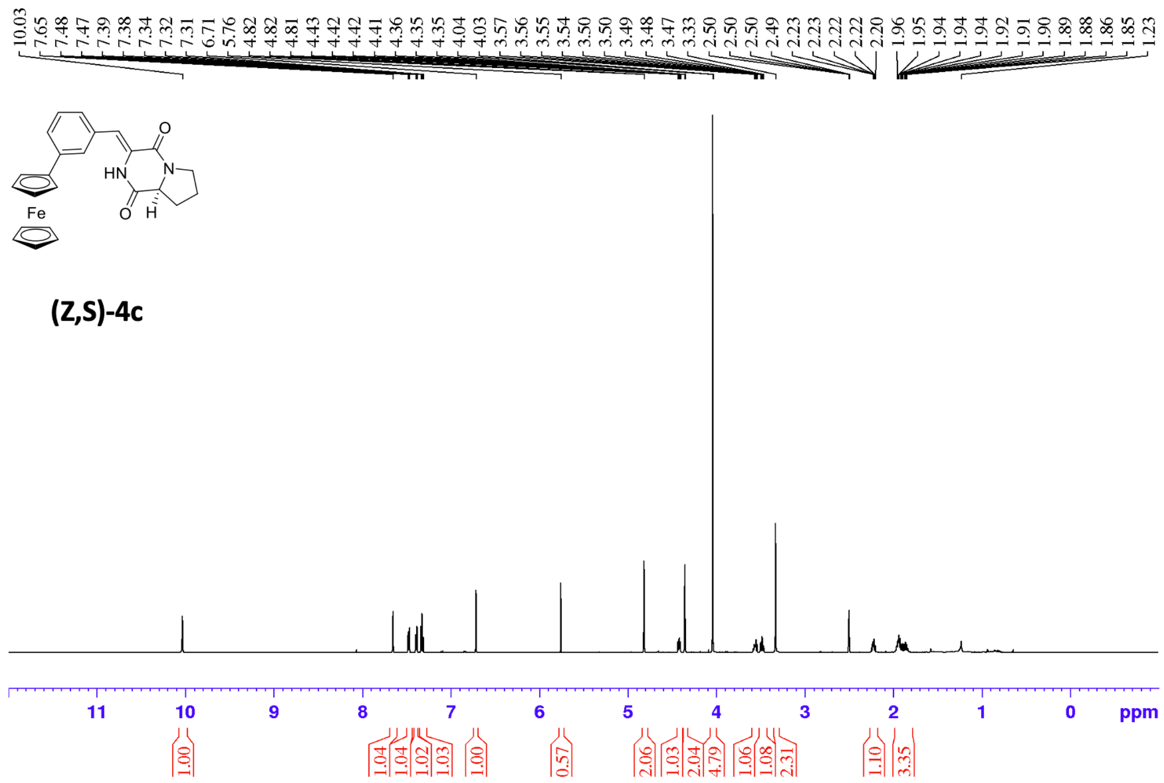

Figure S59. ^{1}H NMR spectrum of (*S*,*Z*)-**4c** in DMSO-d$_6$

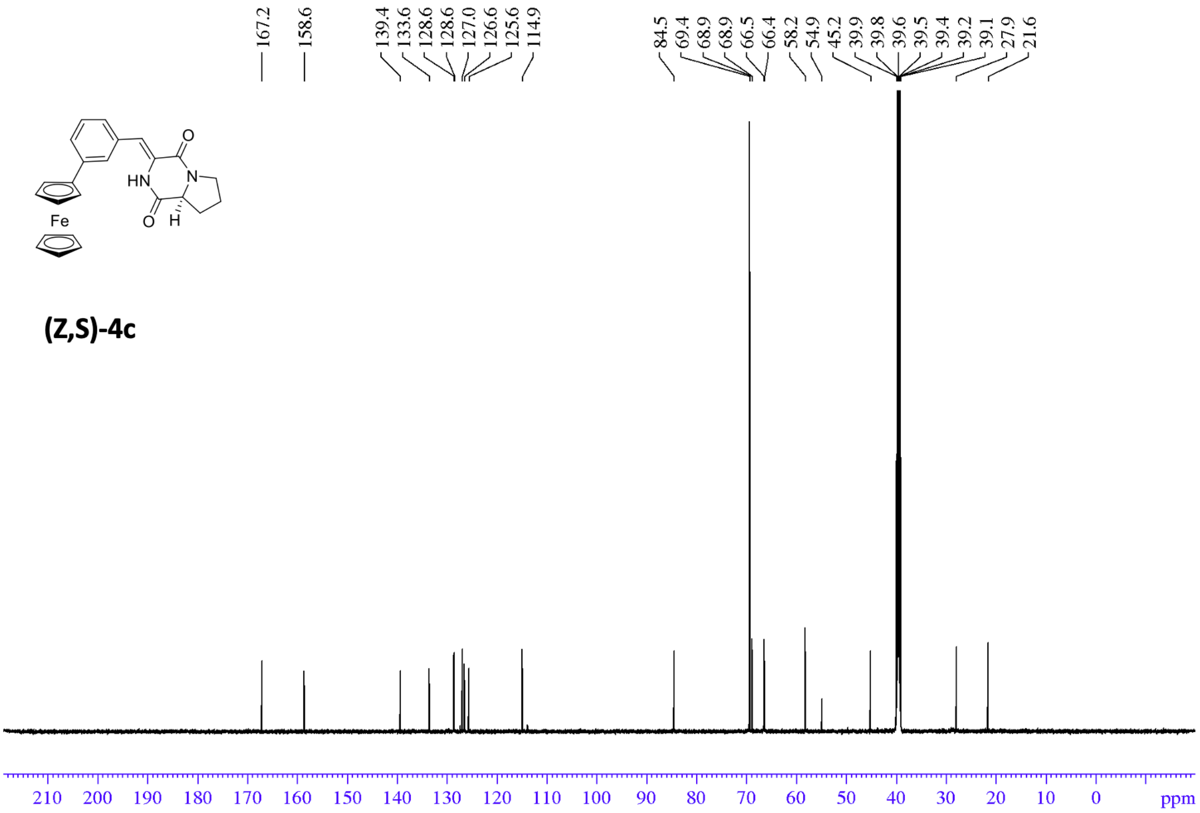

Figure S60. $^{13}C\{^{1}H\}$ NMR spectrum of (*S,Z*)-**4c** in DMSO-d_6

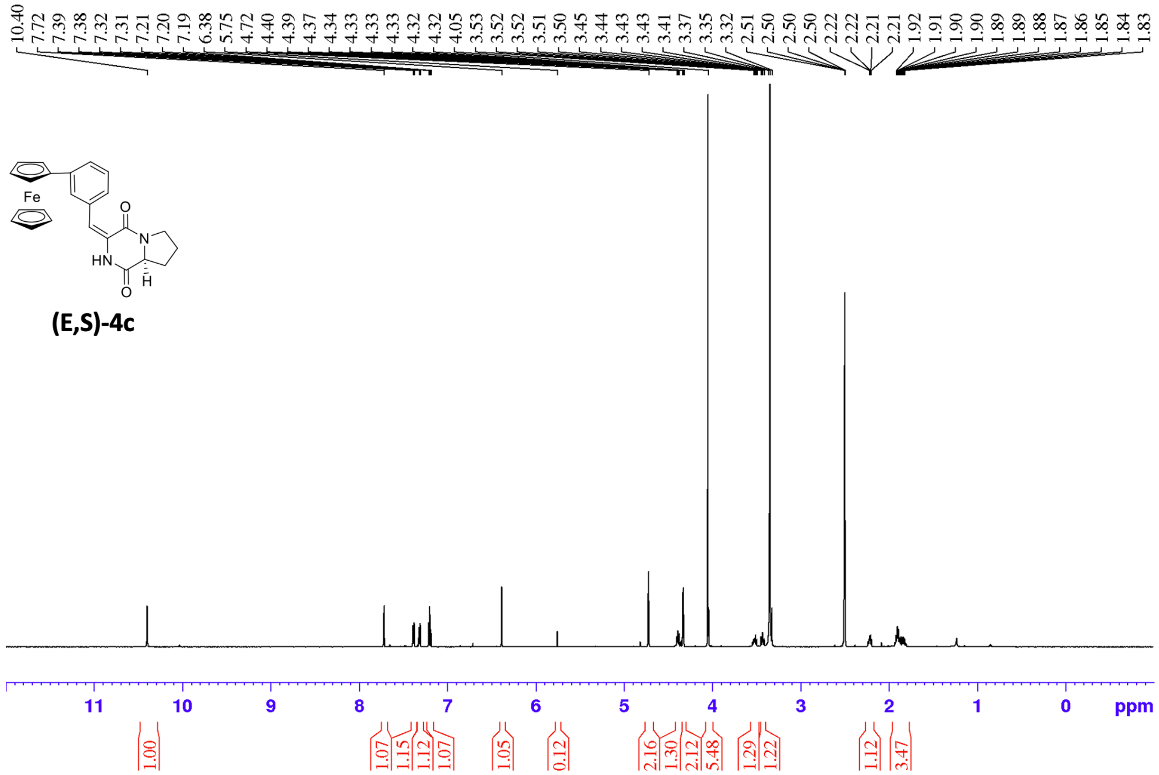

Figure S61. ^{1}H NMR spectrum of (*S,E*)-**4c** in DMSO-d_6

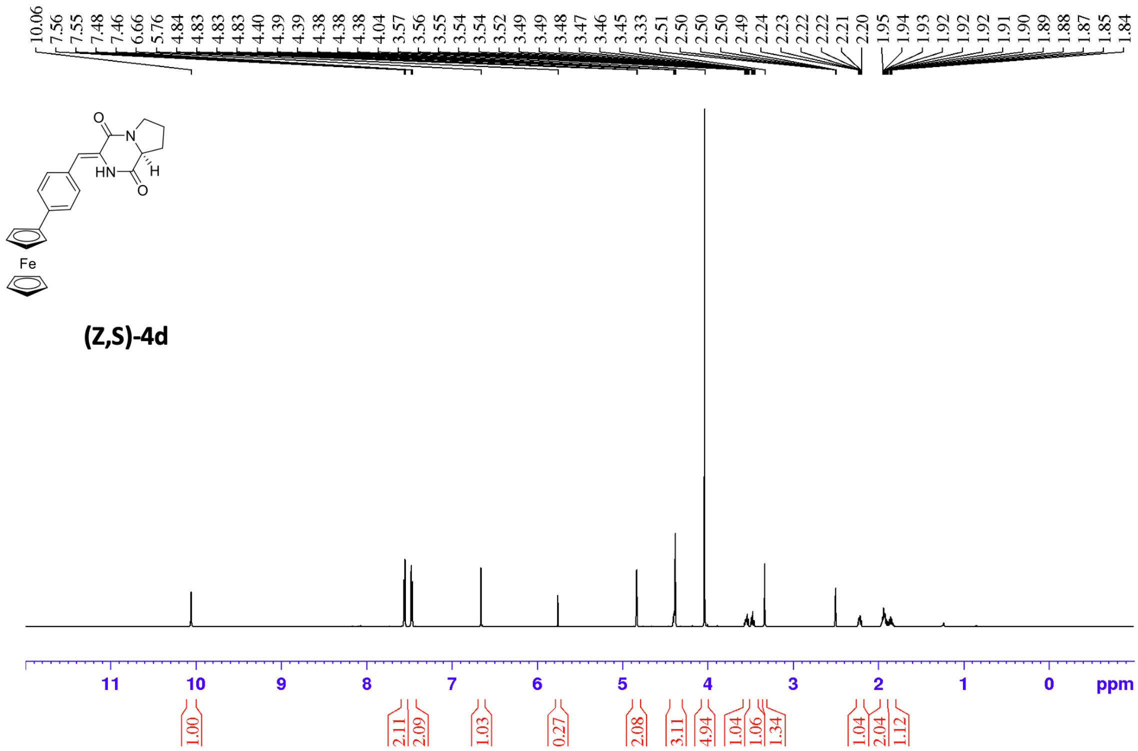

Figure S62. ^{1}H NMR spectrum of (*S,Z*)-**4d** in DMSO-d$_6$

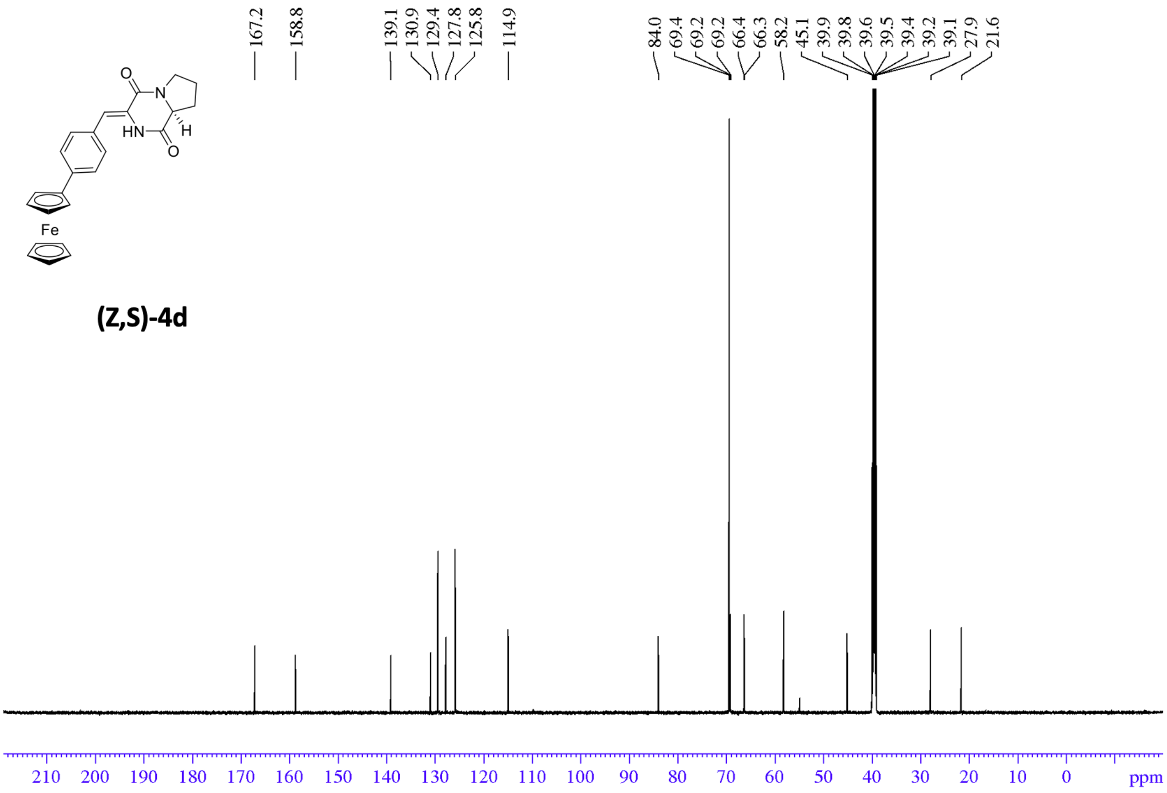

Figure S63. ^{13}C{^{1}H} NMR spectrum of ((*S,Z*)-**4d** in DMSO-d$_6$

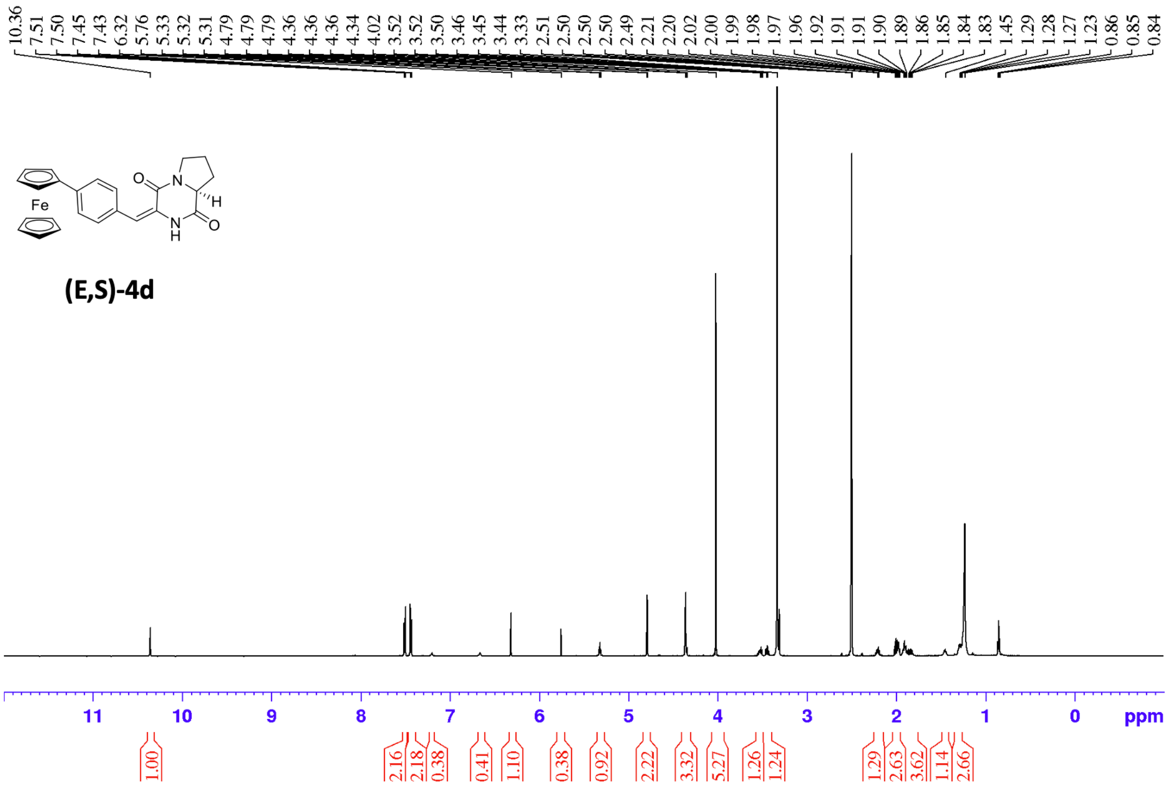

Figure S64. ^{1}H NMR spectrum of (*S,E*)-**4d** in DMSO-d6

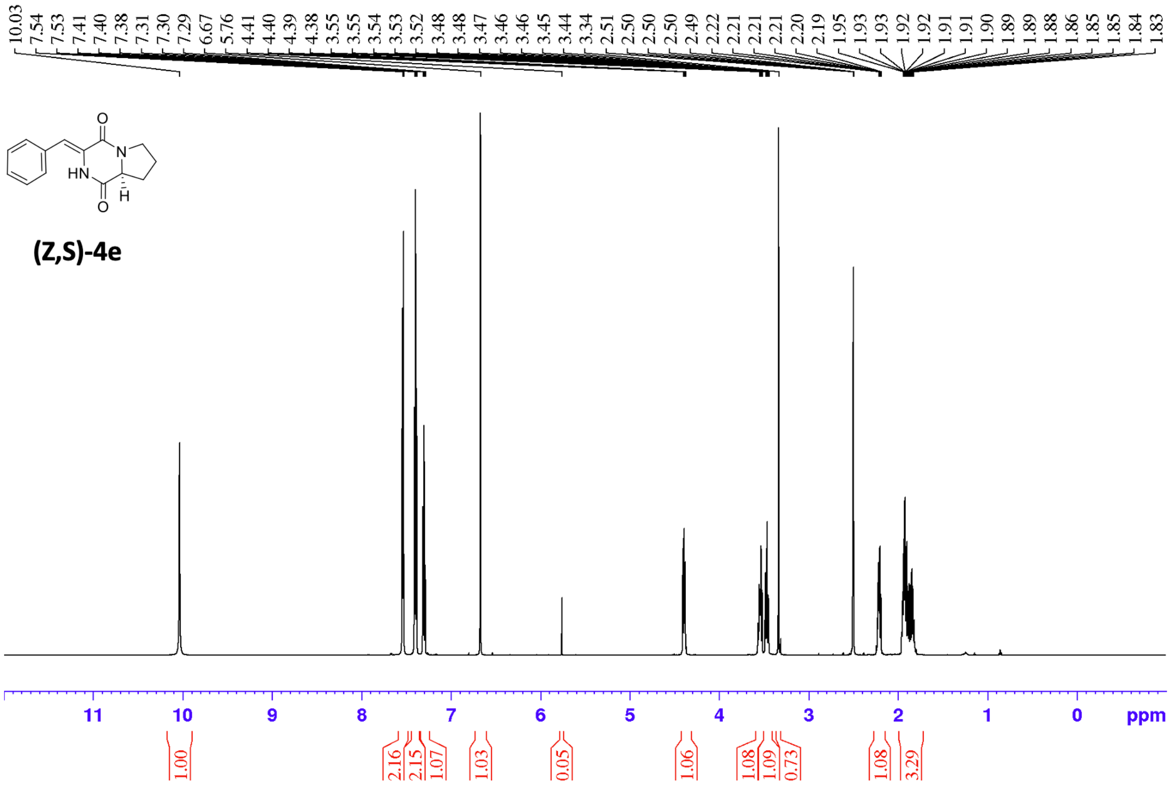

Figure S65. ^{1}H NMR spectrum of (*S,Z*)-**4e** in DMSO-d6

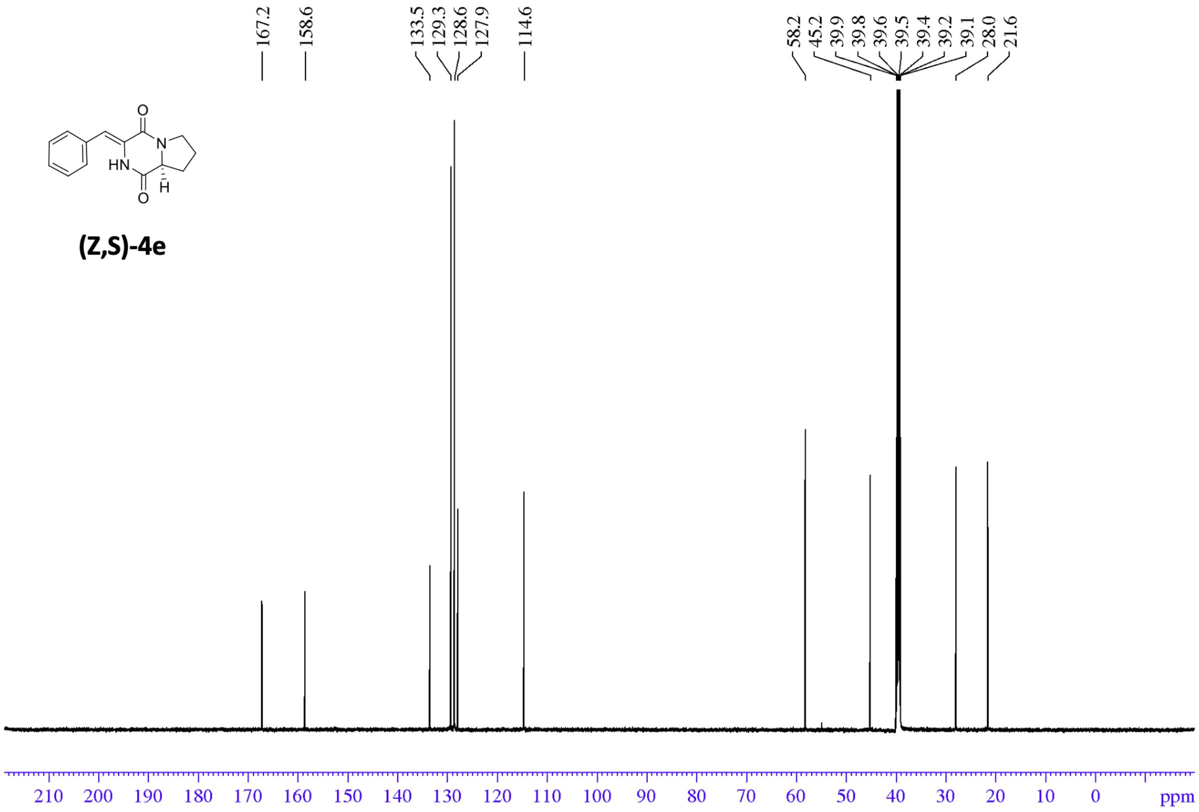

Figure S66. $^{13}C\{^{1}H\}$ NMR spectrum of (*S,Z*)-**4e** in DMSO-d$_6$

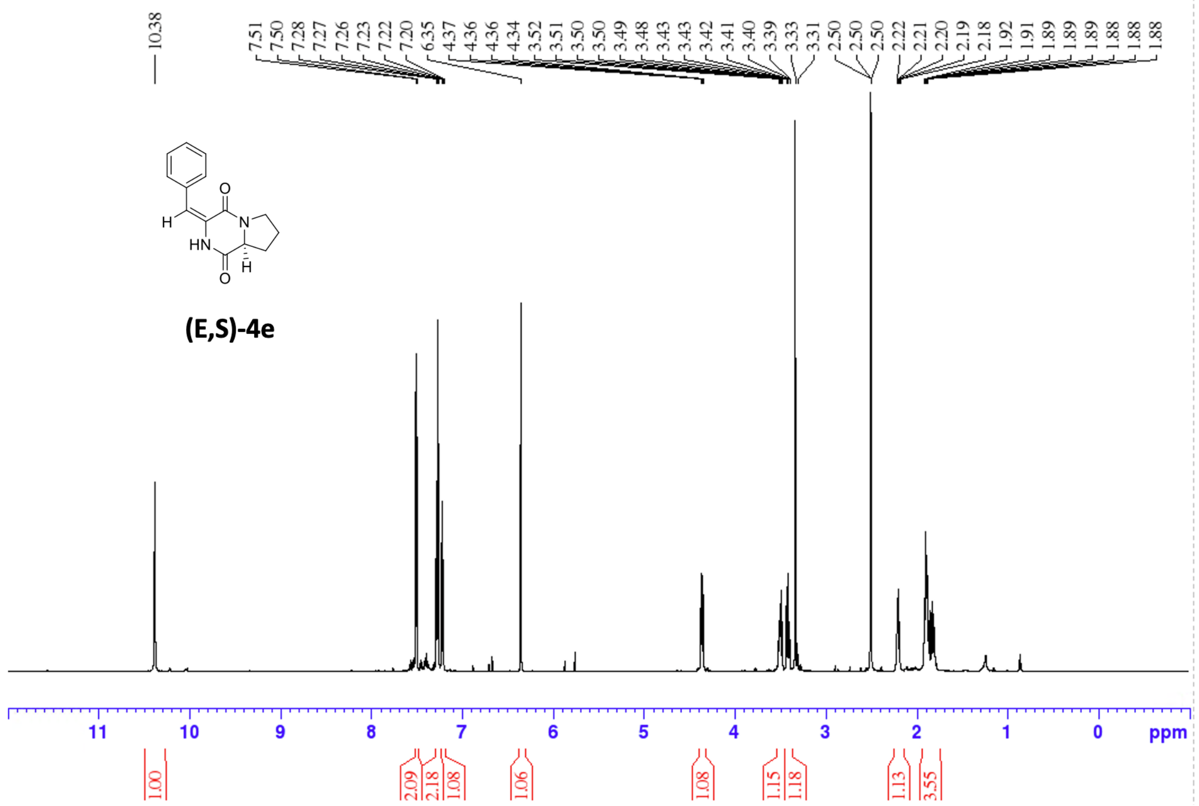

Figure S67. ^{1}H NMR spectrum of (*S,E*)-**4e** in DMSO-d$_6$

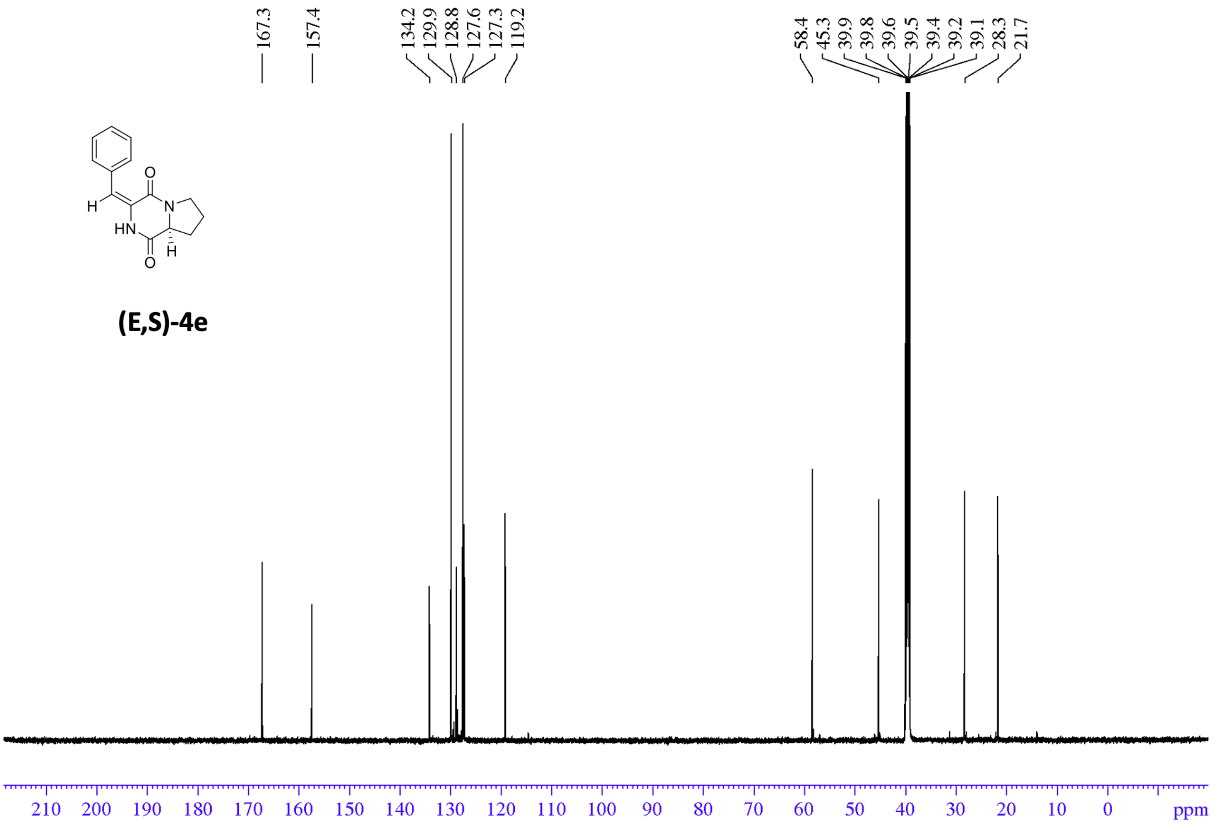

Figure S68. $^{13}C\{^{1}H\}$ NMR spectrum of (*S,E*)-**4e** in DMSO-d$_6$

References

1. Flack, H. D.; Bernardinelli, G., "*Absolute structure and absolute configuration*" *Acta Crystallogr. A.,* **1999,** *55* (5), 908–915. **DOI**: 10.1107/S0108767399004262

2. de Costa, B. R.; He, X.; Linders, J. T. M.; Dominguez, C.; Gu, Z. Q.; Williams, W.; Bowen, W., "*Synthesis and evaluation of conformationally restricted N-[2-(3,4-dichlorophenyl)ethyl]-N-methyl-2-(1-pyrrolidinyl)ethylamines at .sigma. receptors. 2. Piperazines, bicyclic amines, bridged bicyclic amines, and miscellaneous compounds*" *J. Med. Chem.,* **1993,** *36* (16), 2311–2320. **DOI**: 10.1021/jm00068a007

3. Ruzhylo, I.; Sournia-Saquet, A.; Moreau, A.; Delord, T.; Manoury, E.; Poli, R.; Labande, A., "*Heteroleptic Dirhodium(II) Complexes with Redox-Active Ferrocenyl Ligands: Synthesis, Electrochemical Properties, and Redox-Responsive Chemoselectivity in Carbene C−H Insertion*" *Eur. J. Inorg. Chem.,* **2022,** *2022* (12), e202200033. **DOI**: https://doi.org/10.1002/ejic.202200033

4. CrysAlisPro 1.171.39.43d (Rigaku OD, 2015)

5. CrysAlisPro 1.171.39.43d (Rigaku OD, 2015)

6. Sheldrick, G. M., "*A short history of SHELX*" *Acta Crystallogr. A.,* **2008,** *64* (1), 112–122. **DOI**:

7. Sheldrick, G. M., "*SHELXT–Integrated space-group and crystal-structure determination*" *Acta Crystallogr. A.,* **2015,** *71* (1), 3–8. **DOI**:

8. Dolomanov, O. V.; Bourhis, L. J.; Gildea, R. J.; Howard, J. A.; Puschmann, H., "*OLEX2: a complete structure solution, refinement and analysis program*" *J. Appl. Cryst.,* **2009,** *42* (2), 339–341. **DOI**:
9. Sheldrick, G. M., "*Crystal structure refinement with SHELXL*" *Acta Crystallogr. C.,* **2015,** *71* (1), 3–8. **DOI**:
10. Błauż, A.; Rychlik, B., "*Drug-selected cell line panels for evaluation of the pharmacokinetic consequences of multidrug resistance proteins*" *J. Pharmacol. Toxicol. Methods.,* **2017,** *84*, 57–65. **DOI**: 10.1016/j.vascn.2016.11.001
11. Blauz, A.; Wachulec, M.; Rychlik, B., "*Oncostatin M reverses ABCG2-mediated mitoxantrone resistance*" *Biomed. Pharmacother.,* **2024,** *176*, 116861. **DOI**: 10.1016/j.biopha.2024.116861
12. Repetto, G.; del Peso, A.; Zurita, J. L., "*Neutral red uptake assay for the estimation of cell viability/cytotoxicity*" *Nature Protocols,* **2008,** *3* (7), 1125–1131. **DOI**: 10.1038/nprot.2008.75
13. Ianevski, A.; He, L.; Aittokallio, T.; Tang, J., "*SynergyFinder: a web application for analyzing drug combination dose–response matrix data*" *Bioinformatics,* **2020,** *36* (8), 2645–2645. **DOI**: 10.1093/bioinformatics/btaa102
14. Ianevski, A.; Giri, A. K.; Aittokallio, T., "*SynergyFinder 3.0: an interactive analysis and consensus interpretation of multi-drug synergies across multiple samples*" *Nucleic Acids Res.,* **2022,** *50* (W1), W739–W743. **DOI**: 10.1093/nar/gkac382
